# Dual activation of pathways regulated by steroid receptors and peptide growth factors in primary prostate cancer revealed by Factor Analysis of microarray data

**DOI:** 10.1186/1471-2164-6-109

**Published:** 2005-08-17

**Authors:** Juan Jose Lozano, Marta Soler, Raquel Bermudo, David Abia, Pedro L Fernandez, Timothy M Thomson, Angel R Ortiz

**Affiliations:** 1 Bioinformatics Unit, Centro de Biología Molecular "Severo Ochoa" (CSIC-UAM), Universidad Autónoma de Madrid, Cantoblanco, 28049 Madrid, Spain; 2 Department of Physiology and Biophysics, Mount Sinai School of Medicine, One Gustave Levy Pl., New York, NY 10029, USA; 3 Instituto de Biología Molecular, Consejo Superior de Investigaciones Científicas, c. Jordi Girona 18–26, 08034 Barcelona, Spain; 4 Departament de Anatomía Patològica, Hospital Clínic, and Institut de Investigacions Biomèdiques August Pi i Sunyer, c. Villarroel 170, 08036 Barcelona, Spain; 5 Center for Genome Regulation, Barcelona (Spain)

## Abstract

**Background:**

We use an approach based on Factor Analysis to analyze datasets generated for transcriptional profiling. The method groups samples into biologically relevant categories, and enables the identification of genes and pathways most significantly associated to each phenotypic group, while allowing for the participation of a given gene in more than one cluster. Genes assigned to each cluster are used for the detection of pathways predominantly activated in that cluster by finding statistically significant associated GO terms. We tested the approach with a published dataset of microarray experiments in yeast. Upon validation with the yeast dataset, we applied the technique to a prostate cancer dataset.

**Results:**

Two major pathways are shown to be activated in organ-confined, non-metastatic prostate cancer: those regulated by the androgen receptor and by receptor tyrosine kinases. A number of gene markers (HER3, IQGAP2 and POR1) highlighted by the software and related to the later pathway have been validated experimentally *a posteriori *on independent samples.

**Conclusion:**

Using a new microarray analysis tool followed by *a posteriori *experimental validation of the results, we have confirmed several putative markers of malignancy associated with peptide growth factor signalling in prostate cancer and revealed others, most notably ERRB3 (HER3). Our study suggest that, in primary prostate cancer, HER3, together or not with HER4, rather than in receptor complexes involving HER2, could play an important role in the biology of these tumors. These results provide new evidence for the role of receptor tyrosine kinases in the establishment and progression of prostate cancer.

## Background

The phenotype of a cell is determined by its transcriptional repertoire, a result of combinations of transcriptional programs partly set during lineage determination and partly activated in response to intrinsic and extrinsic stimuli. Microarray hybridization experiments permit a quantitative analysis of this transcriptional repertoire in response to defined experimental conditions. A particularly interesting case of study is given by the transcriptional repertoire of human tumors. Here, the objective is usually the search for cancer subtypes for individualized prognosis and/or therapy. The questions most frequently asked are whether samples can be automatically grouped, in the absence of additional information, into biologically relevant phenotypes; and whether transcriptional programs can be unveiled that can explain such phenotypes. It must be noted that this situation (sample clustering and relevant gene extraction) is difficult mainly due to three reasons [1]: the sparsity of the data (samples), the high dimensionality of the feature (gene) space, and the fact that many features are irrelevant or redundant (low signal-to-noise ratio). It has been pointed out that, due to the low signal-to-noise ratio, the quality and reliability of clustering may degrade when using standard hierarchical clustering algorithms or similar approximations [2]. Similarly, model-based clustering methods encounter problems due to the sparsity of the set and its high dimensionality, leading to overfitting during the density estimation process [3]. Additional difficulties are encountered during the selection of features (genes) relevant to the sample cluster structure, since most clustering methods produce non-overlapping gene clusters. This behaviour may distort the extraction of biologically relevant genes in cases where expression patterns overlap several classes of samples or experimental conditions, a reflection of the dependence of the expression of most genes on multiple signals and their participation in more than one regulatory network.

Three main strategies have been taken in sample-based clustering: unsupervised gene selection, interrelated clustering and biclustering [1]. The first views gene selection and sample clustering as basically independent processes, the second dynamically uses the relationship between gene and sample spaces to iteratively apply a clustering and selection engine, while the third tries to cluster both genes and samples at the same time in a reduced space. For the first one, principal components analysis (PCA)[4] has been proposed. PCA, a well known dimensionality reduction technique, has been criticized because the sample projection in the low-dimensional space is not guaranteed to yield optimal sample partitions, particularly when the fraction of relevant genes specific to each cluster is small. As for the second approach, several novel methods have been proposed recently based on various greedy filtering techniques (for a review see [1]), but it has been suggested that they may group the data based on local decisions [1]. Finally, different biclustering methods have also been applied to this situation [5-8], but a difficulty with most biclustering tools is that they generate non-overlapping partitions.

**Figure 1:**
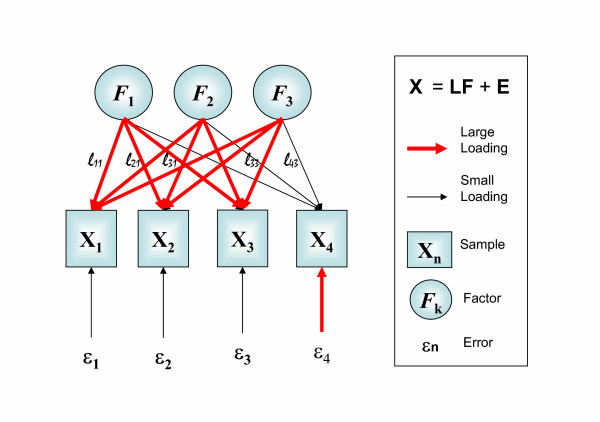
Graphical overview of Q-mode Factor Analysis (FA) [9]. Each sample is described by a vector **x**_**i**_, containing the expression levels for all genes in the chip. The complete expression for all samples is contained in the matrix **X **= {**x**_**i**_}. The expression levels of each sample are assumed to be generated by a linear combination of a small number of underlying transcriptional programs, the latent (non-observable) variables, contained in the set of vectors {**F**_**i**_}, forming matrix **F**. The relative contribution of each program is given by the thickness of the arrows connecting factors and samples, stored in variables *l*_ij_, altogether forming the loading matrix **L**. Each *l*_ij _element can be understood as the correlation coefficient between the expression levels of the sample and the corresponding latent variable. Residuals are kept in vectors {**ε**_**i**_}, giving rise to matrix **E**. Note that small loadings connecting a given sample (i.e., **X**_**4 **_with the factor model implies large residuals.

Here we apply Factor Analysis (FA) [9], a multivariate tool related to PCA, coupled to clustering algorithms in sample space, *t*-test scores in gene space and data mining procedures. Q-mode (i.e. in sample space) FA is a latent variable modelling tool [9] that assumes that the observed gene expression levels are the result of a linear combination of an unknown number of independent underlying global transcriptional programs, called latent variables or factors (Figure 1). The contribution of each factor to the expression levels of the genes in each sample is given by the elements of the loadings matrix (arrows in Figure 1). Each sample contains, in addition, a given amount of expression that cannot be modelled by the latent variables, for example due to the presence of noise. FA models the covariance of a data matrix, as opposed to PCA, which attempts to summarize the total variance. Covariance in the mRNA expression levels has been shown to occur in proteins involved in related pathways and functions, as well as in proteins co-locating to the same organuli in the cell, and may be indicative of common regulatory mechanisms at the expression level[10]. By contrast, the specific variance in the expression of a given gene, not associated with the rest of the genes in the sample, is most likely related to artefacts in the chip or in data handling. We couple FA dimensionality reduction to clustering algorithms [11] to obtain clusters in sample space. For gene extraction, a multiple-testing corrected *t*-test (the so-called *q*-value) is employed. Finally, the genes assigned to each cluster are used for the detection of pathways predominantly activated in that cluster by finding statistically significant the GO [12] or GenMAPP terms associated to each cluster.

We first tested the approach by using a published dataset of microarray experiments in yeast [13], and then applied it to the analysis of human prostate cancer samples [14]. The yeast dataset is particularly relevant because the biochemistry of *S. cereviseae *is relatively well understood in comparison with other eukaryots, and the data set has been previously analyzed with other clustering techniques. From the application to the prostate cancer dataset, a number of significant gene outcomes highlighted by the algorithm have been corroborated experimentally *a posteriori *by expression analysis on an independent set of samples. The biological interpretation of the results lead us to propose that two major pathways are predominantly activated in organ-confined, non-metastatic prostate cancer: those regulated by androgen receptor and by receptor tyrosine kinases. We close this paper by discussing the implications of these findings.

## Results and Discussion

### Testing FADA with the yeast expression dataset

Our procedure is coded in a software package, FADA. We first tested FADA by analyzing the dataset of Gasch et al [13], who studied the transcriptional responses of *S. cerevisiae *to a variety of stress stimuli. The main results are in Table 1, and the genes most significantly associated to the clusters are in Table 1 of the Supporting Information. We discuss in what follows only the most salient features of the analysis for this set, corresponding to clusters 1, 2, 6, 8 and 16, from a biological viewpoint.

**Table 1:** Results of the analysis of the yeast dataset [13]. The different clusters found by FADA are shown, together with the significant GO terms associated to them. The samples belonging to each one of the clusters are also shown. The first column shows the cluster number; the second shows the conditions associated to that cluster; columns 3 to 5 show the *Z*-score of the GO terms associated to the cluster (see Methods) at the Cellular Component (CC), Biological Process (BP) and Molecular Function (MF) levels; columns 6 to 8 show the corresponding GO terms.

**C**	**CONDITIONS**	**Z(CC)**	**Z(BP)**	**Z(MF)**	**GO(CC)**	**GO(BP)**	**GO(MF)**
**1**	Heat_Shock_05_minutes_hs.1, Heat_Shock_10_minutes_hs.1 Heat_Shock_15_minutes_hs.1, Heat_Shock_20_minutes_hs.1 Heat_Shock_30_minutes_hs.1, Heat_Shock_40_minutes_hs.1 Heat_Shock_60_minutes_hs.1, Heat_Shock_80_minutes_hs.1 Heat_Shock_015_minutes_hs.2, Heat_Shock_030minutes_hs.2 heat_shock_17_to_37._20_minutes, heat_shock_21_to_37._20_minutes heat_shock_25_to_37._20_minutes, heat_shock_29_to_37._20_minutes heat_shock_33_to_37._20_minutes 29C_to_33C_._5_minutes, 29C_to_33C_._15_minutes 29C_.1M_sorbitol_to_33C_._1M_sorbitol_._5_minutes 29C_.1M_sorbitol_to_33C_._1M_sorbitol_._15_minutes 29C_.1M_sorbitol_to_33C_._.NO_sorbitol_._5_minutes dtt_240_min_dtt.2 1M_sorbitol_._5_min 1M_sorbitol_._15_min 1M_sorbitol_._30_min 1M_sorbitol_._45_min_ DBY7286_37degree_heat_._20_min DBYmsn2.4._37degree_heat_._20_min DBYmsn2.4_.real_strain._._37degrees_.20_min. DBYyap1._37degree_heat_._20_min_.redo. DBYyap1_._37degree_heat_.repeat., DBYyap1_._0.32_mM_H2O2_.20_min. Msn2_overexpression_.repeat. Msn4_overexpression	3.25	5.39	1.16	nucleolus (325/88;0.81E-006)	ribosome biog. & ass. (271/75; 0.57E-007) **response to stress **(214/52; 0.26E-003)	
**2**	constant_0.32_mM_H2O2_.10_min._redo constant_0.32_mM_H2O2_.20_min._redo constant_0.32_mM_H2O2_.30_min._redo constant_0.32_mM_H2O2_.40_min._rescan constant_0.32_mM_H2O2_.50_min._redo constant_0.32_mM_H2O2_.60_min._redo 1.5_mM_diamide_.5_min. 1.5_mM_diamide_.10_min. 1.5_mM_diamide_.20_min. 1.5_mM_diamide_.30_min. 1.5_mM_diamide_.40_min. 1.5_mM_diamide_.50_min. 1.5_mM_diamide_.60_min. 1.5_mM_diamide_.90_min. DBY7286_._0.3_mM_H2O2_.20_min. DBYmsn2msn4_.good_strain._._0.32_mM_H2O2 DBYmsn2.4_.real_strain._._0.32_mM_H2O2_.20_min. DBYyap1._._0.3_mM_H2O2_.20_min.	0.39	9.71	11.40		protein catabolism (114/28; 0.74E-017) cell homeostasis (54/8; 0.10E-003)	**peptidase activity **(125/25; 0.17E-012) **oxidored. Act. **(263/30; 0.36E-008)
**3**	2.5 mM_DTT_045_min_dtt.1 2.5 mM_DTT_060_min_dtt.1 2.5 mM_DTT_090_min_dtt.1 2.5 mM_DTT_120_min_dtt.1 2.5 mM_DTT_180_min_dtt.1 dtt_120_min_dtt.2	6.55	2.53	1.86	endoplasmic ret. (353/27; 0.11E-008)		
**4**	constant_0.32_mM_H2O2_.80_min._redo constant_0.32_mM_H2O2_.100_min._redo constant_0.32_mM_H2O2_.120_min._redo constant_0.32_mM_H2O2_.160_min._redo	1.25	0.63	1.58			
**5**	37_deg_growth_ct.1	NA	NA	NA			
**6**	Nitrogen_Depletion_8_h Nitrogen_Depletion_12_h Nitrogen_Depletion_1_d Nitrogen_Depletion_2_d Nitrogen_Depletion_3_d Nitrogen_Depletion_5_d	7.11	5.30	0.61	plasma membrane (197/16; 0.86E-005) extracellular region (19/4; 0.85E-004)	**transcription **(225/15 0.57E-003)	
**7**	diauxic_shift_timecourse_18.5_h diauxic_shift_timecourse_20.5_h YPD_6_h_ypd.2 YPD_8_h_ypd.2 YPD_10_h_ypd.2 YPD_12_h_ypd.2 YPD_1_d_ypd.2 YPD_2_d_ypd.2 YPD_3_d_ypd.2 YPD_5_d_ypd.2 YPD_stationary_phase_12_h_ypd.1 YPD_stationary_phase_1_d_ypd.1 YPD_stationary_phase_2_d_ypd.1 YPD_stationary_phase_3_d_ypd.1 YPD_stationary_phase_5_d_ypd.1 YPD_stationary_phase_7_d_ypd.1 YPD_stationary_phase_13_d_ypd.1 YPD_stationary_phase_22_d_ypd.1 YPD_stationary_phase_28_d_ypd.1 ethanol_vs._reference_pool_car.1 YP_ethanol_vs_reference_pool_car.2	9.91	####	5.51	ribosome (368/126; 0.20E-010) peroxisome (52/22; 0.66E-004)	protein biosynthesis (493/168; 0.12E-012) **vitamin metabolism **(48/20; 0.27E-003)	structural mol act (359 /119; 0.13E-006)
**8**	aa_starv_0.5_h aa_starv_1_h aa_starv_2_h aa_starv_4_h aa_starv_6_h Nitrogen_Depletion_30_min. Nitrogen_Depletion_1_h Nitrogen_Depletion_2_h Nitrogen_Depletion_4_h	3.60	####	4.66	peroxisome (52/6; 0.14E-003) plasma membrane (197/17; 0.35E-006)	**aminoacid metab **(173/42; 0.24E-031)	transporter act (343/27; 0.12E-004) lyase activity (97/12; 0.24E-004)
**9**	33C_vs._30C_._90_minutes dtt_480_min_dtt.2 steady_state_36_dec_C_ct.2 steady_state_36_dec_C_ct.2_.repeat_hyb._	0.12	-1.42	-0.24			
**10**	dtt_060_min_dtt.2 YP_galactose_vs_reference_pool_car.2 YP_raffinose_vs_reference_pool_car.2	1.43	0.12	-1.53			
**11**	Diauxic_Shift_Timecourse_._0_h diauxic_shift_timecourse_9.5_h diauxic_shift_timecourse11.5_	2.86	1.16	0.44	vacuole (140/6; 0.63E-003)		
**12**	YPD_stationary_phase_2_h_ypd.1 YPD_stationary_phase_4_h_ypd.1	-0.39	3.11	2.06		electron transport (14/1; 0.91E-003)	
**13**	diauxic_shift_timecourse_13.5_h diauxic_shift_timecourse_15.5_h YPD_stationary_phase_8_h_ypd.1	-0.51	-0.27	-0.20			
**14**	1M_sorbitol_._60_min 1M_sorbitol_._90_min 1M_sorbitol_._120_min	1.21	2.46	1.33		cell cycle (115/4; 0.11E-003)	
**15**	YPD_2_h_ypd.2 YPD_4_h_ypd.2 YAP1_overexpression	-0.34	0.20	0.56			
**16**	1_mM_Menadione_.10_min.redo 1_mM_Menadione_.20_min._redo 1_mM_Menadione_.30_min._redo 1mM_Menadione_.40_min._redo 1_mM_Menadione_.50_min.redo 1_mM_Menadione_.80_min._redo 1_mM_Menadione_.105_min._redo 1_mM_Menadione_.120_min.redo 1_mM_Menadione_.160_min._redo	4.71	6.25	2.67	mitochondrion (732/88; 0.49E-003) **Golgi apparatus **(90/17; 0.63E-003)	vesicle-med. Transp. (190/31; 0.84E-004)	
**17**	Heat_Shock_000_minutes_hs.2 Heat_Shock_000_minutes_hs.2.1 Heat_Shock_000_minutes_hs.2.2 37C_to_25C_shock_._15_min 37C_to_25C_shock_._30_min 37C_to_25C_shock_._45_min 37C_to_25C_shock_._60_min 37C_to_25C_shock_._90_min dtt_000_min_dtt.2 dtt_015_min_dtt.2 dtt_030_min_dtt.2 steady_state_21_dec_C_ct.2 steady_state_25_dec_C_ct.2 steady_state_29_dec_C_ct.2	5.33	####	5.49	ribosome (368/145; 0.26E-007) nucleolus (325/138; 0.15E-009)	ribosome biog & ass (271/121; 0.87E-011) RNA metabolism (382/148; 0.14E-007) **protein biosynthesis **(493/194 0.12E-010)	structural mol. act. (359/123; 0.11E-003) RNA binding (268/96; 0.93E-004)
**18**	YP_fructose_vs_reference_pool_car.2 YP_glucose_vs_reference_pool_car.2 YP_mannose_vs_reference_pool_car.2 YP_sucrose_vs_reference_pool_car.2	2.12	0.31	0.32	mitochondrial membr (136/7; 0.59E-004)		
**19**	Hypo.osmotic_shock_._15_min Hypo.osmotic_shock_._30_min Hypo.osmotic_shock_._45_min Hypo.osmotic_shock_._60_min	3.08	5.06	3.67	bud (59/6; 0.40E-003) nucleolus (325/21; 0.525E-005)	ribosome biog & ass (271/24; 0.12E-007) RNA metabolism (382/22; 0.86E-004	
**20**	Heat_Shock_060_minutes_hs.2	NA	NA	NA			
**21**	17_deg_growth_ct.1 21_deg_growth_ct.1 25_deg_growth_ct.1 29_deg_growth_ct.1	1.67	1.54	-1.34			
**22**	steady_state_15_dec_C_ct.2 steady_state_17_dec_C_ct.2	0.51	-1.14	0.48			
**23**	2.5mM_DTT_005_min_dtt.1 2.5mM_DTT_015_min_dtt.1 2.5mM_DTT_030_min_dtt.1	0.03	1.28	0.32			
**24**	galactose_vs._reference_pool_car.1 glucose_vs._reference_pool_car.1 mannose_vs._reference_pool_car.1 raffinose_vs._reference_pool_car.1 sucrose_vs._reference_pool_car.1 steady_state_33_dec_C_ct.2	8.69	5.93	6.57	cell cortex (39/7; 0.97E-003) cytoplasmic vesicle (52/11; 0.15E-004) bud (59/10; 0.27E-003) endomembrane syst (76/15; 0.21E-005)	cytokinesis (52/10; 0.25E-003) nuclear org & biog (105/16; 0.20E-003) vesicle-med transp (190/23; 0.55E-003)	helicase activity (71/13; 0.21E-004)
**25**	29C_to_33C_._30_minutes 29C_.1M_sorbitol_to_33C_._1M_sorbitol_._30_minutes 29C_.1M_sorbitol_to_33C_._.NO_sorbitol_._15_minute 29C_.1M_sorbitol_to_33C_._.NO_sorbitol_._30_minute Hypo.osmotic_shock_._5_min steady.state_1M_sorbitol	0.39	2.96	3.27		DNA metabolism (221/8; 0.78E-003)	DNA binding (146/7; 0.21E-003)
**26**	Heat_Shock_005_minutes_hs.2	NA	NA	NA			

(a) GO terms discussed in the text are shown in bold. Together with each GO term, we show the number of genes corresponding to that term; the number of genes of that term in the cluster; and the corresponding *P*-value, according to the hipergeometric distribution.

(b) NA: Data Not Available. No significant genes (according to the *q*-value cutoff) could be found.

Cluster 1 encompasses responses to heat shock, DTT (late), sorbitol (early response), stationary culture (late), and overexpression of Msn2p and Msn4p. The significant GO terms [12] automatically detected by FADA indicate that this grouping is related to a common environmental stress response (Table 1) (ESR or CER in the case of *S. cerevisae*, CESR in the case of *S. pombe *[13,15,16]). Inspection of the top selected genes (Table 1, supplementary material) confirms this assignment, as most of them are known to be transcriptionally regulated through the stress response element (STRE), recognized by Msn2p and Msn4p [17,18]. Thus, Cluster 1 corresponds largely to a *"core" *ESR, induced by a variety of stimuli, including *"early" *time points of osmotic stress and *"late" *time points of DTT treatment and stationary culture. A relatively late induction of ESR by DTT has been noted previously, with suggestions that ESR could be a secondary response to the exposure of this reducing agent [13]. Conversely, hyperosmotic shock is known to induce a rapid and strong expression of ESR [13,15].

Clusters 2 and 16 correspond to responses to three oxidizing agents: hydrogen peroxide, which generates peroxides and hydroxyl radicals; menadione, a generator of superoxide; and diamide, a thiol reducing agent. FADA groups together responses to H_2_O_2 _and diamide (Cluster 2), while defining a distinct group for responses to menadione (Cluster 16). It is well known that several organisms use distinct sensing and response systems to discriminate among different degrees of oxidative injury. In *S. pombe*, reponses to low concentrations of H_2_O_2 _and to diamide depend on the b-Zip transcription factor pap1, while reponses to higher concentrations of H_2_O_2 _utilize a different transcription factor, atf1 [19]. The *S. cerevisiae *homologue of *S. pombe *pap1 is Yap1p, a transcription factor regulated by oxidation, formation of intramolecular disulfide bonds at its carboxy terminus and nuclear translocation upon exposure to H_2_O_2 _and diamide [20-22]. On the other hand, of the b-ZIP proteins in *S. cerevisiae*, Yap3p is most similar to atf1 in its carboxy terminus, suggesting that both atf1 and Yap3p could be subject to a similar redox regulation. Interestingly, YAP1 is upregulated in Cluster 2 (H_2_O_2 _and diamide), but not in Cluster 8 (menadione), while Yap3 is upregulated in the latter Cluster (Table 1 of Supplementary Data). Moreover, several of the genes most relevant to Cluster 2 are known to respond to mild oxidative stress, and are controlled by Yap1p [23]. The statistically significant GO-terms selected are related to *"oxidoreductase" *and *"peptidase" *activities. This includes genes regulating the thioredoxin and glutathione biosynthesis, genes for heat shock proteins, and a large number of genes involved in proteasome function and ubiquitin-dependent protein degradation (Table 1 of Supplementary Material).

Cluster 6 includes cultures at late times of nitrogen starvation. Many of the relevant genes in this group code for enzymes for the utilization and enhanced transport of poor nitrogen sources, such as allantoin or urea (Table 1 of Supplementary Material). Other upregulated genes include those required for different stages of meiosis (chromosome pairing, recombination and segregation; anaphase; or nucleokinesis), sporulation, autophagy, or genes that regulate vesicle and peroxisome structure and dynamics. Among these genes are also transcriptional regulators with major roles in the control of several of these processes, such as *UGA3*, *DAL81 *(allantoin metabolism), or *IME1*, *RIM101 *and *SPO1 *(meiosis and sporulation). This is consistent with the development of a classical response to nitrogen starvation in the absence of fermentable carbon sources, which leads to meiosis and sporulation [24-27]. FADA also suggests that this response to nitrogen starvation becomes most prominent at relatively late times, when it can be distinguished from the early, relatively non-specific response to nutrient deprivation [25,26]. In fact, FADA finds *"transcription" *and *"sporulation" *as significant GO-terms (Table 1).

Cluster 8 aggregates samples from early stages of both early response to aminoacid and nitrogen starvation. FADA finds a significant overrepresentation of genes for amino acid biosynthetic pathways (Table 1), consistent with the fact that deprivation of nutrients, including nitrogen and carbon sources, is recognized by several sensing systems regulating rapamycin-sensitive TOR kinase [28]. This lipid-dependent kinase derepresses translation of the GATA transcription factor Gcn4p [29,30], which controls expression of many genes, including enzymes involved in amino acid biosynthesis [31]. Thus, the selection of genes in Cluster 8 is consistent with known Gcn4p-dependent responses to nutrient and nitrogen starvation [31].

Altogether, these results indicate that the automatic analysis provided by FADA yields results consistent with the known biochemistry of yeast.

### Application of FADA to the prostate cancer dataset

**Figure 2:**
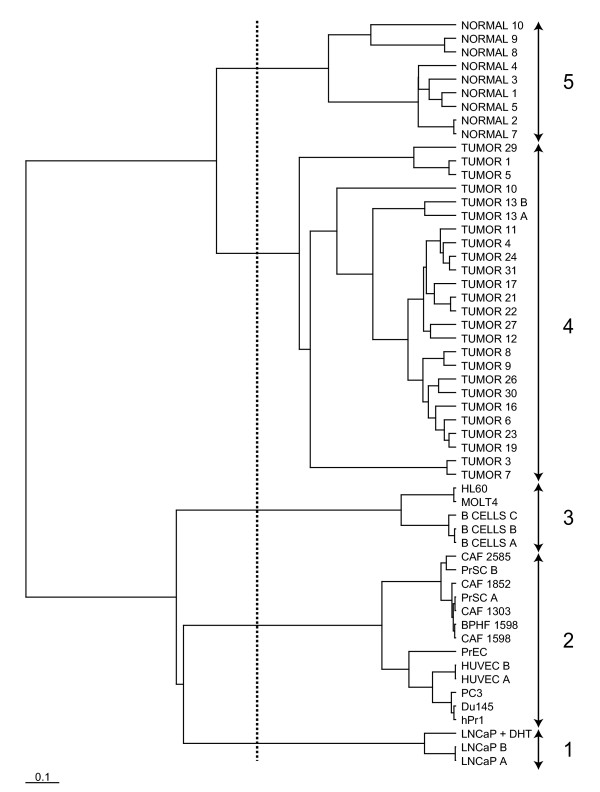
Dendrogram for the Welsh dataset [14]. The dashed line indicates the thresholding used to define the clusters.

We next applied FADA to the dataset published by Welsh et al. [14], for the analysis of transcripts associated with prostate cancer. Samples were classified into two major branches: samples from cultured cells, and samples from tissues, which in turn could be further bifurcated into two well-supported branches, one corresponding to samples enriched for carcinomatous cells and one for non-neoplastic prostate cells (Figure 2). The first-level grouping into cultured *vs*. non-cultured samples most likely reflects the profound impact of culturing procedures on the transcriptional profiles of the different cell types. Within the cultured cells subgroup, samples were generally clustered according to cell type, with haematopoietic cell lines forming well-clustered groups and epithelial and fibroblastic prostate-derived cells clustering together with endothelial cells. A separate cluster was formed by the androgen-sensitive epithelial cell line LNCaP, the prostate cancer cell lines included in the study. The genes most significantly contributing to each sample cluster were analyzed for their participation in the pathways contained both in GenMAPP [32], and GO (Tables 2 and 3). Since pathway categorization is a difficult problem, as partition of the global interaction network in "parts" inevitably introduces artefacts, we also proceeded to a detailed, gene-by-gene inspection of the most discriminative genes based on inspection of literature data.

**Table 2:** Results of the analysis of the Welsh dataset for up-regulated genes. The different sample clusters found by FADA are shown, together with the significant GO and GenMAPP terms associated to them. The first column shows the cluster number; the second shows the samples associated to that cluster; columns 3 and 4 show the *z*-score of the GenMAPP and GO terms associated to the cluster (see Methods); columns 5 to 8 show the corresponding GenMAPP and GO terms selected.

**C**	**SAMPLES**	**Z(GM)**	**Z(GO)**	**GENMAPP**	**GO(MF)**	**GO(BP)**	**GO(CC)**
**1**	LNCaP_A, LNCaP_B, LNCaP_+_DHT	7.22	5.09 -0.39 2.59	RNA_transcription_React. (2.40e-03) Electron_Transport_Chain (5.74e-10)	oxidoreductase activity (1.58e-04) carrier activity (1.87e-04) ATPase activity, coupled to transmembrane movement of substances (4.23e-03) transcriptional activator activity (2.09e-03)		intracellular organelle (5.48e-04)
**2**	CAF_1598, BPHF_1598 CAF_1303, CAF_1852 PrSC_A, PrSC_B, CAF_2585, Du145, PC3, HUVEC_A, HUVEC_B, hPr1, PrEC	3.83	4.66 2.74 5.13	Hypertrophy_model (8.97e-03) Proteasome_Degradation (1.44e-08) Cell_cycle_KEGG (7.86e-03) Pentose_Phosphate_Pathway (4.89e-03)	Enzyme inhibitor activity (4.96e-04) hydrolase activity (9.26e-03) small protein conjugating enzyme activity (3.54e-04) structural constituent of cytoskeleton (2.73e-03) nucleotide binding (1.17e-05)	regulation of cellular process (4.31e-03) cellular physiological process (1.93e-04)	signalosome complex (7.27e-03) membrane coat adaptor complex (4.24e-03) tubulin (5.06e-06) proteasome complex (sensu Eukaryota)(3.69e-07) Arp2/3 protein complex (4.19e-05)
**3**	B_CELLS_A, B_CELLS_B B_CELLS_C, MOLT4, HL60	4.27	0.42 0.18 -0.04	mRNA_processing_React. (2.10e-03) G1_to_S_cell_cycle_React. (9.25e-05) Cell_cycle_KEGG (1.94e-04) Small_ligand_GPCRs (5.52e-03) Ovarian_Infertility_Genes (5.19e-03) GPCRDB_Class_C_Metabotropic_glutamate_pheromone (4.72e-04)			
**4**	T4, T7 T3, T5, T1, T27, T10, T9, T13A, T13B, T22, T12, T29, T8, T31, T30, T26, T19, T16, T23, T6, T24, T21, T11, T17	2.06	1.93 0.39 0.76	Fatty_Acid_Degradation (8.48e-03) Hypertrophy_model (3.34e-03) Eicosanoid_Synthesis (3.56e-03)	steroid binding (7.57e-03) isomerase activity (7.99e-04) vitamin binding (6.99e-04)		
**5**	N2, N1, N5, N3, N9, N8, N7, N10, N4	6.86	2.52 -0.31 -0.40	Smooth_muscle_contraction (6.55e-03) Calcium_reg_in_card_cells (3.97e-03)	channel or pore class transporter activity (1.75e-03) structural constituent of cytoskeleton (2.75e-04)		

(a) GO or GenMAPP terms are discussed in the text. Together with each term, we show the corresponding *P*-value, according to the hipergeometric distribution (see Methods).

(b) The Z(GO) column shows the Z-scores corresponding to Molecular Function (MF), Biological Process (BP), and Celular Component (CC), respectively, obtained for each cluster. The Z(GM) column refers to the Z-score corresponding to the GenMAPP terms.

**Table 3:** Results of the analysis of the Welsh dataset for down-regulated genes. The different sample clusters found by FADA are shown, together with the significant GO and GenMAPP terms associated to them. The first column shows the cluster number; the second shows the samples associated to that cluster; columns 3 and 4 show the *Z*-score of the GenMAPP and GO terms associated to the cluster (see Methods); columns 5 to 8 show the corresponding GenMAPP and GO terms selected.

**C**	**SAMPLES**	**Z(GM)**	**Z(GO)**	**GENMAPP**	**GO(MF)**	**GO(BP)**	**GO(CC)**
**1**	LNCaP_A, LNCaP_B, LNCaP_+_DHT	0.36	-0.10 -0.54 4.09				extracellular space (3.74e-04) MHC protein complex (4.71e-04)
**2**	CAF_1598, BPHF_1598, CAF_1303, CAF_1852, PrSC_A, PrSC_B, CAF_2585, Du145, PC3, HUVEC_A, HUVEC_B, hPr1, PrEC	2.33	2.43 0.68 -0.50	Hs_GPCRDB_Other (1.96e-03) Hs_Ribosomal_Proteins (1.03e-08)	structural constituent of ribosome (2.57e-05) SH3/SH2 adaptor activity (6.48e-03) nucleic acid binding (7.71e-04) oxygen transporter activity (6.93e-03) nucleobase, nucleoside, nucleotide and nucleic acid transporter activity (6.93e-03)		
**3**	B_CELLS_A, B_CELLS_B, B_CELLS_C, MOLT4, HL60	0.91	0.85 -0.65 -0.91				
**4**	T4, T7, T3, T5, T1, T27, T10, T9, T13A, T13B, T22, T12, T29, T8, T31, T30, T26, T19, T16, T23, T6, T24, T21, T11, T17	4.30	1.04 1.86 7.71	G1_to_S_cell_cycle_React (7.76e-04) Glycolysis_and_Gluconeogenesis (4.02e-04) Cell_cycle_KEGG (7.03e-08) DNA_replication_Reactome (2.01e-03)			signalosome complex (5.23e-04) intracellular (4.66e-04) tubulin (2.97e-03) proteasome complex (sensu Eukaryota) (2.47e-04) proton-transporting ATP synthase complex (9.01e-04) Arp2/3 protein complex (5.23e-04)
**5**	N2, N1, N5, N3, N9, N8, N7, N10, N4	-0.21	-0.65 3.83 -0.53			metabolism (3.90e-04)	

(a) GO or GenMAPP terms are discussed in the text. Together with each term, we show the corresponding *P*-value, according to the hipergeometric distribution (see Methods).

(b) The Z(GO) column shows the *Z*-scores corresponding to Molecular Function (MF), Biological Process (BP), and Cellular Component (CC), respectively, obtained for each cluster. The Z(GM) column refers to the Z-score corresponding to the GenMAPP terms.

**Figure 3:**
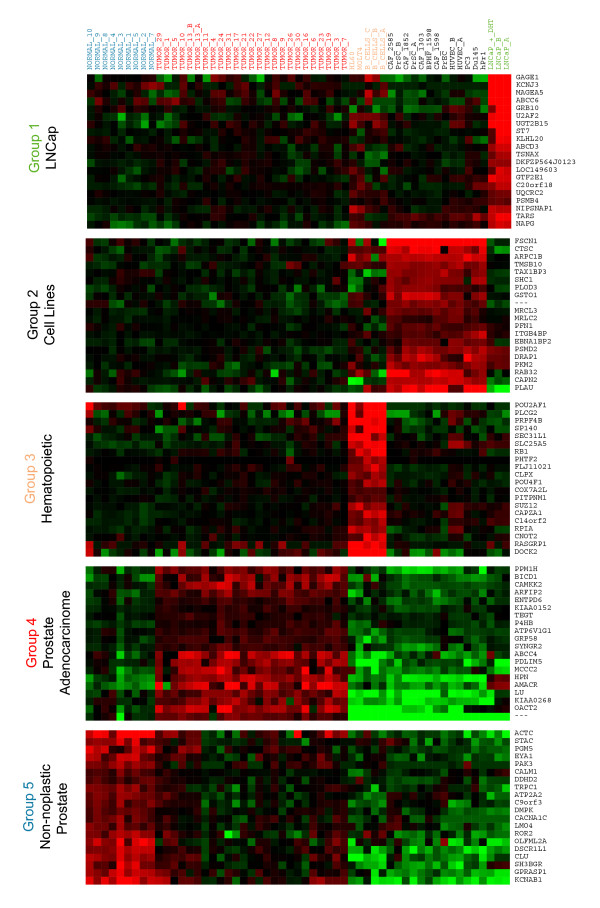
Expression levels for the 20 most relevant genes selected in each cluster for the Welsh dataset. Gene descriptions can be found in Table 2 of the Supporting Information. A) (See Figure 3) Up-regulated; B) Down-regulated. (See Figure 4)

**Figure 4:**
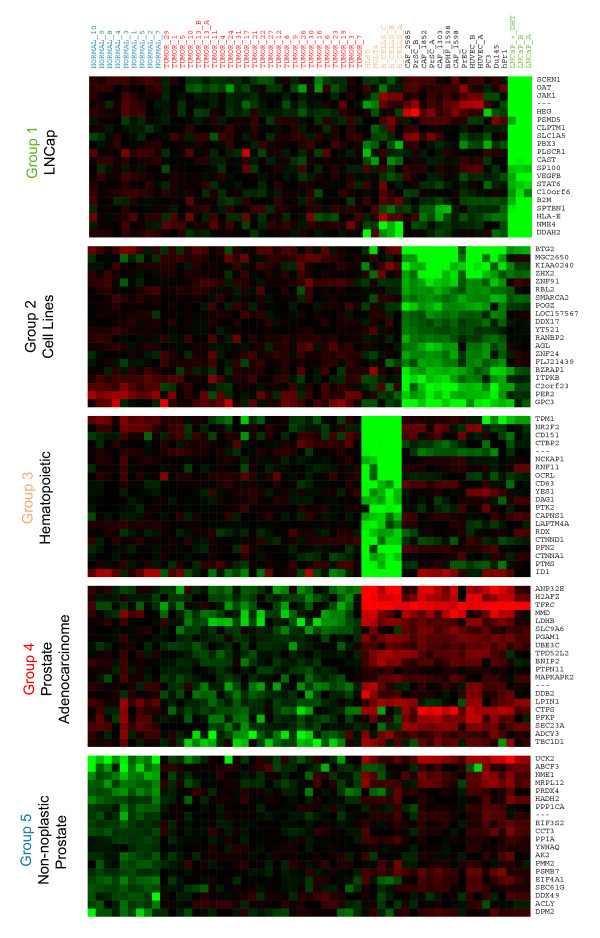


Cluster 1 corresponds to the LNCaP cluster. It is placed in a branch distinctly separated from the rest of the cultured prostatic cells. LNCaP cells were originally derived from metastatic prostate cells, presumably of epithelial origin [33] and respond to androgens through its cognate receptor [34]. FADA found significant overrepresentation of upregulated genes coding for proteins that participate in electron transport and ATP generation, both when using GenMAPP and GO annotations (Figure 3, 4 and Table 2). Other sets of genes likely relevant to the LNCaP cluster, but not highlighted in the pathway mapping protocol, are those for proteins in steroid metabolism and signalling, such UDP glycosyltransferases B15 (Table 2 of the Supporting Information). Cluster 2 includes mesenchimal, epithelial, and endothelial cells. This cluster shows a bias for genes and pathways involved in ubiquitin and proteasome-dependent protein degradation, cell cycle regulation, inflammatory responses and cell-matrix interaction. Cluster 3 (hematopoietic cells) showed a significant bias in genes and pathways involved in cell cycle regulation and RNA processing. The selected genes included known markers of differentiation of B cell, T cell or myelomonocytic lineages. Examples are genes for immunoglobulin, histocompatibility antigens, haematopoietic-specific cytokines and their receptors, and regulatory proteins known to play significant roles in such lineages in processes such as signal transduction or cytoskeletal dynamics.

Regarding Cluster 4 (prostate tumor tissue), GenMAPP mapping finds significant overexpression of enzymes related to fatty acid metabolism (Table 2). Other genes and KEGG pathways with a significantly biased association with cluster 4 are those for ribosomal function and fatty acid synthesis (Table 2 of the Supporting Information). The upregulation of these two functions in prostate cancer has been noted previously [14,35]. In addition, GO mapping finds overrepresentation of genes for proteins directly involved in steroid receptor recognition, including androgen receptor and estrogen receptor β. This is confirmed by a survey of the list of selected genes, where one can find a number of proteins involved in steroid signalling, including the coactivators GRIP1 and NRIP1, and genes that have been described as transcriptional targets of these pathways [36], such as the secreted proteases KLK2 and KLK3, and protein IQGAP, involved in cytoskeletal dynamics [37], or the enzymes fatty acid CoA-ligase or androgen-regulated short chain dehydrogenase (Table 2 of the Supporting Information). A second group of genes significantly contributing to this cluster are those for cell surface polypeptide growth factor receptors, associated signalling molecules and regulators, and known transcriptional targets for these pathways. These include the receptor tyrosine kinase partner ERBB3 (HER3), the calmodulin-dependent kinase activator CAMKK2, or the signalling modulators RAPGA1 and PDE3B (Table 2 of the Supporting Information).

Finally, the highest ranking genes for samples from normal prostate tissue (Cluster 5) correspond, according to GO, to proteins involved in the control of cytoskeletal architecture and dynamics in muscle cells (Table 2). GenMAPP finds a significant overrepresentation of muscle-associated functions. The implication is that, in these experiments, normal prostate tissue samples possibly are strongly enriched for muscle cells. This strong overrepresentation of genes corresponding to a smooth muscle phenotype suggests that the non-neoplastic tissues used correspond to areas of prostate hyperplasia or adenoma derived from the transition zone, in which smooth muscle cells are often major contributors [38]. In practical terms, this suggests that these experiments may be used with caution in the comparison of tumor epithelial cells with corresponding normal epithelial counterparts.

In recent years, several transcriptional profiling studies have been performed in prostate cancer, aimed at the identification of novel tumor markers [14,39-41] or prognostic signatures [42-44]. So far, only one study has systematically searched for overrepresented biochemical pathways in a meta-analysis of four previously published prostate cancer transcriptional profiling studies [45]. This study used KEGG as reference pathway database, which is biased towards metabolic pathways [46]. Our study, however, focuses on GenMapp and GO terms, and therefore on the identification of signalling pathways.

### Signalling pathways in prostate cancer and their experimental validation

In order to validate the pathways found to be overrepresented in prostate tumor samples, we used real-time RT-PCR. We chose for our analysis the genes for hepsin, KLK3 (PSA), ERBB3 (HER3), IQGAP2, and POR/ARFAPTIN2. Hepsin was found to be overexpressed in most tumor samples, and validated by immunohistochemical analysis [14]. This gene has been shown to be overexpressed in prostate cancer by several other groups. KLK3 (PSA) is the marker *par excellence *of prostate epithelial activity and cellular bulk, and detection of its serum protein levels is the best available marker for monitoring prostate cancer [47]. HER3 is a receptor for the paracrine growth factor neregulin-1, and a transmembrane protein that tethers the ligand to its dimerization partners, the receptor tyrosine kinases HER2 and HER4 [48], and known to play important roles in the development and progression of the malignant phenotype in breast cancer [49]. The abnormal expression and activity of HER2 has been studied extensively in the context of prostate cancer [50], being found overexpressed in advanced tumors, either metastatic or homone-independent, but infrequently in primary, organ-confined tumors. More controversial is the information available on the role of HER3, with reports of its overexpression in prostate cancer together with HER2, HER4, or both [51,52], but also of its overexpression only in metastatic tumors, in particular of a truncated form corresponding to the extracellular domains of HER3 [53]. Furthermore, several transcriptional profiling analyses have found overexpression of this gene in prostate cancer. IQGAP2 is a calmodulin-binding protein that participates in cell signalling and modulation of cytoskeletal dynamics [37], and its activity has been reported to be positively [54] and negatively associated with neoplastic phenotype. POR1/ARFAPTIN2, a Rac1-interacting protein [55], is a regulator of cytoskeletal dynamics that so far has not been associated with any particular type of neoplasia.

**Figure 5:**
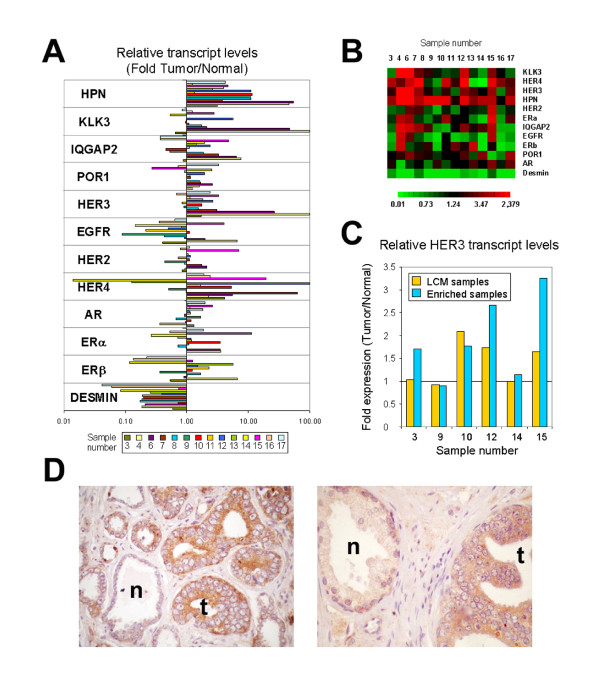
Validation of genes selected by FADA from the Welsh *et al. *dataset [14] as overexpressed in prostate cancer. **(A) **RT-PCR was applied to 14 paired prostate tumor – normal prostate samples to determine the expression levels of a selection of genes shown by FADA as significantly overrepresented in prostate cancer (HPN, KLK3, IQGAP2, POR1 and HER3), and additional genes relevant to this tumor (genes for the receptor tyrosine kinases EGFR, HER2, HER4, and genes for the steroid hormone receptors AR, ERα and ERβ). The expression values for each gene, previously normalized with respect to the S14r expression level in each sample, are shown as ratios of the normalized values in prostate cancer *vs*. values in the matching normal prostate tissue. Quantitation of desmin expression levels was used to assess the degree of contribution of stromal components in the samples analyzed. Values equal to or above 100-fold are shown as 100. **(B) **Heatmap representation of the same data (color scale as shown below). **(C) **Real-time PCR analysis for HER3 transcript levels of laser microdissected tumor and normal samples, compared with relative transcript levels in enriched (non-microdissected) tissues from the same cases. **(D) **Immunohistochemical analysis of HER3 on paraffin-embedded prostate tissue sections arranged in tissue microarrays (see Methods). Left, low magnification image (×100) of one case, with weak staining for HER3 in normal glands **(n)**, and a strong staining in tumor epithelial cells **(t)**. Right, higher magnification (×400) of a second case.

The results of semiquantitative real-time RT-PCR on our samples indicate that hepsin is significantly overexpressed in 14 out of 14 cases, IQGAP2 in 8 of 14, and HER3 in 10 of 14 cases (Figure 5). Other genes analyzed, such as KLK3 (PSA), HER2 or the steroid receptors androgen receptor, estrogen receptor α or estrogen receptor β are less frequently overexpressed in these tumors. Levels of desmin transcripts were determined as an index of the contribution of stromal cells, suggesting that the overexpression of the analyzed genes are detected in tumor samples even in the presence of substantial stromal contamination (Figure 5). Of particular interest is the observed upregulation of HER3 in prostate tumor tissues relative to normal tissues. The HER3/ErbB3 protein has impaired intrinsic kinase activity [56], and it appears to function in signal transduction by tethering the ligand to other members of the HER family of receptors, with preference for HER2/ErbB2 [57]. Increased levels of expression of HER3 are seen in many tumors that express HER2 [58], and it is widely assumed that the signalling and/or oncogenic functions reside in the corresponding heterodimer, rather than in either individual receptor [59,60]. Recent experimental evidence further highlights the importance of HER3 in conferring a malignant phenotype and a hormone-refractory state to prostate epithelial cells [61]. Thus, whenever HER3 is expressed it is reasonable to expect co-expression of at least one other member of the HER family. Therefore, we determined by real-time RT-PCR the relative expression in our prostate tissue samples of the genes for all four members of the HER family of receptor tyrosine kinases. Our results show that HER4 is expressed at increased levels in 10 of 14 prostate tumor samples (Fig. 5A, B), whereas HER2/ErbB2 and EGFR are overexpressed in 3 of the 14 samples analyzed. Seven samples simultaneously overexpressed HER3 and HER4, of which 2 overexpressed all four members of the HER family (Fig. 5A, B). None of the samples overexpressed the pairs HER3 and HER2, or HER3 and EGFR, without overexpressing at the same time one of the other members of the family (Fig. 5A, B).

As mentioned in the Methods section, both tumor and normal tissues were carefully chosen to have similar representation of epithelial compartment. However, to further ensure that the observed expression of HER3 was not due to a dilution effect of normal epithelial cells by stroma, we performed real-time PCR analysis of laser microdissected samples. For this, we selected four samples that had shown overexpression of HER3 in the enriched tumor samples described above, and two that had levels that did not differ significantly from non-tumor containing (normal) matched tissues. Of the four samples in which the enriched tumor tissue had shown increased levels of HER3 transcript, three microdissected samples overexpressed HER3 (Fig. 5C). In two of the microdissected samples, HER3 transcript levels were equal in normal and tumor microdissected epithelia, and this also corresponded to samples in which HER3 levels did not differ significantly between enriched tumor and normal prostate tissues (Fig. 5C). This analysis showed that overexpression of HER3 in prostate tumor tissues is not due to simple enrichment of epithelial cells in comparison with non-tumor tissues. To further confirm the cell type expressing HER3 in prostate tissues, immunohistochemical analysis with a monoclonal antibody to HER3 was performed on 16 prostate samples, arranged in duplicate 1-mm diameter cores in tissue microarrays, in which both tumor and normal glands were present. HER3 protein was found clearly overexpressed in tumor epithelia in 13 of the 16 cases (81.2%), showing juxtamembrane and finely granular cytoplasmic patterns (Fig. 5D). In all cases, normal epithelia showed weak reactivities for HER3 (Fig. 5D).

In summary, our transcriptome re-analysis, validated by real-time RT-PCR of non-microdissected and microdissected samples and by immunohistochemical analysis, significantly reinforces previous immunohistochemical studies that reported high levels of expression of HER3 and HER4 in primary prostate cancer [51,52].

## Conclusion

We have shown that the method presented here for the analysis of expression microarray data permits the classification of samples into meaningful categories and, simultaneously, to identify a subset of genes and their assignment to pathways most significantly contributing to the corresponding phenotypes, while allowing for a given gene to participate as significant in more than one cluster of samples. The analysis of the yeast dataset validates the approach. Our results are consistent with biochemical pathways known to be activated in the different stress conditions analyzed, and the clustering of samples reflects the underlying similarity of the biochemical responses. In the application to the prostate cancer dataset, we have found that two pathways, one modulated by androgen receptor and a second one by signals that originate from cell surface growth factor receptors, are prominently active in the organ-confined, non-metastatic prostate cancer samples analyzed. The latter pathway has been reported to be spuriously active in at least a subset of prostate tumors that have progressed to invasive and hormone-independent states [62]. Our results suggest that such altered activation may already be present in primary tumors. Although a prevailing model for prostate tumor progression is that acquisition of the capacity for metastatic and hormone independent growth proceeds through selection of rare populations of cells concealed among primary tumor cells, there is also evidence that a transcriptional program for metastasis may already be present in the bulk of primary tumors at the time of diagnosis [63,64]. Our analysis would be more consistent with the latter model.

Finally, we have unveiled and validated several markers highlighted by the analysis of the prostate cancer dataset. While several of these genes were identified in the original analysis of the data [14], others are revealed here, notably HER3, IQGAP2 and POR1, the biologically most relevant being HER3. With an external dataset, we have found that prostate cancer samples frequently co-overexpress HER3 and HER4, accompanied less frequently by increased expression of EGFR or HER2. Overexpression of HER2 and consequent increased signalling have been associated with advanced prostate cancer, development of hormone independent state and poor prognosis [65,66], but is infrequently observed in primary tumors [67,68]. On the other hand, our results suggest that, in primary prostate cancer, HER3, together or not with HER4, rather than receptor complexes involving HER2, could play important roles in the biology of these tumors.

## Materials and methods

### Datasets

The *S. cereviseae *dataset consists of transcriptional responses of the yeast *S. cerevisiae *to environmental stress [13]. It originally consists of spotted array measurements of 6152 genes in 173 experimental conditions that include temperature shocks, hyper and hypoosmotic shocks, exposure to various agents such as peroxide, menadione, diamide, dithiothreitol, amino acid starvation, nitrogen source depletion and progression into stationary phase. Log-ratios were preprocessed following several steps: first data from genes with missing values were filtered out, and their missing values estimated with LSimpute [69] using the *'Adaptive' *method. Next, ratios were computed from the log-ratios and quantile-normalized (experiment-wise) using the normalizeQuantile function from the R package [70], so that all experiments had the same average sample distribution. Finally, ratios were log transformed again.

The prostate cancer dataset chosen is described in [14]. It was originally obtained by hybridizations on Affymetrix U95A oligonuleotide arrays with probes for a total of 55 samples. Intensity values were preprocessed following several steps: first intensity data were thresholded, with intensities below 10 fixed at 10 and values above 16000 fixed at 16000. The thresholded values were log-transformed and then centered by the median of all experiments. Finally, genes were subjected to z-transformation (per gene basis).

### Determination of genotypically coherent groups of samples

Q-mode Factor Analysis (FA) [9] seeks to find an underlying orthogonal factor model of an original **X**-matrix *nxm *(where *n *are the number of samples and *m *the number of mRNA levels measured) of the form:

X = LF + E

**L **is the loadings matrix of size *nxk*, where *k *is the number of factors, and **F **the *scores matrix *of size *kxm*, while **E **is the *residual matrix*, which contains both the specific variance of the individual genes and the errors in the model (see Figure 1). We used the so-called *principal factor solution *to solve this factor model. Specifically, in a first step, and based on the correlation matrix **R **derived from **X**, communalities (i.e. the proportion of the variance explained by common factors) were computed from the multiple squared correlation coefficient between the ith variable and the rest. These communalities replaced the diagonal entries of the correlation matrix, which was subjected to diagonalization. New communalities were computed from the loadings at the chosen dimensionality, obtained by scaling the eigenvector matrix **(P)**, as follows:

**L = P Λ**^1/2^

The new communalities again replaced the diagonal entries, and the process was iterated until convergence. Finally, we proceeded to rotate the factor loadings by means of a *varimax *rotation [9]. The effect of this rotation is to maximally align each of the samples with one factor in order to simplify the factor model and make it more readily interpretable. Phyletic trees were derived by clustering samples in loadings space at the optimal dimensionality using average linkage [4,11]. When needed, bootstrap values were computed by selecting random subsets of 90% of the genes [71]. Distribution of trees and frequency of each branch in the original tree were recorded using CONSENSE, program included in the PHYLIP package [72].

### Selecting genes associated to each cluster

Once sample clusters are defined, these are used to identify groups of genes contributing heavily to the specific character of different groups. Each gene on the list is subjected to a Student's *t*-test that measures the differential expression of the gene in the cluster as compared with the rest of the samples. *t*-test scores were transformed to *q*-values, which include multiple testing correction. The *q*-value is similar to the well known *P*-value, except that it is a measure of significance in terms of the false discovery rate, rather than the false positive rate [73]. Genes with a *q*-value < 10^-4 ^were taken as differentially expressed for that particular cluster.

### Assigning pathways to gene clusters

The association between selected genes and biological functions was established by determining the hypergeometric distribution of genes on the annotation databases GO [12] or GenMapp [32]. With this distribution we computed the probability that at least *x *genes annotated within a given biological function according to GO (or GenMapp) in a cluster of size *n *(the total number of genes per cluster selected in the previous step) can be obtained by chance, given a population of *N *genes under consideration and given *A*, the total number of genes within *N *with that particular annotation. These *P*-values are obtained according to:



An aggregated score for each cluster from the significant *P*-values (i.e., those below 10^-2^) is computed as follows:

*s*_0 _= ∑-ln *p*(*x*; *N*, *A*, *n*)

The significance of this score is established by simulation. We randomly selected 100 samples of size *n *genes each (the number of genes per cluster selected according to the *q*-value) and computed a new *s*-score (*s*_*r*_) for each one. The *Z*-score is finally computed as:

*z *= (*s*_*o *_- <*s*_*r*_>)/*σ*_*r*_

*Z*-scores > 2.0 are taken as indicative of significant association between the samples in the cluster and the set of pathways uncovered.

We should emphasize that in spite of the apparent intricacy of the computational procedure, the computational complexity is similar to other biclustering methods, and operates within a highly constrained parameter space: in the factor analysis part of the program only the percentage of variance employed should be set, yielding a reduced number of dimensions or latent variables, usually below 5; the number of clusters is automatically determined in this space from the *c*-index, and has no free parameters, and the selection of genes relevant for each cluster only depends on the cutoff employed in the *q*-value.

### Real-time RT-PCR

We used RT-PCR with either TaqMan probes or by SYBRGreen incorporation to determine the expression levels of selected genes on samples unrelated to the original study by Welsh *et al. *In each instance, the tumor sample and its matching normal counterpart were obtained from the same case, upon removal by radical prostatectomy. Serial sections from all normal counterparts to the tumor tissues were stained and analyzed to confirm that normal prostate glands and epithelial cells were present in near-normal patterns, and that they contained less than 1% of cells or structures with carcinomatous appearance. In addition, samples were chosen such that the tumor and normal counterparts in each case had approximately equal representations of the epithelial compartment, as assessed microscopically. RNA was isolated from corresponding frozen serial sections, and controlled for quality on a 2100 BioAnalyzer instrument (Agilent, Palo Alto, CA). For each sample, 0.5 μg of total RNA was reverse transcribed by priming with random hexamers at 42°C for 50 minutes, followed by treatment with RNase at 37°C for 20 min. The resulting cDNAs were used as templates in PCR reactions with gene-specific primers. Real-time PCR was performed on ABI PRISM 7700 (Applied Biosystems, Foster City, CA) or DNA Engine Opticon (MJ Research, Waltham, MA) instruments. TaqMan probes and their corresponding primer sets were obtained from Applied Biosystems. Thermal cycler conditions were 95°C for 10 min and 40 cycles of 95°C for 15 sec and 60°C for 1 minute for TaqMan assays. In the case of SYBRGreen reactions, the conditions were 95°C for 15 min, and 40 cycles of 95°C for 15 sec, 55°C for 30 sec and 72°C for 30 sec. All determinations were performed in triplicate and in at least two independent experiments. Since the relative amplification efficiencies of target and reference samples were found to be approximately equal, the ΔΔCt method was applied to estimate relative transcript levels. Levels of ribosomal S14r amplification were used as an endogenous reference to normalize each sample value of Ct (threshold cycle) and normal tissues were used as calibrators for their tumoral counterparts in each case. The final results, expressed as *n*-fold differences in target gene expression were calculated as follows:

n_TARGET _= 2^-[(Ct target - Ct reference)TUMORAL - (Ct target - Ct reference)NORMAL]^

### Laser capture microdissection

Prostate tissues were obtained by punch sections of radical prostatectomies and snap-frozen in isopentane at -50°C embedded in OCT-containing cryomolds. 8 μM cryosections were mounted onto plastic membrane-covered glass slides (PALM Mikrolaser Technology, Bernried, Germany), fixed for 3 minutes in 70% ethanol, stained with Mayer's hematoxilin, dehydrated, air-dried for 10 minutes and stored at -80°C until used. Laser catapulting microdissection was performed with a PALM MicroBeam Systems instrument. 2 to 5 × 10^4 ^normal or carcinomatous epithelial cells were collected and estimated to be >99% homogeneous by microscopic visualization.

Total RNA from microdissected samples was isolated using the PicoPure RNA Kit (Arcturus Engineering, Santa Clara, CA), with an additional DNase I digestion step (Qiagen, Valencia, CA).

### Immunohistochemistry

Sixteen paraffin embedded prostate samples were evaluated for HER3 expression by immunohistochemistry on a tissue microarray. The cases were represented in duplicated 1-mm diameter cores and always included normal prostatic glands adjacent to neoplastic foci in at least one of the cores. Three μM sections of the microarray were deparaffinized, rehydrated and subjected to antigen retrieval in a pressure cooker with citrate buffer at pH 6.0 for 5 min. Slides were cooled for 15 min, washed in water and incubated overnight at 4°C with anti-HER3 mouse monoclonal antibody (Upstate Biotechnology, Lake Placid, New York). Endogenous peroxidase was quenched and slides were incubated for 30 minutes with secondary antibody (Envision, DAKO, Gostrup, Denmark). Reactions were detected after development with diaminobencidine and H_2_O_2 _for 3 min. Slides were counterstained with Harri's hematoxilin, dehydrated and mounted. As a negative control, the primary antibody was substituted for isotype-matched mouse IgG.

### Access to the program

The complete procedure has been coded in a Fortran-77 program, called FADA. Remote access to the program has been enabled by setting up a web-server where the program can be executed [74].

## Authors' contributions

JJL and DA implemented the software and carried out the analysis; MS and RB performed the RT-PCR and Inmunohistochemistry experiments; PLF obtained the prostate samples and carried out the laser capture microdissections; TMT and ARO coordinated the work and wrote the manuscript.

## Supplementary Material

Additional File 1 List of genes significantly associated to each cluster in the yeast dataset (*q*-value < 10^-3^). Click here for file

Additional File 2 List of genes significantly associated to each cluster in the prostate cancer dataset (*q*-value < 10^-3^). Click here for file
