# Supplementary material for: Dual activation of pathways regulated by steroid receptors and peptide growth factors in primary prostate cancer revealed by Factor Analysis of microarray data
Source: BMC Genomics. 2005 Aug 17;6:109. doi: 10.1186/1471-2164-6-109 (PMC1239914; doi:10.1186/1471-2164-6-109)
Supplement: Additional File 1 — List of genes significantly associated to each cluster in the yeast dataset (q-value < 10-3). [file 1471-2164-6-109-S1.doc]

**Table 1.** Genes most relevant according to FADA for the yeast dataset. For each gene, the *t-*test q-value, the t-statistic, the gene name, and gene description are shown.

| **Gene** | **Q-value** | **T-statistic** | **Description** |
| --- | --- | --- | --- |
| Cluster 1 |  |  |  |
| SSA4 | 1.48E-23 | 12.855 | CYTOSOLIC HSP70 |
| SSA3 | 1.64E-16 | 10.262 | CYTOSOLIC HSP70 |
| HSP78 | 5.01E-16 | 10.023 | MITOCHONDRIAL |
| SSE2 | 6.78E-16 | 9.919 | HSP70 FAMILY |
| TOR1 | 6.78E-16 | 9.896 | PHOSPHATIDYLINOSITOL 3-KINASE |
| MTC2 | 1.14E-15 | 9.784 | UNKNOWN |
| --- | 1.57E-15 | 9.71 | UNKNOWN; SIMILAR TO SUR7P |
| GRE3 | 1.84E-14 | 9.298 | INDUCED BY OSMOTIC STRESS |
| HSP104 | 2.74E-14 | 9.215 | HEAT SHOCK PROTEIN |
| ALD3 | 8.24E-14 | 9.005 | ALDEHYDE DEHYDROGENASE |
| HSP82 | 1.36E-13 | 8.909 | HSP90 HOMOLOG |
| STI1 | 4.84E-13 | 8.677 | COMPONENT OF HSP70-HSP90 COMPLEXES |
| MDJ1 | 5.06E-13 | 8.648 | CHAPERONE; DNAJ HOMOLOG |
| TPS1 | 7.80E-13 | 8.567 | TREHALOSE-6-PHOSPHATE SYNTHAS |
| CPR6 | 1.02E-12 | 8.504 | PEPTIDYL-PROLYL CUS-TRANS ISOMERASE |
| ECM12 | 1.02E-12 | 8.504 | UNKNOWN |
| PTK2 | 1.91E-12 | 8.392 | SER/THR PROTEIN KINASE |
| MYO3 | 8.19E-12 | 8.133 | MYOSIN, CLASS I |
| ARO9 | 1.43E-11 | 8.004 | AROMATIC AMINO ACID AMINOTRANSFERASE II |
| SIS1 | 1.81E-11 | 7.958 | HEAT SHOCK PROTEIN, HOMOLOG OF E. COLI D |
| UBI4 | 2.46E-11 | 7.889 | UBIQUITIN |
| BTN2 | 4.08E-11 | 7.787 | UNKNOWN |
| HSP42 | 4.24E-11 | 7.775 | HEAT SHOCK PROTEIN, SIMILAR TO HSP26 |
| SOL4 | 8.62E-11 | 7.648 | UNKNOWN; SIMILAR TO SOL3P |
| FAA1 | 9.49E-11 | 7.625 | LONG CHAIN FATTY ACYL:COA SYNTHETASE |
| ALD2 | 1.21E-10 | 7.575 | ALDEHYDE DEHYDROGENASE |
| NTH1 | 1.25E-10 | 7.565 | ALPHA, ALPHA-TREHALASE |
| IKS1 | 1.39E-10 | 7.543 | PROTEIN KINASE |
| ARC35 | 2.29E-10 | 7.443 | CORTICAL ACTIN PATCH INTEGRITY |
| ATM1 | 2.42E-10 | -7.427 | REGULATOR OF MIT. IRON TRANSPORTER |
| UGP1 | 2.42E-10 | 7.426 | UGP1, UDP-GLUCOSE PYROPHOSPHORYLASE |
| HSC82 | 2.53E-10 | 7.404 | CHAPERONIN |
| SPI1 | 2.56E-10 | 7.398 | UNKNOWN; SIMILAR TO SED1P; INDUCED IN ST |
| PGM2 | 3.75E-10 | 7.321 | PHOSPHOGLUCOMUTASE |
| BAG7 | 4.99E-10 | 7.265 | GTPASE ACTIVATING PROTEIN |
| ENT3 | 6.94E-10 | 7.196 | UNKNOWN; EPSIN HOMOLOG |
| YSC84 | 9.63E-10 | 7.132 | UNKNOWN; SIMILAR TO HYPOTHETICAL PROTEIN |
| NUC1 | 1.19E-09 | -7.088 | ENDONUCLEASE |
| KSP1 | 1.50E-09 | 7.039 | PROTEIN KINASE |
| YMC1 | 1.66E-09 | -7.015 | (PUTATIVE) MITOCHONDRIAL CARRIER |
| SOL1 | 1.70E-09 | 7.008 | UNKNOWN |
| GPH1 | 2.60E-09 | 6.923 | GLYCOGEN PHOSPHORYLASE |
| GCN4 | 3.00E-09 | -6.893 | TRANSCRIPTION FACTOR |
| RNY1 | 4.14E-09 | 6.827 | RIBONUCLEASE, T2 FAMILY |
| MSB3 | 4.37E-09 | 6.814 | UNKNOWN; SUPPRESSES BUD EMERGENCE MUTANT |
| SSA1 | 5.49E-09 | 6.761 | CYTOSOLIC HSP70 |
| TRM1 | 5.99E-09 | -6.74 | TRNA METHYLTRANSFERASE |
| TSL1 | 5.99E-09 | 6.739 | TREHALOSE-6-PHOSPHATE SYNTHASE/PHOSPHATA |
| POP8 | 5.99E-09 | -6.737 | RNASE P AND RNASE MRP SUBUNIT |
| HCH1 | 6.20E-09 | 6.728 | UNKNOWN; OVEREXPRESSION SUPPRESSES HSP82 |
| TPS3 | 6.45E-09 | 6.717 | ALPHA,ALPHA-TREHALOSE-PHOSPHATE SYNTHASE |
| HSP60 | 6.45E-09 | 6.715 | MITOCHONDRIAL CHAPERONIN |
| HPT1 | 6.62E-09 | -6.708 | HYPOXANTHINE GUANINE PHOSPHORIBOSYL TRAN |
| FAA4 | 9.80E-09 | -6.626 | LONG-CHAIN-FATTY-ACID--COA LIGASE |
| MRF1' | 1.16E-08 | 6.587 | ARS-BINDING PROTEIN |
| HOR2 | 1.56E-08 | 6.525 | DL-GLYCEROL-3-PHOSPHATASE |
| PHR1 | 1.56E-08 | 6.524 | DEOXYRIBODIPYRIMIDINE PHOTOLYASE |
| APM2 | 1.65E-08 | 6.511 | AP-2 COMPLEX SUBUNIT |
| SRA1 | 1.72E-08 | 6.5 | CAMP DEPENDENT PROTEIN KINASE, REGULATOR |
| RMT2 | 2.20E-08 | -6.453 | ARGININE METHYLTRANSFERASE |
| GLK1 | 2.24E-08 | 6.448 | GLUCOKINASE |
| DBP9 | 2.28E-08 | -6.443 | PUTATIVE RNA HELICASE |
| AIP1 | 2.72E-08 | 6.403 | ACTIN CORTICAL PATCH COMPONENT |
| LTV1 | 3.15E-08 | -6.373 | UNKNOWN; REQUIRED FOR VIABILITY AT LOW T |
| URA5 | 3.50E-08 | -6.351 | OROTATE PHOSPHORIBOSYLTRANSFERASE |
| GLO1 | 3.53E-08 | 6.347 | GLYOXALASE I |
| SGA1 | 3.71E-08 | 6.334 | GLUCAN 1,4-ALPHA-GLUCOSIDASE |
| FRE1 | 3.80E-08 | -6.328 | FERRIC (AND CUPRIC) REDUCTASE |
| PPH3 | 3.96E-08 | -6.315 | PROTEIN PHOSPHATASE 2A |
| NMD3 | 4.16E-08 | -6.303 | NAM7P/UPF1P-INTERACTING PROTEIN |
| SMP3 | 4.42E-08 | -6.287 | INTEGRAL MEMBRANE, PROTEIN KINASE C PATH |
| CAR2 | 4.77E-08 | 6.269 | ORNITHINE AMINOTRANSFERASE |
| KKQ8 | 5.18E-08 | 6.252 | PROTEIN KINASE |
| TIN1 | 5.25E-08 | 6.248 | TOR INHIBITOR (UNPUBLISHED) |
| TPS2 | 5.39E-08 | 6.241 | TREHALOSE-6-PHOSPHATE PHOSPHATASE |
| SAS5 | 5.39E-08 | -6.24 | UNKNOWN |
| TKL2 | 5.51E-08 | 6.235 | TRANSKETOLASE |
| YVH1 | 5.68E-08 | -6.227 | PROTEIN PHOSPHATASE |
| ULA1 | 5.90E-08 | 6.214 | RUB1P ACTIVATING PROTEIN |
| CWP1 | 6.18E-08 | 6.204 | BETA-1,6-GLUCAN ACCEPTOR |
| ILV1 | 6.29E-08 | -6.196 | THREONINE DEAMINASE |
| DSS4 | 6.29E-08 | -6.194 | GDP/GTP EXCHANGE FACTOR FOR SEC4P |
| SSE1 | 6.32E-08 | 6.192 | HSP70 FAMILY |
| FRE6 | 6.34E-08 | -6.19 | UNKNOWN; SIMILAR TO FERRIC REDUCTASE FRE |
| ECM33 | 6.37E-08 | -6.188 | UNKNOWN |
| XKS1 | 6.59E-08 | 6.18 | XYLULOKINASE |
| MDL1 | 6.86E-08 | -6.171 | ATP-BINDING CASSETTE (ABC) FAMILY |
| SPT15 | 6.93E-08 | -6.168 | TFIID AND TFIIIB SUBUNIT |
| TAH18 | 7.04E-08 | -6.162 | UNKNOWN; SIMILAR TO NAPDH-CYTOCHROME P45 |
| SSA2 | 7.08E-08 | 6.16 | CYTOSOLIC HSP70 |
| YHM1 | 7.96E-08 | -6.135 | MITOCHONDRIAL CARRIER FAMILY |
| POL30 | 8.27E-08 | -6.125 | DNA POLYMERASE PROCESSIVITY FACTOR |
| TFS1 | 8.61E-08 | 6.116 | SUPPRESSES CDC25 MUTATIONS |
| UBP2 | 9.66E-08 | 6.088 | UBIQUITIN-SPECIFIC PROTEASE |
| RAD27 | 9.85E-08 | -6.079 | SSDNA ENDONUCLEASE |
| HSP10 | 9.85E-08 | 6.079 | MITOCHONDRIAL CHAPERONIN |
| IMP4 | 1.07E-07 | -6.059 | U3 SNORNP PROTEIN |
| RLP7 | 1.07E-07 | -6.057 | RIBOSOMAL PROTEIN L7 (PUTATIVE) |
| ALD4 | 1.29E-07 | 6.012 | MITOCHONDRIAL ALDEHYDE DEHYDROGENASE |
| CTT1 | 1.36E-07 | 6.001 | CATALASE T |
| Cluster 2 |  |  |  |
| FLR1 | 8.50E-21 | 11.877 | TRANSPORTER, MAJOR FACILITATOR SUPERFAMI |
| --- | 8.50E-21 | 11.823 | PUTATIVE ARYL-ALCOHOL REDUCTASE |
| AAD6 | 6.06E-20 | 11.461 | HYPOTHETICAL ARYL-ALCOHOL DEHYDROGENASE |
| AAD4 | 1.35E-18 | 10.941 | HYPOTHETICAL ARYL-ALCOHOL DEHYDROGENASE |
| GTT2 | 2.37E-17 | 10.462 | GLUTATHIONE TRANSFERASE |
| RIB3 | 1.63E-16 | 10.109 | 3,4-DIHYDROXY-2-BUTANONE 4-PHOSPHATE SYN |
| AAD15 | 2.25E-16 | 10.038 | HYPOTHETICAL ARYL-ALCOHOL DEHYDROGENASE |
| AAD3 | 4.80E-15 | 9.535 | HYPOTHETICAL ARYL-ALCOHOL DEHYDROGENASE |
| AAD14 | 4.74E-14 | 9.137 | HYPOTHETICAL ARYL-ALCOHOL DEHYDROGENASE |
| NBP35 | 6.62E-14 | 9.069 | UNKNOWN; ESSENTIAL; SIMILAR TO BACTERIAL |
| ZWF1 | 8.53E-12 | 8.235 | GLUCOSE-6-PHOSPHATE DEHYDROGENASE |
| DDI1 | 9.77E-12 | 8.202 | UNKNOWN; INDUCED BY DNA DAMAGE |
| PUP1 | 1.34E-11 | 8.139 | 20S PROTEASOME SUBUNIT (BETA2) |
| UFD1 | 2.98E-11 | 7.995 | UNKNOWN; UBIQUITIN FUSION DEGRADATION |
| AAD10 | 1.41E-10 | 7.694 | HYPOTHETICAL ARYL-ALCOHOL DEHYDROGENASE |
| TRR1 | 2.21E-10 | 7.602 | THIOREDOXIN REDUCTASE |
| MAG1 | 2.29E-10 | 7.582 | 3-METHYLADENINE DNA GLYCOSYLASE |
| KSS1 | 2.13E-09 | 7.177 | PROTEIN KINASE |
| GLR1 | 5.38E-09 | 6.999 | GLUTATHIONE REDUCTASE |
| MRS4 | 6.18E-09 | 6.962 | MITOCHONDRIAL CARRIER |
| CAF17 | 7.58E-09 | 6.92 | COMPONENT OF CCR4 TRANSCRIPTIONAL COMPLE |
| PRE1 | 7.87E-09 | 6.903 | 20S PROTEASOME SUBUNIT C11(BETA4) |
| ISA2 | 1.99E-08 | 6.713 | UNKNOWN; IRON SULFUR ASSEMBLY -- ISCA/NI |
| CDC20 | 2.24E-08 | -6.687 | ACTIVATOR OF THE ANAPHASE PROMOTING COMP |
| PRE5 | 2.59E-08 | 6.655 | 20S PROTEASOME SUBUNIT(ALPHA6) |
| PRE2 | 2.83E-08 | 6.629 | 20S PROTEASOME SUBUNIT (BETA5) |
| PRE10 | 4.90E-08 | 6.519 | 20S PROTEASOME SUBUNIT C1 (ALPHA7) |
| RPT5 | 5.34E-08 | 6.499 | 26S PROTEASOME REGULATORY SUBUNIT |
| UMP1 | 7.82E-08 | 6.416 | 20S PROTEASOME MATURATION FACTOR |
| TTR1 | 7.82E-08 | 6.415 | GLUTAREDOXIN |
| GRE2 | 9.22E-08 | 6.38 | UNKNOWN; INDUCED BY OSMOTIC STRESS |
| RFA2 | 1.19E-07 | 6.324 | REPLICATION FACTOR A 36 KD SUBUNIT |
| RPT6 | 1.19E-07 | 6.318 | 26S PROTEASOME REGULATORY SUBUNIT |
| BET4 | 1.28E-07 | 6.299 | GERANYLGERANYL TRANSFERASE SUBUNIT |
| GSH2 | 1.40E-07 | 6.279 | GLUTATHIONE SYNTHETASE |
| TRX2 | 1.64E-07 | 6.239 | THIOREDOXIN II |
| ECM4 | 1.71E-07 | 6.227 | UNKNOWN |
| ROK1 | 1.74E-07 | -6.221 | RNA HELICASE |
| SOL3 | 1.94E-07 | 6.19 | UNKNOWN |
| SFA1 | 2.32E-07 | 6.153 | LONG-CHAIN ALCOHOL DEHYDROGENASE |
| SOR1 | 2.45E-07 | 6.136 | SORBITOL DEHYDROGENASE |
| PRE3 | 3.65E-07 | 6.055 | 20S PROTEASOME SUBUNIT (BETA1) |
| UBC13 | 3.65E-07 | 6.053 | E2 UB.-CONJUGATING ENZYME |
| SLF1 | 5.15E-07 | 5.981 | CUS BIOMINERALIZATION |
| PHB2 | 5.20E-07 | 5.975 | PROHIBITIN HOMOLOG |
| RPN3 | 5.48E-07 | 5.962 | 26S PROTEASOME REGULATORY SUBUNIT |
| MAS1 | 6.11E-07 | 5.935 | MITOCHONDRIAL PROCESSING PROTEASE SUBUNI |
| ZRT2 | 6.18E-07 | -5.931 | ZINC TRANSPORTER |
| ARP2 | 6.28E-07 | 5.924 | ACTIN-RELATED PROTEIN |
| SGD1 | 9.15E-07 | -5.844 | HIGH OSMOLARITY PATHWAY |
| RPN12 | 9.83E-07 | 5.828 | 26S PROTEASOME REGULATORY SUBUNIT |
| GRX5 | 1.20E-06 | 5.781 | UNKNOWN; SIMILAR TO LEGIONELLA PNEUMOPHI |
| PRD1 | 1.20E-06 | 5.777 | PROTEINASE YSCD |
| PRE8 | 1.31E-06 | 5.758 | 20S PROTEASOME SUBUNIT Y7 (ALPHA2 |
| IFH1 | 1.37E-06 | -5.745 | UNKNOWN |
| LAP4 | 1.40E-06 | 5.738 | VACUOLAR AMINOPEPTIDASE YSC1 |
| SHP1 | 1.41E-06 | 5.734 | (PUTATIVE) GLC7P REGULATORY SUBUNIT |
| RPT4 | 2.06E-06 | 5.648 | 26S PROTEASOME REGULATORY SUBUNIT |
| GCR2 | 2.11E-06 | -5.64 | TRANSCRIPTIONAL ACTIVATOR |
| RSA1 | 2.32E-06 | -5.619 | UNKNOWN |
| ECM16 | 2.38E-06 | -5.612 | UNKNOWN |
| CYT2 | 2.38E-06 | 5.61 | CYTOCHROME C1 HEME LYASE |
| TRK1 | 2.73E-06 | -5.579 | POTASSIUM PERMEASE |
| ISU2 | 2.94E-06 | 5.56 | UNKNOWN; SIMILAR TO IRON-SULFUR CLUSTER |
| RPC82 | 3.55E-06 | -5.515 | RNA POLYMERASE III 82 KD SUBUNIT |
| PUP3 | 3.68E-06 | 5.505 | 20S PROTEASOME SUBUNIT (BETA3 |
| ADH4 | 3.70E-06 | -5.502 | ALCOHOL DEHYDROGENASE IV |
| RAD23 | 4.39E-06 | 5.463 | UBIQUITIN-LIKE PROTEIN |
| GAL83 | 4.45E-06 | 5.458 | COMPONENT OF SNF1 COMPLEX |
| YRR1 | 4.54E-06 | 5.446 | TRANSCRIPTIONAL ACTIVATOR OF SNQ2 |
| RIB5 | 4.54E-06 | 5.445 | RIBOFLAVIN SYNTHASE, ALPHA CHAIN |
| RTT104 | 4.67E-06 | -5.436 | UNKNOWN; SIMILAR TO PIF1P AND OTHER HELI |
| YJU3 | 4.93E-06 | 5.423 | UNKNOWN; SIMILAR TO E.COLI HYPOTHETICAL |
| OYE2 | 5.19E-06 | 5.402 | NAPDH DEHYDROGENASE (OLD YELLOW ENZYME) |
| NTG1 | 5.34E-06 | 5.393 | DNA GLYCOSYLASE |
| MRPL10 | 5.76E-06 | 5.375 | RIBOSOMAL PROTEIN, MITOCHONDRIAL L10 |
| LEU3 | 6.33E-06 | -5.351 | TRANSCRIPTION FACTOR |
| RPT2 | 6.33E-06 | 5.351 | 26S PROTEASOME SUBUNIT |
| PRE4 | 8.37E-06 | 5.289 | PROTEASOME SUBUNIT, B TYPE |
| LYS7 | 9.00E-06 | 5.272 | COPPER CHAPERONE FOR SUPEROXIDE DISMUTAS |
| IMG2 | 1.11E-05 | 5.222 | UNKNOWN |
| RAD52 | 1.21E-05 | 5.2 | RAD51P COFACTOR |
| YTA12 | 1.24E-05 | 5.195 | MITOCHONDRIAL CHAPERONIN |
| SNU66 | 1.26E-05 | -5.189 | U4/U6.U5 SNRNP PROTEIN |
| PIM1 | 1.30E-05 | 5.181 | MITOCHONDRIAL ATP-DEPENDENT PROTEASE |
| CAK1 | 1.33E-05 | 5.175 | PROTEIN KINASE |
| MAE1 | 1.41E-05 | -5.157 | MITOCHONDRIAL MALIC ENZYME |
| SBA1 | 1.46E-05 | 5.145 | HSP90 ASSOCIATED CO-CHAPERONE |
| TRF5 | 1.61E-05 | -5.12 | RELATED TO DNA TOPOISOMERASE I |
| TOF2 | 1.66E-05 | -5.111 | INTERACTS WITH DNA |
| HSP42 | 1.69E-05 | 5.105 | HEAT SHOCK PROTEIN, SIMILAR TO HSP26 |
| CDC36 | 1.71E-05 | 5.101 | GENERAL NEGATIVE REGULATOR |
| ECM22 | 1.74E-05 | -5.096 | UNKNOWN; SIMILAR TO TRANSCRIPTION FACTOR |
| TSA1 | 1.86E-05 | 5.075 | THIOL-SPECIFIC |
| YTH1 | 1.88E-05 | 5.071 | CLEAVAGE/POLYADENYLATION SPECIFICITY FAC |
| FHL1 | 2.04E-05 | -5.046 | TRANSCRIPTIONAL ACTIVATOR |
| ECM38 | 2.07E-05 | 5.041 | GAMMA-GLUTAMYLTRANSFERASE |
| SCL1 | 2.19E-05 | 5.025 | 20S PROTEASOME SUBUNIT YC7ALPHA/Y8 |
| GSH1 | 2.25E-05 | 5.016 | GAMMA-GLUTAMYLCYSTEINE SYNTHETASE |
| SLK19 | 2.25E-05 | -5.015 | UNKNOWN; SYNTHETIC LETHAL WITH KAR3 |
| Cluster 3 |  |  |  |
| YIP3 | 2.78E-21 | 11.955 | UNKNOWN; INTERACTS WITH YPT PROTEIN(S) |
| UBS1 | 4.47E-17 | 10.359 | REGULATES CDC34P (UBIQUITIN-CONJUGATING |
| YPS4 | 2.29E-16 | 10.059 | GPI-ANCHORED ASPARTIC PROTEASE |
| SGE1 | 2.00E-15 | 9.699 | TRANSPORTER, MAJOR FACILITATOR SUPERFAMI |
| ERD2 | 1.31E-14 | 9.33 | HDEL RECEPTOR |
| PAU1 | 4.64E-13 | 8.703 | UNKNOWN; SIMILAR TO MEMBERS OF THE SRP1P |
| ERO1 | 3.74E-12 | 8.35 | PROTEIN DISULFIDE BOND FORMATION IN THE |
| YET1 | 1.06E-11 | 8.154 | ER 25 KDA TRANSMEMBRANE PROTEIN |
| EMP47 | 1.73E-11 | 8.064 | UNKNOWN; ER/GOLGI MEMBRANE PROTEIN |
| LHS1 | 3.45E-11 | 7.934 | CHAPERONE; ER PROTEIN TRANSLOCATION |
| OST2 | 1.41E-10 | 7.679 | OLIGOSACCHARYLTRANSFERASE COMPLEX SUBUNI |
| VPH2 | 1.74E-10 | 7.631 | VACUOLAR H+-ATPASE ASSEMBLY PROTEIN |
| FUS1 | 4.78E-10 | 7.434 | SH3 DOMAIN PROTEIN |
| SPC3 | 1.49E-09 | 7.219 | SIGNAL PEPTIDASE SUBUNIT |
| GFA1 | 1.62E-09 | 7.199 | CHITIN BIOSYNTHESIS |
| KTR1 | 2.89E-09 | 7.078 | MANNOSYLTRANSFERASE |
| PRP40 | 2.95E-09 | 7.067 | U1 SNRNP PROTEIN |
| JEM1 | 2.95E-09 | 7.062 | DNAJ-LIKE PROTEIN |
| SPC2 | 3.60E-09 | 7.022 | SIGNAL PEPTIDASE SUBUNIT |
| PTP2 | 6.01E-09 | 6.926 | PROTEIN TYROSINE PHOSPHATASE |
| DFG10 | 6.81E-09 | 6.899 | UNKNOWN |
| RHO4 | 1.50E-08 | -6.741 | GTP-BINDING PROTEIN, RHO FAMILY |
| CRH1 | 1.50E-08 | 6.74 | UNKNOWN; CELL WALL PROTEIN |
| CHS1 | 1.63E-08 | 6.721 | CHITIN SYNTHASE |
| PAU6 | 2.37E-08 | 6.642 | SERIPAUPERIN FAMILY |
| MPD1 | 4.34E-08 | 6.526 | RELATED TO PROTEIN DISULFIDE ISOMERASES |
| PAU5 | 5.25E-08 | 6.487 | UNKNOWN; SIMILAR TO MEMBERS OF THE SRP1P |
| PAU4 | 7.63E-08 | 6.403 | UNKNOWN; SERIPAUPERIN FAMILY |
| SOH1 | 9.75E-08 | 6.354 | UNKNOWN; SIMILAR TO RNA POLYMERASES |
| HNT1 | 1.41E-07 | 6.278 | UNKNOWN; SIMILAR TO PROTEIN KINASE C INH |
| PDI1 | 4.01E-07 | 6.064 | PROTEIN DISULFIDE ISOMERASE |
| HAC1 | 4.41E-07 | 6.043 | TRANSCRIPTION FACTOR |
| RHO1 | 6.57E-07 | 5.957 | GTP-BINDING PROTEIN, RHO FAMILY |
| KAR2 | 7.03E-07 | 5.94 | BIP HOMOLOG; ER PROTEIN TRANSLOCATION |
| EUG1 | 9.53E-07 | 5.877 | PROTEIN DISULFIDE ISOMERASE |
| MRP2 | 1.10E-06 | 5.841 | RIBOSOMAL PROTEIN, MITOCHONDRIAL S14 |
| SCW10 | 1.45E-06 | 5.77 | GLUCANASE (PUTATIVE) |
| CHS5 | 2.27E-06 | 5.677 | UNKNOWN |
| NYV1 | 3.12E-06 | 5.603 | VACUOLAR V-SNARE |
| MSP1 | 3.88E-06 | 5.557 | AAA-ATPASE |
| KTR2 | 4.50E-06 | 5.522 | PUTATIVE MANNOSYLTRANSFERASE; TYPE 2 MEM |
| RET3 | 5.27E-06 | 5.487 | VESICLE COAT COMPONENT |
| PMT6 | 5.71E-06 | 5.467 | PUTATIVE O-MANNOSYLTRANSFERASE |
| PRY2 | 5.86E-06 | 5.459 | UNKNOWN; SIMILAR TO PLANT PR-PATHOGEN RE |
| COS1 | 6.78E-06 | 5.424 | UNKNOWN; SIMILAR TO SUBTELOMERICALLY-ENC |
| SRL3 | 7.02E-06 | 5.415 | UNKNOWN; SUPPRESSOR OF RAD53 LETHALITY |
| TLG1 | 7.45E-06 | 5.4 | LATE GOLGI T-SNARE |
| COS6 | 7.64E-06 | 5.391 | UNKNOWN; SIMILAR TO OTHER SUBTELOMERICAL |
| FKB2 | 7.64E-06 | 5.385 | PEPTIDYL-PROLYL CIS-TRANS ISOMERASE |
| MET12 | 7.64E-06 | -5.384 | METHYLENETETRAHYDROFOLATE REDUCTASE |
| MNR2 | 8.39E-06 | 5.361 | UNKNOWN |
| CYP5 | 8.65E-06 | 5.353 | PEPTIDYL-PROLYL CIS-TRANS ISOMERASE |
| MNN5 | 9.32E-06 | -5.335 | ALPHA-1,2-MANNOSYLTRANSFERASE (PUTATIVE) |
| SML1 | 9.94E-06 | 5.319 | REGULATOR OF RIBONUCLEOTIDE REDUCTASE (P |
| BSD2 | 1.33E-05 | 5.249 | CU(2+) TRANSPORTER |
| ERG2 | 1.52E-05 | 5.218 | C-8 STEROL ISOMERASE |
| MID2 | 1.57E-05 | 5.21 | UNKNOWN; MUTANT IS SENSITIVE TO MATIN PH |
| ACB1 | 1.75E-05 | 5.183 | ACYL-COA ESTER TRANSPORTER |
| COS3 | 1.77E-05 | 5.179 | UNKNOWN; SIMILAR TO SUBTELOMERICALLY-ENC |
| YPS1 | 2.19E-05 | 5.131 | GPI-ANCHORED ASPARTIC PROTEASE |
| ADK1 | 2.21E-05 | 5.127 | CYTOSOLIC ADENYLATE KINASE |
| CSE2 | 2.37E-05 | 5.109 | KINETOCHORE PROTEIN (PUTATIVE) |
| BMH1 | 2.37E-05 | 5.108 | UNKNOWN; SIMILAR TO MAMMALIAN 14-3-3 PRO |
| SEC61 | 2.81E-05 | 5.067 | ER PROTEIN TRANSLOCATION COMPLEX SUBUNIT |
| SSP120 | 2.93E-05 | 5.053 | UNKNOWN |
| TSA1 | 3.46E-05 | 5.012 | THIOL-SPECIFIC |
| NUP120 | 3.69E-05 | 4.995 | NUCLEAR PORE PROTEIN |
| DOG2 | 3.71E-05 | 4.993 | 2-DEOXYGLUCOSE-6-PHOSPHATE PHOSPHATASE |
| PRP42 | 3.78E-05 | 4.985 | U1 SNRNP PROTEIN |
| BFR1 | 3.95E-05 | 4.972 | UNKNOWN |
| ARC19 | 4.25E-05 | 4.951 | CORTICAL ACTIN PATCH INTEGRITY |
| COS4 | 4.27E-05 | 4.948 | UNKNOWN; SIMILAR TO SUBTELOMERICALLY-ENC |
| CAN1 | 4.39E-05 | -4.938 | BASIC AMINO ACID PERMEASE |
| ASP3-3 | 4.39E-05 | 4.939 | L-ASPARAGINASE II |
| UBP7 | 4.65E-05 | 4.925 | UBIQUITIN-SPECIFIC PROTEASE |
| LSM7 | 4.82E-05 | 4.915 | SIMILAR TO SNRNA-ASSOCIATED PROTEIN |
| NRG2 | 4.83E-05 | 4.912 | UNKNOWN |
| SCJ1 | 4.83E-05 | 4.912 | UNKNOWN; SIMILAR TO E. COLI DNAJ |
| SRB6 | 5.14E-05 | 4.896 | RNA POLYMERASE MEDIATOR SUBUNIT |
| ASP3-4 | 6.67E-05 | 4.831 | L-ASPARAGINASE II |
| SRL1 | 7.08E-05 | 4.815 | UNKNOWN; SIMILAR TO SVS1P; SUPPRESSOR OF |
| MRP8 | 7.12E-05 | 4.811 | RIBOSOMAL PROTEIN, MITOCHONDRIAL SMALL S |
| SAP4 | 7.92E-05 | 4.786 | SIT4P-ASSOCIATED PROTEIN |
| YPK1 | 8.51E-05 | 4.767 | PROTEIN KINASE |
| SFB2 | 1.01E-04 | 4.725 | UNKNOWN; SIMILAR TO SEC24P; BINDS SED3P |
| MOB1 | 1.02E-04 | 4.722 | UNKNOWN; BINDS MPS1P AND DBF2P |
| HEM15 | 1.10E-04 | 4.7 | FERROCHELATASE (PROTOHEME FERROLYASE) |
| ARF2 | 1.11E-04 | 4.698 | ADP-RIBOSYLATION FACTOR |
| SVS1 | 1.13E-04 | 4.691 | UNKNOWN |
| CDC10 | 1.18E-04 | 4.679 | SEPTIN |
| SUB1 | 1.23E-04 | 4.667 | TRANSCRIPTIONAL COACTIVATOR |
| ECM3 | 1.24E-04 | 4.664 | UNKNOWN |
| MIG1 | 1.31E-04 | -4.647 | TRANSCRIPTIONAL REPRESSOR |
| HRD1 | 1.33E-04 | 4.643 | UNKNOWN; REQUIRED TO DEGRADE MISFOLDED E |
| PAM1 | 1.34E-04 | 4.638 | UNKNOWN; OVEREXPRESSION SUPPRESSES PP2A |
| HOR7 | 1.48E-04 | 4.605 | HYPEROSMOLARITY-RESPONSIVE |
| SSK2 | 1.92E-04 | 4.538 | MAPKKK (MITOGEN-ACTIVATED PROTEIN KINASE |
| YIP2 | 1.93E-04 | 4.534 | UNKNOWN; INTERACTS WITH YPT PROTEIN(S) |
| QRI8 | 2.21E-04 | 4.501 | E2 UB.-CONJUGATING ENZYME |
| Cluster 4 |  |  |  |
| CDC14 | 9.42E-19 | 11.155 | PROTEIN PHOSPHATASE |
| ARP1 | 3.81E-18 | -10.878 | ACTIN-RELATED PROTEIN |
| SSY5 | 1.62E-14 | 9.527 | UNKNOWN |
| TOM71 | 1.15E-10 | -8.042 | OUTER MEMBRANE TRANSLOCASE COMPONENT |
| DPB3 | 4.83E-10 | 7.74 | POLYMERASE EPSILON C SUBUNIT |
| DIG1 | 1.89E-08 | 7.059 | MAP KINASE-ASSOCIATED PROTEIN |
| AAT1 | 3.12E-08 | 6.943 | ASPARTATE AMINOTRANSFERASE, |
| CCP1 | 1.39E-07 | 6.572 | CYTOCHROME-C PEROXIDASE |
| PLM2 | 1.39E-07 | 6.572 | UNKNOWN |
| OPI1 | 3.51E-07 | -6.386 | NEGATIVE REGULATOR OF PHOSPHOLIPID BIOSY |
| ISU2 | 3.88E-07 | 6.347 | UNKNOWN; SIMILAR TO IRON-SULFUR CLUSTER |
| THI13 | 1.04E-06 | 6.124 | UNKNOWN; SIMILAR TO THI5P, THI11P, AND T |
| ESC2 | 1.82E-06 | -5.983 | UNKNOWN |
| ORM1 | 2.60E-06 | 5.905 | UNKNOWN |
| RNR2 | 2.88E-06 | 5.876 | RIBONUCLEOTIDE REDUCTASE |
| NGG1 | 2.88E-06 | -5.872 | HISTONE ACETYLTRANSFERASE COMPLEX SUBUNI |
| TSC10 | 1.06E-05 | -5.595 | 3-KETOSPHINGANINE REDUCTASE |
| GAL83 | 1.39E-05 | -5.533 | COMPONENT OF SNF1 COMPLEX |
| SPS18 | 1.42E-05 | 5.524 | UNKNOWN |
| PDC2 | 1.51E-05 | -5.505 | REGULATOR OF PYRUVATE DECARBOXYLASE GENE |
| HMO1 | 2.48E-05 | -5.395 | NON-HISTONE PROTEIN |
| BRR1 | 3.44E-05 | -5.304 | REQUIRED FOR SNRNP BIOGENESIS |
| RGD1 | 5.20E-05 | -5.205 | GTPASE-ACTIVATING (GAP) PROTEIN (PUTATIV |
| RAD51 | 5.20E-05 | 5.201 | RECOMBINASE |
| YPT10 | 5.62E-05 | -5.18 | UNKNOWN; SIMILAR TO RAB PROTEINS AND OTH |
| SIT4 | 6.17E-05 | -5.155 | TYPE 2A RELATED PROTEIN PHOSPHATASE |
| LRE1 | 6.41E-05 | -5.137 | UNKNOWN |
| SDS22 | 7.11E-05 | -5.106 | GLC7P REGULATORY SUBUNIT |
| CTR3 | 9.78E-05 | 5.023 | COPPER TRANSPORTER |
| TSA1 | 1.13E-04 | 4.973 | THIOL-SPECIFIC |
| CAX4 | 1.13E-04 | 4.971 | UNKNOWN |
| AAD6 | 1.19E-04 | 4.952 | HYPOTHETICAL ARYL-ALCOHOL DEHYDROGENASE |
| AIP2 | 1.26E-04 | 4.935 | ACTIN INTERACTING PROTEIN |
| SOD1 | 1.27E-04 | 4.928 | COPPER-ZINC SUPEROXIDE DISMUTASE |
| AAD3 | 1.42E-04 | 4.897 | HYPOTHETICAL ARYL-ALCOHOL DEHYDROGENASE |
| THI5 | 1.42E-04 | 4.894 | UNKNOWN |
| TFC6 | 1.54E-04 | -4.866 | TFIIIC 91 KD SUBUNIT |
| SIR2 | 1.54E-04 | -4.861 | REULATOR OF SILENCING AT HML, HMR, TELOM |
| SOD2 | 1.54E-04 | 4.861 | MANGANESE SUPEROXIDE DISMUTASE |
| PDC6 | 1.74E-04 | 4.823 | PYRUVATE DECARBOXYLASE 3 |
| HAC1 | 1.86E-04 | -4.801 | TRANSCRIPTION FACTOR |
| NPL4 | 2.45E-04 | 4.726 | NUCLEAR PORE PROTEIN |
| TRP4 | 2.66E-04 | -4.702 | ANTHRANILATE PHOSPHORIBOSYLTRANSFERASE |
| AHT1 | 2.77E-04 | -4.687 | UNKNOWN |
| SPC72 | 2.77E-04 | 4.686 | SPINDLE POLE BODY COMPONENT |
| BOI2 | 2.77E-04 | -4.684 | BINDS BEM1P |
| NPL6 | 2.77E-04 | -4.682 | UNKNOWN |
| SMB1 | 2.77E-04 | -4.679 | U1 SNRNP PROTEIN |
| CYC1 | 3.13E-04 | 4.648 | CYTOCHROME-C ISOFORM 1 |
| SHS1 | 3.54E-04 | -4.617 | SEPTIN (PUTATIVE) |
| RPO21 | 4.83E-04 | -4.533 | RNA POLYMERASE II 215 KD SUBUNIT |
| CDC40 | 5.14E-04 | -4.507 | UNKNOWN |
| CDH1 | 5.25E-04 | -4.499 | CYCLIN DEGRADATION |
| PRP11 | 5.30E-04 | -4.492 | U2, U5, U4/U6 SNRNP PROTEIN |
| CYT2 | 5.30E-04 | 4.491 | CYTOCHROME C1 HEME LYASE |
| SPR6 | 5.56E-04 | -4.475 | UNKNOWN |
| SCS2 | 5.90E-04 | -4.459 | REGULATOR OF INO1 EXPRESSION |
| GNP1 | 7.01E-04 | 4.406 | GLUTAMINE PERMEASE |
| LYS9 | 7.01E-04 | 4.404 | SACCHAROPINE DEHYDROGENASE |
| NUP57 | 7.01E-04 | -4.401 | NUCLEAR PORE PROTEIN |
| BPL1 | 7.76E-04 | -4.37 | BIOTIN:APOPROTEIN LIGASE |
| MRPL33 | 8.48E-04 | 4.344 | RIBOSOMAL PROTEIN, MITOCHONDRIAL L33 |
| ARR3 | 8.51E-04 | -4.339 | ARSENITE TRANSPORTER |
| SCY1 | 9.51E-04 | -4.31 | UNKNOWN |
| MRS5 | 9.84E-04 | -4.299 | INNER MEMBRANE CARRIER PROTEIN |
| Cluster 5 |  |  |  |
| --- |  |  |  |
| Cluster 6 |  |  |  |
| DAL4 | 6.30E-31 | 15.502 | ALLANTOIN PERMEASE |
| DUR3 | 4.58E-30 | 15.09 | UREA PERMEASE |
| AMD2 | 1.49E-20 | 11.687 | PUTATIVE AMIDASE |
| DAL7 | 2.67E-17 | 10.455 | MALATE SYNTHASE |
| YSP3 | 4.48E-16 | 9.963 | SUBTILISIN-LIKE PROTEASE III |
| DAL80 | 1.66E-15 | 9.734 | TRANSCRIPTION FACTOR |
| DAL5 | 2.39E-13 | 8.888 | ALLANTOATE PERMEASE |
| GNP1 | 3.05E-12 | -8.442 | GLUTAMINE PERMEASE |
| MEP2 | 5.85E-12 | 8.314 | AMMONIA PERMEASE |
| ISC10 | 7.75E-12 | 8.243 | UNKNOWN |
| MUP1 | 6.54E-11 | -7.817 | METHIONINE PERMEASE |
| DAL82 | 1.18E-10 | 7.709 | ACTIVATOR OF ALLANTOIN CATABOLIC GENES |
| PIB1 | 2.42E-10 | 7.573 | PHOSPHATIDYLINOSITOL(3)-PHOSPHATE BINDIN |
| DAL2 | 1.81E-09 | 7.186 | ALLANTOICASE |
| TKL1 | 1.93E-09 | -7.168 | TRANSKETOLASE |
| GAP1 | 3.35E-09 | 7.065 | GENERAL AMINO ACID PERMEASE |
| DCG1 | 8.41E-09 | 6.89 | MAY BE INVOLVED IN CATABOLITE REPRESSION |
| DAL1 | 9.62E-09 | 6.855 | ALLANTOINASE |
| BAR1 | 1.30E-08 | -6.781 | ALPHA-FACTOR DEGRADATION |
| CIS3 | 3.15E-08 | -6.599 | UNKNOWN; OVEREXPRESSION SUPPRESSES CIK1 |
| MAS2 | 5.87E-08 | -6.472 | MITOCHONDRIAL PROCESSING PROTEASE SUBUNI |
| LCB2 | 1.35E-07 | -6.305 | SERINE C-PALMITOYLTRANSFERASE SUBUNIT |
| PMU1 | 1.63E-07 | -6.26 | PHOSPHOMUTASE HOMOLOG; SUPPRESSES TS TPS |
| INO2 | 1.70E-07 | 6.246 | TRANSCRIPTION FACTOR |
| VAP1 | 2.20E-07 | -6.19 | AMINO ACID PERMEASE |
| LEA1 | 2.89E-07 | 6.134 | U2 SNRNP PROTEIN |
| MKT1 | 3.53E-07 | -6.089 | RETROVIRAL PROTEASE SIGNATURE PROTEIN |
| CUP1-2 | 5.94E-07 | 5.969 | METALLOTHIONEIN |
| PMT1 | 6.51E-07 | -5.949 | DOLICHYL PHOSPHATE-D-MANNOSE:PROTEIN O-D |
| RAD30 | 8.65E-07 | 5.88 | UNKNOWN |
| ASP3-1 | 8.65E-07 | 5.879 | L-ASPARAGINASE II |
| HSH49 | 9.35E-07 | 5.86 | U2 SNRNP PROTEIN; HUMAN SAP145 HOMOLOG |
| RGM1 | 1.01E-06 | 5.841 | PUTATIVE REPRESSOR |
| PEX15 | 1.08E-06 | 5.821 | INTEGRAL MEMBRANE PROTEIN |
| ARO8 | 1.12E-06 | -5.813 | AROMATIC AMINO ACID AMINOTRANSFERASE |
| PUT1 | 1.12E-06 | 5.81 | PROLINE OXIDASE |
| UGA3 | 1.16E-06 | 5.798 | ACTIVATOR OF GABA CATABOLIC GENES |
| SHC1 | 1.22E-06 | 5.784 | UNKNOWN; MAY BE INVOLVED IN CHITIN SYNTH |
| CUP1-1 | 2.04E-06 | 5.674 | METALLOTHIONEIN |
| ECM37 | 2.14E-06 | 5.661 | UNKNOWN |
| ERG4 | 2.20E-06 | -5.654 | STEROL C-24 REDUCTASE |
| PIG2 | 2.32E-06 | 5.641 | (PUTATIVE) GLC7P REGULATORY SUBUNIT |
| IME1 | 2.69E-06 | 5.609 | TRANSCRIPTION FACTOR |
| ALG7 | 2.93E-06 | -5.589 | UDP-N-ACETYL-GLUCOSAMINE-1-P TRANSFERASE |
| AGA1 | 2.96E-06 | -5.582 | A-AGGLUTININ ANCHOR SUBUNIT |
| RPB2 | 3.45E-06 | -5.547 | RNA POLYMERASE II 140 KDA SUBUNIT |
| YAP5 | 3.87E-06 | 5.518 | BASIC LEU ZIPPER TRANSCRIPTION FACTOR |
| INO1 | 4.37E-06 | 5.487 | L-MYO-INOSITOL-1-PHOSPHATE SYNTHASE |
| STE50 | 4.38E-06 | 5.484 | PHEROMONE RESPONSE |
| PEX10 | 4.58E-06 | 5.474 | INTEGRAL MEMBRANE PROTEIN |
| MPS2 | 4.59E-06 | 5.47 | UNKNOWN; NUCLEAR ENVELOPE/ER PROTEIN |
| PCL7 | 4.90E-06 | 5.456 | CYCLIN |
| YIP1 | 5.01E-06 | -5.448 | UNKNOWN; INTERACTS WITH YPT PROTEIN(S) |
| MUD1 | 5.28E-06 | 5.432 | U1 SNRNP A PROTEIN |
| TOM40 | 5.62E-06 | -5.418 | OUTER MEMBRANE TRANSLOCASE COMPONENT |
| NHP10 | 6.34E-06 | 5.39 | NON-HISTONE PROTEIN |
| ECM39 | 6.39E-06 | -5.386 | UNKNOWN |
| KTR3 | 6.90E-06 | -5.367 | PUTATIVE ALPHA-1,2-MANNOSYLTRANSFERASE |
| TEP1 | 7.03E-06 | 5.361 | PROTEIN PHOSPHATASE |
| ASF2 | 7.39E-06 | 5.349 | ANTI-SILENCING PROTEIN |
| LEE1 | 8.56E-06 | 5.316 | UNKNOWN |
| ASP3-2 | 8.56E-06 | 5.314 | L-ASPARAGINASE II |
| SRL1 | 1.23E-05 | -5.226 | UNKNOWN; SIMILAR TO SVS1P; SUPPRESSOR OF |
| UGX2 | 1.23E-05 | 5.223 | UNKNOWN |
| SHE9 | 1.23E-05 | -5.22 | UNKNOWN |
| ELM1 | 1.23E-05 | 5.219 | PROTEIN KINASE |
| MAK31 | 1.23E-05 | 5.219 | UNKNOWN |
| SIP18 | 1.52E-05 | 5.167 | INDUCED BY OSMOTIC STRESS |
| STE6 | 1.58E-05 | -5.156 | A-FACTOR EXPORTER (ABC SUPERFAMILY) |
| SFI1 | 1.63E-05 | 5.146 | UNKNOWN |
| TAT2 | 1.70E-05 | -5.133 | TRYPTOPHAN PERMEASE |
| MET30 | 1.88E-05 | 5.101 | F-BOX TRANSCRIPTION FACTOR |
| EGT2 | 2.04E-05 | -5.08 | UNKNOWN |
| GAS1 | 2.17E-05 | -5.065 | CELL SURFACE GLYCOPROTEIN |
| SAC1 | 2.37E-05 | -5.043 | ER/GOLGI ATP/ADP EXCHANGER |
| STN1 | 2.64E-05 | 5.017 | ASSSOCIATES WITH CDC13P |
| ASN2 | 2.66E-05 | -5.013 | ASPARAGINE SYNTHETASE |
| RRN10 | 2.72E-05 | 5.006 | COMPONENT OF UPSTREAM ACTIVATION FACTOR |
| MGT1 | 2.98E-05 | 4.983 | 6-O-METHYLGUANINE-DNA METHYLASE |
| YHR160C | 2.98E-05 | 4.983 | PEX18 |
| FUS1 | 3.06E-05 | -4.973 | SH3 DOMAIN PROTEIN |
| MAF1 | 3.24E-05 | 4.96 | UNKNOWN |
| APT2 | 3.25E-05 | 4.958 | UNKNOWN; SIMILAR TO ADENINE PHOSPHORIBOS |
| EXG2 | 3.73E-05 | -4.922 | EXO-BETA-1,3-GLUCANASE |
| PLB3 | 3.76E-05 | -4.917 | PHOSPHOLIPASE B |
| ITR2 | 3.85E-05 | -4.911 | INOSITOL PERMEASE |
| COP1 | 4.69E-05 | -4.859 | VESICLE COAT COMPONENT |
| GLN3 | 4.69E-05 | 4.858 | TRANSCRIPTION FACTOR |
| WSC2 | 4.73E-05 | -4.855 | ALPHA-1,4-GLUCAN-GLUCOSIDASE |
| SPT3 | 4.96E-05 | 4.842 | HISTONE ACETYLTRANSFERASE COMPLEX SUBUNI |
| STE2 | 5.17E-05 | -4.831 | ALPHA-FACTOR RECEPTOR |
| SCP160 | 5.18E-05 | -4.829 | UNKNOWN |
| CWP1 | 5.41E-05 | -4.818 | BETA-1,6-GLUCAN ACCEPTOR |
| SLC1 | 5.58E-05 | -4.807 | FATTY ACYLTRANSFERASE |
| CSE4 | 6.06E-05 | 4.785 | HISTONE-RELATED |
| CDC3 | 6.33E-05 | -4.773 | SEPTIN |
| ECM13 | 6.79E-05 | 4.756 | UNKNOWN |
| CTS1 | 6.85E-05 | -4.752 | ENDOCHITINASE |
| HPA2 | 6.88E-05 | 4.75 | HISTONE ACETYLTRANSFERASE COMPLEX SUBUNI |
| Cluster 7 |  |  |  |
| JEN1 | 0.00E+00 | 21.104 | LACTATE TRANSPORTER |
| PCK1 | 4.74E-39 | 18.197 | PHOSPHOENOLPYRUVATE CARBOXYKINASE |
| CAT2 | 5.48E-27 | 13.738 | CARNITINE O-ACETYLTRANSFERASE |
| FBP1 | 6.18E-27 | 13.684 | FRUCTOSE-1,6-BISPHOSPHATASE |
| ACH1 | 6.18E-27 | 13.682 | ACETYL-COA HYDROLASE |
| CIT3 | 1.31E-25 | 13.201 | CITRATE SYNTHASE |
| CTA1 | 2.25E-25 | 13.104 | CATALASE A |
| SPS100 | 2.44E-24 | 12.728 | SPORE WALL MATURATION PROTEIN |
| IDP3 | 5.77E-23 | 12.223 | ISOCITRATE DEHYDROGENASE |
| SFC1 | 6.91E-23 | 12.185 | MITOCHONDRIAL SUCCINATE-FUMARATE CARRIER |
| PXA1 | 7.03E-23 | 12.173 | LONG-CHAIN FATTY ACID TRANSPORTER, ABC F |
| IDP2 | 1.69E-22 | 12.03 | ISOCITRATE DEHYDROGENASE |
| ISF1 | 3.25E-22 | 11.922 | INTERACTS WITH NAM7P |
| IME4 | 1.77E-20 | 11.28 | TRANSCRIPTION FACTOR |
| MLS1 | 1.57E-19 | 10.935 | MALATE SYNTHASE |
| GAC1 | 4.13E-18 | 10.418 | GLC7P REGULATORY SUBUNIT |
| CDC10 | 4.77E-17 | -10.013 | SEPTIN |
| CYB2 | 5.45E-17 | 9.981 | CYTOCHROME B2 |
| FUN34 | 5.86E-17 | 9.965 | UNKNOWN |
| FOX2 | 6.59E-17 | 9.941 | PEROXISOMAL BETA-OXIDATION PROTEIN |
| GUT1 | 6.67E-17 | 9.934 | GLYCEROL KINASE |
| SIP18 | 1.19E-16 | 9.819 | INDUCED BY OSMOTIC STRESS |
| REG2 | 1.27E-16 | 9.804 | (PUTATIVE) GLC7P REGULATORY SUBUNIT |
| MBR1 | 1.46E-16 | 9.779 | UNKNOWN |
| RPS7B | 2.26E-16 | -9.702 | RIBOSOMAL PROTEIN S7B |
| STL1 | 2.26E-16 | 9.698 | HEXOSE TRANSPORTER (PUTATIVE) |
| ACS1 | 2.38E-16 | 9.686 | ACETYL-COA SYNTHETASE |
| QCR9 | 2.59E-16 | 9.67 | UBIQUINOL CYTOCHROME-C REDUCTASE SUBUNIT |
| AST2 | 3.45E-16 | 9.621 | TARGETS PLASMA MEMBRANE ATPASE |
| SIP4 | 7.87E-16 | 9.487 | TRANSCRIPTION FACTOR |
| COX13 | 1.52E-15 | 9.375 | CYTOCHROME-C OXIDASE SUBUNIT VIA |
| POX1 | 1.68E-15 | 9.356 | ACYL-COA OXIDASE |
| YHR160C | 3.41E-15 | 9.231 | PEX18 |
| BFR1 | 4.74E-15 | -9.175 | UNKNOWN |
| ERG6 | 5.58E-15 | -9.144 | S-ADENOSYL-METHIONINE DELTA-24-STEROL-C- |
| VMA5 | 5.86E-15 | -9.13 | VACUOLAR H+-ATPASE V1 SECTOR SUBUNIT |
| ECM37 | 9.78E-15 | 9.04 | UNKNOWN |
| THS1 | 1.55E-14 | -8.955 | TRNA SYNTHETASE, THREONYL |
| RPS9A | 1.72E-14 | -8.935 | RIBOSOMAL PROTEIN S9A |
| SNF3 | 2.93E-14 | 8.842 | GLUCOSE PERMEASE |
| KRS1 | 3.43E-14 | -8.811 | TRNA SYNTHETASE, LYSYL |
| ICL1 | 4.96E-14 | 8.747 | ISOCITRATE LYASE |
| KGD2 | 5.39E-14 | 8.728 | 2-OXOGLUTARATE DEHYDROGENASE |
| FRS1 | 8.12E-14 | -8.655 | TRNA SYNTHETASE, PHENYLALANYL, SUBUNIT |
| ARO7 | 8.79E-14 | -8.64 | CHORISMATE MUTASE |
| PIG2 | 8.85E-14 | 8.637 | (PUTATIVE) GLC7P REGULATORY SUBUNIT |
| GRE1 | 9.93E-14 | 8.612 | UNKNOWN; INDUCED BY OSMOTIC STRESS |
| SDH2 | 1.01E-13 | 8.608 | SUCCINATE DEHYDROGENASE |
| TYS1 | 1.23E-13 | -8.571 | TRNA SYNTHETASE, TYROSYL |
| CDC3 | 1.70E-13 | -8.513 | SEPTIN |
| RPL22A | 1.95E-13 | -8.489 | RIBOSOMAL PROTEIN L22A |
| HTS1 | 1.98E-13 | -8.484 | TRNA SYNTHETASE, HISTIDYL |
| ECM8 | 3.76E-13 | 8.376 | UNKNOWN |
| COX5A | 4.00E-13 | 8.365 | CYTOCHROME-C OXIDASE SUBUNIT VA |
| RNA1 | 5.56E-13 | -8.306 | GTPASE ACTIVATING PROTEIN FOR GSP1P |
| IPP1 | 5.73E-13 | -8.299 | INORGANIC PYROPHOSPHATASE, CYTOPLASMIC |
| CRS5 | 8.66E-13 | 8.225 | METALLOTHIONEIN-LIKE PROTEIN |
| SPS19 | 9.99E-13 | 8.198 | PEROXISOMAL 2,4-DIENOYL-COA REDUCTASE |
| COX7 | 1.03E-12 | 8.191 | CYTOCHROME-C OXIDASE, SUBUNIT VII |
| PHO13 | 1.12E-12 | -8.175 | PROTEIN PHOSPHATASE AND 4-NITROPHENYLPHO |
| ALP1 | 1.91E-12 | 8.081 | BASIC AMINO ACID PERMEASE |
| QCR8 | 3.11E-12 | 7.995 | UBIQUINOL--CYTOCHROME-C REDUCTASE SUBUNI |
| UBC4 | 4.79E-12 | -7.917 | E2 UB.-CONJUGATING ENZYME |
| GIS1 | 6.13E-12 | 7.87 | UNKNOWN |
| FUR1 | 6.13E-12 | -7.869 | URACIL PHOSPHORIBOSYLTRANSFERASE |
| GSP1 | 6.67E-12 | -7.854 | GTP-BINDING PROTEIN, RAS SUPERFAMILY |
| HXT5 | 6.77E-12 | 7.849 | HEXOSE PERMEASE |
| MAL11 | 6.83E-12 | 7.847 | ALPHA-GLUCOSIDE PERMEASE |
| HCR1 | 6.94E-12 | -7.842 | UNKNOWN |
| CDH1 | 7.46E-12 | 7.829 | CYCLIN DEGRADATION |
| ALG5 | 8.49E-12 | -7.804 | UDP-GLUCOSE:DOLICHYL-PHOSPHATE GLUCOSYLT |
| RPL11A | 8.97E-12 | -7.792 | RIBOSOMAL PROTEIN L11A |
| PEX3 | 9.69E-12 | 7.777 | INTEGRAL MEMBRANE PROTEIN |
| APL6 | 1.10E-11 | -7.755 | AP-3 COMPLEX SUBUNIT |
| PHO88 | 1.13E-11 | -7.747 | REGULATOR OF PHO81 |
| SRP68 | 1.24E-11 | -7.729 | SIGNAL RECOGNITION PARTICLE SUBUNIT |
| BOP2 | 1.24E-11 | 7.728 | UNKNOWN; BYPASS OF PAM1 |
| ACB1 | 1.38E-11 | -7.708 | ACYL-COA ESTER TRANSPORTER |
| COX12 | 1.51E-11 | 7.689 | CYTOCHROME-C OXIDASE, SUBUNIT VIB |
| SEC63 | 2.08E-11 | -7.632 | ER PROTEIN TRANSLOCATION SUBCOMPLEX SUBU |
| ICL2 | 2.14E-11 | 7.626 | ISOCITRATE LYASE, NONFUNCTIONAL |
| EPT1 | 2.17E-11 | -7.622 | SN-1,2-DIACYLGLYCEROL ETHANOLAMINE- AND |
| RHO2 | 2.56E-11 | -7.59 | GTP-BINDING PROTEIN, RHO FAMILY |
| HAM1 | 2.86E-11 | -7.568 | UNKNOWN |
| RAD4 | 2.91E-11 | 7.564 | REPAIROSOME COMPONENT |
| MES1 | 3.05E-11 | -7.555 | TRNA SYNTHETASE, METHIONYL |
| SEC72 | 3.51E-11 | -7.524 | ER PROTEIN TRANSLOCATION SUBCOMPLEX SUBU |
| NOT5 | 4.72E-11 | -7.47 | GLOBAL NEGATIVE REGULATOR CPMPLEX COMPON |
| RPL21B | 5.08E-11 | -7.456 | RIBOSOMAL PROTEIN L21B |
| SSY1 | 5.14E-11 | 7.453 | REGULATOR OF TRANSPORTERS |
| GRX3 | 5.23E-11 | -7.449 | UNKNOWN; SIMILAR TO YER174P, PROBABLE TH |
| SLZ1 | 5.74E-11 | 7.429 | UNKNOWN |
| SAC1 | 6.12E-11 | -7.417 | ER/GOLGI ATP/ADP EXCHANGER |
| SPO20 | 6.24E-11 | 7.412 | SNAP 25 HOMOLOG; PROSPORE MEMBRANE FORMA |
| PXA2 | 6.45E-11 | 7.404 | PEROXISOMAL FATTY ACID TRANSPORTER, ABC |
| RPL24B | 7.74E-11 | -7.371 | RIBOSOMAL PROTEIN L24B |
| SRP1 | 7.87E-11 | -7.365 | ALPHA-KARYOPHERIN |
| GLG2 | 8.02E-11 | 7.361 | GLYCOGEN SYNTHESIS INITIATOR |
| RET3 | 8.07E-11 | -7.359 | VESICLE COAT COMPONENT |
| Cluster 8 |  |  |  |
| SUL1 | 0.00E+00 | 25.058 | SULFATE PERMEASE |
| DAL3 | 0.00E+00 | 20.21 | UREIDOGLYCOLATE HYDROLASE |
| MET3 | 0.00E+00 | 19.875 | SULFATE ADENYLYLTRANSFERASE |
| MET10 | 4.16E-38 | 17.912 | SULFITE REDUCTASE SUBUNIT |
| --- | 4.50E-37 | 17.496 | PUTATIVE ALPHA-KETOISOCAPROATE REDUCTASE |
| SUL2 | 1.45E-32 | 15.794 | SULFATE PERMEASE |
| MET2 | 9.54E-31 | 15.128 | HOMOSERINE O-ACETYLTRANSFERASE |
| MET32 | 9.54E-31 | 15.113 | TRANSCRIPTION FACTOR |
| RAD59 | 1.76E-27 | 13.947 | UNKNOWN |
| MET16 | 5.51E-26 | 13.409 | 3'-PHOSPHOADENYLYLSULFATE REDUCTASE |
| ECM17 | 1.85E-25 | 13.213 | UNKNOWN |
| MET1 | 6.09E-25 | 13.02 | SIROHEME SYNTHASE |
| DAL5 | 1.22E-22 | 12.193 | ALLANTOATE PERMEASE |
| DAL2 | 1.74E-22 | 12.122 | ALLANTOICASE |
| ARG1 | 2.97E-22 | 12.032 | ARGINOSUCCINATE SYNTHETASE |
| MEP2 | 6.84E-22 | 11.897 | AMMONIA PERMEASE |
| BAP2 | 1.74E-21 | -11.747 | BRANCHED-CHAIN AMINO ACID PERMEASE |
| LYS1 | 4.46E-21 | 11.596 | SACCHAROPINE DEHYDROGENASE |
| GDH1 | 5.09E-21 | 11.569 | GLUTAMATE DEHYDROGENASE |
| GAP1 | 6.85E-20 | 11.164 | GENERAL AMINO ACID PERMEASE |
| LEU2 | 1.02E-19 | 11.096 | BETA-ISOPROPYL-MALATE DEHYDROGENASE |
| DAL7 | 1.95E-19 | 10.991 | MALATE SYNTHASE |
| OAC1 | 6.73E-19 | 10.788 | OXALOACETATE CARRIER |
| LEU1 | 1.44E-18 | 10.665 | 3-ISOPROPYLMALATE DEHYDRATASE |
| MET28 | 3.26E-17 | 10.169 | TRANSCRIPTIONAL ACTIVATOR |
| CTP1 | 7.71E-17 | 10.03 | MITOCHONDRIAL CITRATE TRANSPORTER |
| LYS14 | 1.01E-16 | 9.978 | TRANSCRIPTION FACTOR |
| LYS12 | 9.36E-16 | 9.616 | HOMO-ISOCITRATE DEHYDROGENASE |
| MET22 | 9.36E-16 | 9.613 | 3'(2')5'-BISPHOSPHATE NUCLEOTIDASE |
| MET14 | 2.43E-15 | 9.453 | ADENYLYLSULFATE KINASE |
| ARG3 | 2.55E-15 | 9.441 | ORNITHINE CARBAMOYLTRANSFERASE |
| DAL80 | 5.14E-15 | 9.325 | TRANSCRIPTION FACTOR |
| ARG5,6 | 8.15E-15 | 9.248 | ACETYLGLUTAMATE KINASE AND ACETYLGLUTAMY |
| DCG1 | 4.53E-14 | 8.964 | MAY BE INVOLVED IN CATABOLITE REPRESSION |
| CPS1 | 1.13E-13 | 8.808 | VACUOLAR CARBOXYPEPTIDASE YSCS |
| GAT1 | 1.14E-13 | 8.803 | TRANSCRIPTION FACTOR |
| CHA1 | 2.12E-13 | -8.698 | L-SERINE/L-THREONINE DEAMINASE |
| ADE3 | 3.00E-13 | 8.638 | C1-5,6,7,8-TETRAHYDROFOLATE SYNTHASE |
| BAP3 | 3.27E-13 | -8.621 | BRANCHED-CHAIN AMINO ACID PERMEASE |
| HIS7 | 4.13E-13 | 8.579 | GLUTAMINE AMIDOTRANSFERASE:CYCLASE |
| SER1 | 8.31E-13 | 8.461 | PHOSPHOSERINE |
| MEP1 | 1.10E-12 | 8.41 | AMMONIA PERMEASE |
| YGR239C | 2.20E-12 | 8.289 | PEX21 |
| MNN4 | 2.44E-12 | 8.269 | PHOSPHATIDYLINOSITOL KINASE HOMOLG |
| VAP1 | 8.34E-11 | -7.661 | AMINO ACID PERMEASE |
| CAN1 | 1.80E-10 | 7.525 | BASIC AMINO ACID PERMEASE |
| GLN1 | 1.80E-10 | 7.522 | GLUTAMINE SYNTHETASE |
| GLN3 | 1.91E-10 | 7.508 | TRANSCRIPTION FACTOR |
| ADE12 | 4.19E-10 | 7.369 | ADENYLOSUCCINATE SYNTHETASE |
| NIT1 | 5.80E-10 | 7.308 | NITRILASE |
| MET17 | 7.34E-10 | 7.264 | O-ACETYLHOMOSERINE SULFHYDRYLASE |
| SPS4 | 8.58E-10 | 7.231 | PUTATIVE CELL WALL COMPONENT |
| UGA3 | 8.84E-10 | 7.224 | ACTIVATOR OF GABA CATABOLIC GENES |
| TAT2 | 8.93E-10 | -7.219 | TRYPTOPHAN PERMEASE |
| MET13 | 1.22E-09 | 7.161 | METHYLENETETRAHYDROFOLATE REDUCTASE, PUT |
| ADE2 | 1.27E-09 | 7.151 | PHOSPHORIBOSYLAMINOIMIDAZOLE CARBOXYLASE |
| HIS4 | 3.44E-09 | 6.97 | HISTIDINOL DEHYDROGENASE |
| YAP5 | 9.69E-09 | 6.776 | BASIC LEU ZIPPER TRANSCRIPTION FACTOR |
| GZF3 | 1.03E-08 | 6.762 | TRANSCRIPTION FACTOR |
| PRK1 | 1.13E-08 | 6.744 | PROTEIN KINASE |
| ARG8 | 1.21E-08 | 6.726 | ACETYLORNITHINE AMINOTRANSFERASE |
| ASP3-3 | 1.48E-08 | 6.683 | L-ASPARAGINASE II |
| HIS3 | 1.49E-08 | 6.681 | IMIDAZOLEGLYCEROL-PHOSPHATE DEHYDRATASE |
| DUR3 | 1.70E-08 | 6.653 | UREA PERMEASE |
| DAL1 | 1.76E-08 | 6.645 | ALLANTOINASE |
| CUS1 | 2.15E-08 | 6.603 | U2 SNRNP PROTEIN |
| HIS5 | 2.17E-08 | 6.599 | HISTIDINOL-PHOSPHATE AMINOTRANSFERASE |
| ARG4 | 2.85E-08 | 6.547 | ARGININOSUCCINATE LYASE |
| YHM1 | 3.32E-08 | 6.514 | MITOCHONDRIAL CARRIER FAMILY |
| GIS3 | 4.72E-08 | -6.447 | UNKNOWN |
| ASP3-1 | 4.97E-08 | 6.43 | L-ASPARAGINASE II |
| CIT2 | 1.17E-07 | 6.264 | PEROXISOMAL CITRATE SYNTHASE |
| SAM2 | 1.19E-07 | 6.26 | REGULATOR; S-ADENOSYLMETHIONINE SYNTHETA |
| RHO5 | 1.84E-07 | 6.174 | RHO FAMILY GTPASE |
| LYS9 | 3.45E-07 | 6.041 | SACCHAROPINE DEHYDROGENASE |
| HST2 | 4.19E-07 | -5.997 | SIMILAR TO SIR2P |
| ORT1 | 5.94E-07 | 5.927 | AMINO ACID TRANSPORTER |
| DLD1 | 6.73E-07 | 5.9 | D-LACTATE DEHYDROGENASE |
| CAR1 | 6.95E-07 | -5.892 | ARGINASE |
| ASP3-4 | 1.11E-06 | 5.792 | L-ASPARAGINASE II |
| YHM2 | 1.28E-06 | 5.762 | SUPPRESSES HM MUTANT |
| LYS20 | 1.32E-06 | 5.754 | HOMOCITRATE SYNTHASE |
| KAR1 | 1.62E-06 | 5.709 | NUCLEAR FUSION; ALSO SPINDLE |
| MTD1 | 1.71E-06 | 5.697 | NAD-DEPENDENT 5,10-METHYLENETETRAHYDRAFO |
| ASP3-2 | 1.95E-06 | 5.667 | L-ASPARAGINASE II |
| MED4 | 2.04E-06 | 5.655 | RNA POLYMERASE II MEDIATOR SUBUNIT |
| MMD1 | 2.11E-06 | -5.647 | UNKNOWN |
| PDS1 | 3.85E-06 | 5.514 | ANAPHASE INHIBITOR (PUTATIVE) |
| LYS4 | 4.05E-06 | 5.498 | HOMOACONITASE |
| GDH3 | 6.10E-06 | 5.409 | NADP-GLUTAMATE DEHYDROGENASE |
| MUP1 | 6.36E-06 | -5.398 | METHIONINE PERMEASE |
| PRE8 | 1.21E-05 | -5.261 | 20S PROTEASOME SUBUNIT Y7 (ALPHA2 |
| MUP3 | 1.33E-05 | 5.239 | METHIONINE PERMEASE |
| ECM40 | 1.39E-05 | 5.225 | ACETYLORNITHINE ACETYLTRANSFERASE |
| IDP1 | 1.46E-05 | 5.212 | ISOCITRATE DEHYDROGENASE (NADP+) |
| AGP1 | 1.80E-05 | -5.16 | AMINO ACID PERMEASE |
| ADE4 | 2.22E-05 | 5.11 | AMIDOPHOSPHORIBOSYLTRANSFERASE |
| ZRT1 | 2.23E-05 | -5.107 | HIGH-AFFINITY ZINC TRANSPORTER |
| HOM3 | 2.85E-05 | 5.049 | ASPARTATE KINASE |
| ISA1 | 2.98E-05 | 5.038 | UNKNOWN; IRON SULFUR ASSEMBLY -- ISCA/NI |
| Cluster 9 |  |  |  |
| MEP3 | 4.48E-08 | 7.291 | AMMONIA PERMEASE |
| CDS1 | 2.55E-06 | 6.428 | CDP-DIACYLGLYCEROL SYNTHASE |
| FCY22 | 6.51E-06 | 6.116 | PURINE-CYTOSINE PERMEASE |
| PHO3 | 6.12E-05 | 5.462 | ACID PHOSPHATASE, CONSTITUTIVE |
| SML1 | 6.81E-05 | 5.421 | REGULATOR OF RIBONUCLEOTIDE REDUCTASE (P |
| CUS2 | 9.10E-05 | -5.329 | UNKNOWN |
| HOL1 | 9.15E-05 | 5.312 | (PUTATIVE) MULTIDRUG RESISTANCE PERMEASE |
| MRS2 | 1.03E-04 | 5.274 | UNKNOWN |
| ELO1 | 1.05E-04 | 5.256 | FATTY ACID ELONGATION PROTEIN |
| RPN5 | 1.05E-04 | -5.245 | 26S PROTEASOME SUBUNIT |
| DJP1 | 1.82E-04 | -5.096 | UNKNOWN |
| LEO1 | 1.85E-04 | -5.078 | UNKNOWN |
| BUD9 | 2.30E-04 | 4.985 | UNKNOWN |
| PDA1 | 2.52E-04 | 4.941 | PYRUVATE DEHYDROGENASE |
| UTR5 | 2.80E-04 | 4.911 | UNKNOWN |
| PTA1 | 3.36E-04 | 4.855 | UNKNOWN |
| MTR3 | 3.57E-04 | 4.828 | NUCLEOLAR PROTEIN |
| TFG2 | 3.72E-04 | -4.813 | TFIIF 54 KD SUBUNIT |
| RTT103 | 4.99E-04 | -4.726 | UNKNOWN; SIMILAR TO SPT8P |
| NUF1 | 4.99E-04 | -4.721 | SPINDLE POLE BODY COMPONENT |
| DBP6 | 5.12E-04 | 4.71 | RNA HELICASE, PUTATIVE |
| CDC37 | 5.34E-04 | -4.694 | CHAPERONE |
| RNA14 | 6.27E-04 | -4.639 | CLEAVAGE/POLYADENYLATION FACTOR CF I COM |
| ENT1 | 6.27E-04 | -4.631 | UNKNOWN; EPSIN HOMOLOG |
| DIT2 | 6.74E-04 | 4.609 | SYNTHESIZES DITYROSINE |
| ARG80 | 7.12E-04 | 4.587 | TRANSCRIPTION FACTOR |
| Cluster 10 |  |  |  |
| NAM9 | 7.56E-09 | 7.649 | RIBOSOMAL PROTEIN, MITOCHONDRIAL S4 (PUT |
| TAO3 | 6.91E-07 | 6.723 | UNKNOWN; TRANSCRIPIOTNAL ACTIVATOR OF OC |
| NCA2 | 1.63E-04 | 5.441 | REGULATES ATP6P AND ATP8P SYNTHESIS |
| MRPL4 | 1.92E-04 | 5.343 | RIBOSOMAL PROTEIN, MITOCHONDRIAL L4 |
| MRPL38 | 1.92E-04 | 5.334 | RIBOSOMAL PROTEIN, MITOCHONDRIAL L38 |
| ATP20 | 9.43E-04 | 4.932 | MITOCHONDRIAL ATP SYNTHASE SUBUNIT |
| Cluster 11 |  |  |  |
| HAP5 | 7.88E-13 | -9.113 | TRANSCRIPTION FACTOR |
| ADH3 | 2.58E-10 | 7.977 | ALCOHOL DEHYDROGENASE III, MITOCHONDRIAL |
| PGI1 | 4.04E-10 | 7.852 | GLUCOSE-6-PHOSPHATE ISOMERASE |
| KRE2 | 9.26E-10 | 7.645 | ALPHA-1,2-MANNOSYLTRANSFERASE |
| APE3 | 9.26E-10 | 7.64 | VACUOLAR AMINOPEPTIDASE Y |
| TFP1 | 1.44E-08 | 7.116 | VACUOLAR H+-ATPASE SUBUNIT |
| MDL2 | 1.44E-08 | -7.107 | ATP-BINDING CASSETTE (ABC) FAMILY |
| WBP1 | 1.63E-08 | 7.064 | OLIGOSACCHARYLTRANSFERASE COMPLEX SUBUNI |
| IDS2 | 4.41E-08 | -6.834 | PUTATIVE ACTIVATOR OF IME2P |
| VMA2 | 5.68E-08 | 6.757 | 58 KD REGULATORY SUBUNIT |
| RSC4 | 1.10E-07 | 6.622 | CHROMATIN REMODELING COMPLEX SUBUNIT |
| HMGS | 1.26E-07 | 6.586 | 3-HYDROXY-3-METHYLGLUTARYL COENZYME A SY |
| LEU4 | 2.35E-06 | 5.955 | 2-ISOPROPYLMALALATE SYNTHASE |
| BMH2 | 2.47E-06 | 5.937 | SUPPRESSES CLATHRIN DEFICIENCY |
| ERG24 | 2.96E-06 | 5.885 | C-14 STEROL REDUCTASE |
| HYS2 | 2.99E-06 | -5.875 | POLYMERASE DELTA 55 KD SUBUNIT |
| HOG1 | 3.20E-06 | 5.855 | MAPK (MITOGEN-ACTIVATED PROTEIN KINASE) |
| ABD1 | 5.32E-06 | 5.725 | MRNA CAP METHYLTRANSFERASE |
| MSS4 | 5.37E-06 | -5.718 | PHOSPHATIDYLINOSITOL 4-PHOSPHATE KINASE |
| BIK1 | 5.96E-06 | 5.69 | MICROTUBULE-ASSOCIATED PROTEIN |
| RPS19B | 8.28E-06 | 5.606 | RIBOSOMAL PROTEIN S19B |
| SMP3 | 9.38E-06 | 5.564 | INTEGRAL MEMBRANE, PROTEIN KINASE C PATH |
| GSF2 | 1.11E-05 | 5.524 | UNKNOWN |
| PET9 | 1.14E-05 | 5.513 | MITOCHONDRIAL ADP/ATP TRANSLOCATOR |
| PBN1 | 1.25E-05 | -5.49 | PROTEASE B |
| RFT1 | 1.65E-05 | 5.427 | UNKNOWN |
| MNN2 | 1.80E-05 | 5.394 | GOLGI ALPHA-1,2-MANNOSYLTRANSFERASE (PUT |
| GCD6 | 2.18E-05 | 5.347 | TRANSLATION INITIATION FACTOR EIF2B SUBU |
| YPD1 | 3.88E-05 | -5.215 | TWO-COMPONENT PHOSPHORELAY INTERMEDIATE |
| SEC12 | 4.37E-05 | -5.185 | ER-TO-GOLGI GDP/GTP EXCHANGE FACTOR |
| AAT2 | 4.76E-05 | 5.162 | ASPARTATE AMINOTRANFERASE, |
| VMA8 | 5.08E-05 | 5.144 | VACUOLAR H+-ATPASE V1 DOMAIN D SUBUNIT |
| RPE1 | 5.49E-05 | 5.123 | RIBULOSE-5-PHOSPHATE 3-EPIMERASE |
| SMT4 | 6.51E-05 | -5.082 | SUPPRESSES MIF2 MUTATION |
| TFB4 | 7.18E-05 | 5.056 | TFIIH 37 KD SUBUNIT |
| HXK2 | 9.03E-05 | 4.998 | HEXOKINASE II |
| CCT8 | 1.03E-04 | 4.962 | CYTOPLASMIC CHAPERONIN COMPLEX |
| SLD2 | 1.11E-04 | 4.942 | UNKNOWN; INTERACTS WITH DPB11P |
| SEC53 | 1.12E-04 | 4.937 | PHOSPHOMANNOMUTASE |
| CTH1 | 1.33E-04 | -4.887 | UNKNOWN |
| ALG8 | 1.33E-04 | 4.882 | GLYCOSYLTRANSFERASE |
| ASN2 | 1.33E-04 | 4.881 | ASPARAGINE SYNTHETASE |
| BET2 | 1.34E-04 | 4.876 | GERANYLGERANYLTRANSFERASE TYPE II BETA S |
| GCN20 | 1.61E-04 | 4.825 | ACTIVATOR OF GCN2P KINASE; ABC SUPERFAMI |
| RPL27A | 1.63E-04 | 4.819 | RIBOSOMAL PROTEIN L27A |
| ISR1 | 1.69E-04 | 4.804 | PROTEIN KINASE |
| CHK1 | 1.72E-04 | 4.797 | DNA DAMAGE CHECKPOINT PROTEIN KINASE |
| MCM21 | 3.03E-04 | -4.656 | UNKNOWN |
| CAF4 | 3.79E-04 | 4.6 | COMPONENT OF CCR4 TRANSCRIPTIONAL COMPLE |
| OST1 | 3.83E-04 | 4.592 | OLIGOSACCHARYLTRANSFERASE COMPLEX SUBUNI |
| CCT3 | 3.87E-04 | 4.587 | CYTOPLASMIC CHAPERONIN COMPLEX |
| SLU7 | 4.05E-04 | -4.573 | 3' SPLICE SITE SELECTION |
| RNR4 | 4.34E-04 | 4.547 | RIBONUCLEOTIDE REDUCTASE |
| SAH1 | 4.34E-04 | 4.546 | S-ADENOSYL-L-HOMOCYSTEINE HYDROLASE |
| MCX1 | 4.58E-04 | -4.521 | CHAPERONE, MITOCHONDRIAL (PUTATIVE) |
| NMD4 | 4.58E-04 | 4.52 | NAM7P/UPF1P-INTERACTING PROTEIN |
| PDI1 | 4.58E-04 | 4.518 | PROTEIN DISULFIDE ISOMERASE |
| ERG20 | 4.95E-04 | 4.496 | FARNESYL-PYROPHOSPHATE SYNTHETASE |
| TIF35 | 4.95E-04 | 4.495 | TRANSLATION INITIATION FACTOR EIF3 SUBUN |
| BFA1 | 5.50E-04 | -4.46 | UNKNOWN |
| FUI1 | 5.74E-04 | 4.447 | URIDINE PERMEASE |
| SER2 | 5.87E-04 | 4.441 | PHOSPHOSERINE PHOSPHATASE |
| HXT1 | 5.95E-04 | 4.434 | HEXOSE PERMEASE |
| PPA1 | 6.02E-04 | 4.427 | VACUOLAR H+-ATPASE PROTEOLIPID PROTEIN |
| SLY41 | 6.08E-04 | 4.421 | UNKNOWN; SUPPRESSES YPT1 NULL |
| TUB2 | 6.08E-04 | 4.42 | BETA-TUBULIN |
| ADE13 | 6.13E-04 | 4.415 | ADENYLOSUCCINATE LYASE |
| CDC8 | 6.39E-04 | -4.401 | THYMIDYLATE KINASE |
| SEC27 | 6.40E-04 | 4.398 | VESICLE COAT COMPONENT |
| PCH2 | 6.44E-04 | 4.395 | UNKNOWN |
| HNM1 | 6.52E-04 | 4.39 | CHOLINE PERMEASE |
| NIT2 | 7.38E-04 | -4.355 | NITRILASE |
| ERP3 | 7.38E-04 | -4.354 | UNKNOWN |
| QRI2 | 7.41E-04 | 4.347 | UNKNOWN |
| PHO86 | 7.41E-04 | 4.347 | INORGANIC PHOSPHATE PERMEASE |
| CDC7 | 8.20E-04 | 4.313 | S PHASE PROTEIN KINASE |
| POT1 | 8.20E-04 | -4.312 | PEROXISOMAL 3-OXOACYL COA THIOLASE |
| GPI1 | 8.39E-04 | 4.304 | N-ACETYLGLUCOSAMINYLPHOSPHATIDYLINOSITOL |
| RNR2 | 8.98E-04 | 4.281 | RIBONUCLEOTIDE REDUCTASE |
| SPB4 | 9.49E-04 | 4.261 | RNA HELICASE |
| GRR1 | 9.60E-04 | -4.248 | CYCLIN F BOX PROTEIN |
| ARO8 | 9.60E-04 | 4.248 | AROMATIC AMINO ACID AMINOTRANSFERASE |
| COS8 | 9.80E-04 | 4.241 | UNKNOWN; SIMILAR TO SUBTELOMERICALLY-ENC |
| KTR1 | 9.90E-04 | 4.237 | MANNOSYLTRANSFERASE |
| SES1 | 9.90E-04 | 4.235 | TRNA SYNTHETASE, SERYL |
| Cluster 12 |  |  |  |
| WSC4 | 4.78E-08 | 7.119 | UNKNOWN |
| SKP1 | 1.29E-06 | 6.462 | KINETOCHORE PROTEIN |
| RAD5 | 1.46E-06 | -6.359 | DNA HELICASE |
| ENO2 | 1.46E-06 | 6.337 | ENOLASE II |
| PDB1 | 2.20E-06 | 6.229 | PYRUVATE DEHYDROGENASE |
| PPH21 | 4.47E-06 | 6.05 | PROTEIN PHOSPHATASE 2A |
| CMK2 | 4.47E-06 | 6.048 | CALMODULIN-DEPENDENT PROTEIN KINASE |
| SEC4 | 5.70E-06 | 5.982 | RAS-LIKE GTPASE; POST-GOLGI |
| WTM1 | 1.34E-05 | 5.78 | TRANSCRIPTION FACTOR |
| FBA1 | 1.34E-05 | 5.777 | ALDOLASE |
| MMS21 | 1.49E-05 | -5.742 | UNKNOWN |
| IDH1 | 2.64E-05 | 5.611 | ISOCITRATE DEHYDROGENASE |
| CBR1 | 5.20E-05 | 5.436 | CYTOCHROME B REDUCTASE |
| ERG9 | 5.20E-05 | 5.433 | SQUALENE SYNTHETASE |
| RHO1 | 5.39E-05 | 5.414 | GTP-BINDING PROTEIN, RHO FAMILY |
| MAP1 | 1.39E-04 | 5.202 | METHIONINE AMINOPEPTIDASE |
| HEM15 | 1.74E-04 | 5.143 | FERROCHELATASE (PROTOHEME FERROLYASE) |
| LSC1 | 4.17E-04 | 4.922 | SUCCINYL-COA LIGASE ALPHA SUBUNIT |
| VMA13 | 6.66E-04 | 4.798 | VACUOLAR H+-ATPASE V1 DOMAIN 54 KD SUBUN |
| MCK1 | 7.15E-04 | 4.773 | PROTEIN KINASE |
| RNP1 | 7.43E-04 | -4.756 | RIBONUCLEOPROTEIN |
| Cluster 13 |  |  |  |
| RSC1 | 7.46E-05 | -5.938 | CHROMATIN REMODELING COMPLEX SUBUNIT |
| SOK1 | 7.46E-05 | -5.827 | SUPPRESSOR OF A CAMP-DEPENDENT PROTEIN K |
| PDB1 | 1.07E-04 | 5.672 | PYRUVATE DEHYDROGENASE |
| Cluster 14 |  |  |  |
| SPE1 | 6.61E-11 | 8.23 | ORNITHINE DECARBOXYLASE |
| NUP42 | 1.84E-09 | 7.616 | NUCLEAR PORE PROTEIN |
| ROT1 | 1.78E-08 | 7.178 | UNKNOWN |
| EXG1 | 1.83E-08 | 7.141 | EXO-BETA-1,3-GLUCANASE |
| CDC28 | 2.24E-07 | 6.657 | CYCLIN DEPENDENT PROTEIN KINASE |
| RTT104 | 6.84E-07 | 6.424 | UNKNOWN; SIMILAR TO PIF1P AND OTHER HELI |
| CLB6 | 8.62E-07 | 6.338 | B-TYPE CYCLIN; S PHASE |
| RUB1 | 1.95E-06 | -6.163 | UBIQUITIN-LIKE PROTEIN |
| GRX4 | 4.99E-06 | 5.962 | UNKNOWN; SIMILAR TO TRX1P |
| SPR1 | 1.95E-05 | 5.618 | EXO-1,3-BETA-GLUCANASE |
| NUP120 | 2.02E-05 | 5.6 | NUCLEAR PORE PROTEIN |
| SPO12 | 3.04E-05 | 5.503 | UNKNOWN |
| KRE9 | 4.15E-05 | 5.418 | BETA-1,6-GLUCAN ASSEMBLY |
| MIF2 | 5.57E-05 | 5.337 | CENTROMERE PROTEIN |
| BDF2 | 2.06E-04 | -5.028 | UNKNOWN; SIMILAR TO MAMMALIAN GLYCOGEN |
| AYT1 | 2.41E-04 | 4.985 | TRANSACETYLASE (PUTATIVE) |
| SCD6 | 2.63E-04 | -4.958 | SUPPRESSOR OF CLATHRIN DEFICIENCY |
| MSL5 | 3.60E-04 | 4.865 | BRANCHPOINT BRIDGING PROTEIN (COMMITMENT |
| YND1 | 3.80E-04 | 4.839 | APYRASE (NDPASE/NTPASE) |
| CNB1 | 6.17E-04 | 4.701 | CALCINEURIN REGULATORY B SUBUNIT |
| CDC16 | 6.77E-04 | 4.672 | ANAPHASE-PROMOTING COMPLEX SUBUNIT |
| YPS7 | 6.77E-04 | 4.668 | GPI-ANCHORED ASPARTIC PROTEASE |
| OCH1 | 7.67E-04 | 4.633 | MEMBRANE-BOUND MANNOSYLTRANSFERASE |
| Cluster 15 |  |  |  |
| APG12 | 9.01E-07 | 6.648 | UNKNOWN |
| TOA1 | 2.34E-06 | 6.339 | TFIIA 32 KD SUBUNIT |
| CDC37 | 9.33E-06 | 5.992 | CHAPERONE |
| BUR6 | 1.35E-05 | 5.818 | GENERAL POL II REPRESSOR |
| SPP2 | 1.35E-05 | 5.818 | SPLICEOSOME-ASSOCIATED PROTEIN |
| PAF1 | 1.76E-05 | 5.745 | RNA POLYMERASE II-ASSOCIATED PROTEIN |
| YHC1 | 3.08E-05 | 5.614 | U1 SNRNP PROTEIN |
| SPT20 | 5.01E-05 | 5.481 | HISTONE ACETYLTRANSFERASE COMPLEX SUBUNI |
| LIP5 | 1.05E-04 | 5.279 | LIPOIC ACID SYNTHASE |
| CKI1 | 1.07E-04 | -5.247 | CHOLINE KINASE |
| SRB7 | 1.24E-04 | 5.202 | RNA POLYMERASE II MEDIATOR SUBUNIT |
| DBR1 | 1.24E-04 | -5.185 | DEBRANCHING ENZYME |
| AGA2 | 1.26E-04 | 5.162 | A-AGGLUTININ BINDING SUBUNIT |
| ADE8 | 1.26E-04 | 5.159 | PHOSPHORIBOSYLGLYCINAMIDE FORMYLTRANSFER |
| PPQ1 | 1.26E-04 | -5.15 | PROTEIN PHOSPHATASE |
| APN1 | 1.26E-04 | 5.145 | APURINIC/APYRIMIDINIC ENDONUCLEASE |
| GIM4 | 1.29E-04 | 5.133 | CHAPERONE; TUBULIN FOLDING |
| GPA1 | 1.83E-04 | 5.042 | ALPHA SUBUNIT OF G PROTEIN COUPLED TO MA |
| MAP2 | 1.97E-04 | 5.018 | METHIONINE AMINOPEPTIDASE 2 |
| MRP7 | 2.45E-04 | 4.944 | RIBOSOMAL PROTEIN, MITOCHONDRIAL LARGE S |
| HAT2 | 2.82E-04 | 4.906 | HISTONE ACETYLTRANSFERASE COMPLEX SUBUNI |
| HPC2 | 3.56E-04 | 4.847 | REGULATOR OF HISTONE TRANSCRIPTION |
| AIP2 | 4.25E-04 | 4.789 | ACTIN INTERACTING PROTEIN |
| SSO1 | 6.11E-04 | 4.664 | POST-GOLGI T-SNARE |
| BOS1 | 6.44E-04 | 4.647 | ER-TO-GOLGI V-SNARE |
| SNU23 | 7.61E-04 | 4.603 | U4/U6.U5 SNRNP PROTEIN |
| MRPL24 | 8.28E-04 | 4.578 | RIBOSOMAL PROTEIN, MITOCHONDRIAL L24 |
| APS1 | 9.32E-04 | 4.546 | AP-1 COMPLEX SUBUNIT |
| Cluster 16 |  |  |  |
| CKS1 | 3.19E-28 | -14.426 | PORTEIN KINASE REGULATOR |
| DIG2 | 1.58E-24 | 13.068 | NEGATIVE REGULATOR |
| MRPL9 | 1.13E-17 | 10.576 | RIBOSOMAL PROTEIN, MITOCHONDRIAL L9 |
| RAM2 | 3.68E-16 | -9.979 | PROTEIN FARNESYLTRANSFERASE, ALPHA SUBUN |
| RIB5 | 5.84E-16 | 9.886 | RIBOFLAVIN SYNTHASE, ALPHA CHAIN |
| VMA7 | 1.20E-15 | -9.753 | VACUOLAR H+-ATPASE V1 DOMAIN 14 KDA SUBU |
| ATR1 | 1.87E-15 | 9.667 | TRANSPORTER (PUTATIVE) |
| HEM2 | 1.15E-14 | -9.324 | PORPHOBILINOGEN SYNTHASE |
| NFU1 | 1.67E-14 | 9.253 | UNKNOWN; SIMILAR TO ANABAENA NITROGEN FI |
| MLC1 | 2.56E-14 | -9.175 | MYOSIN LIGHT CHAIN |
| RTG3 | 2.61E-14 | 9.161 | CIT2 REGULATOR |
| YCP4 | 3.21E-14 | 9.119 | UNKNOWN; SIMILAR TO S. POMBE BREFELDIN A |
| ASF2 | 5.17E-14 | -9.033 | ANTI-SILENCING PROTEIN |
| ECM40 | 7.03E-14 | 8.975 | ACETYLORNITHINE ACETYLTRANSFERASE |
| MSW1 | 1.46E-13 | 8.82 | TRNA SYNTHETASE, MITOCHONDRIAL, TRP |
| ESS1 | 1.90E-13 | -8.771 | PEPTIDYL-PROLYL CIS/TRANS ISOMERASE |
| TAH18 | 2.45E-13 | 8.717 | UNKNOWN; SIMILAR TO NAPDH-CYTOCHROME P45 |
| OPY1 | 2.45E-13 | 8.712 | UNKNOWN |
| PHO23 | 1.90E-12 | -8.357 | TRANSCRIPTIONAL REGULATOR OF PHO5 |
| KTR2 | 3.23E-12 | 8.259 | PUTATIVE MANNOSYLTRANSFERASE; TYPE 2 MEM |
| GOS1 | 5.50E-12 | -8.16 | GOLGI SNARE |
| EHT1 | 6.32E-12 | 8.133 | ALCOHOL ACYL TRANSFERASE (PUTATIVE) |
| VAN1 | 7.06E-12 | 8.109 | MANNOSYLTRANSFERASE |
| PHB1 | 1.07E-11 | 8.034 | UNKNOWN |
| YCK1 | 1.30E-11 | -7.998 | PROTEIN KINASE |
| YCK3 | 2.08E-11 | 7.91 | PLASMA MEMBRANE-BOUND CASEIN KINASE I |
| TRP4 | 4.16E-11 | 7.776 | ANTHRANILATE PHOSPHORIBOSYLTRANSFERASE |
| SPP381 | 4.44E-11 | 7.761 | U4/U6.U5 SNRNP PROTEIN |
| POL30 | 4.77E-11 | 7.745 | DNA POLYMERASE PROCESSIVITY FACTOR |
| SSO1 | 4.88E-11 | -7.738 | POST-GOLGI T-SNARE |
| STB1 | 5.42E-11 | -7.716 | BINDS SIN3P |
| IMG1 | 5.92E-11 | -7.698 | RIBOSOMAL PROTEIN, MITOCHONDRIAL |
| CAF16 | 9.70E-11 | 7.602 | ATP-BINDING CASSETTE (ABC) FAMILY |
| PDR16 | 1.03E-10 | 7.585 | UNKNOWN |
| MCM1 | 1.44E-10 | -7.524 | MULTIFUNCTIONAL REGULATOR |
| SAP185 | 1.70E-10 | -7.492 | SIT4P-ASSOCIATED PROTEIN |
| GRE2 | 5.84E-10 | 7.271 | UNKNOWN; INDUCED BY OSMOTIC STRESS |
| SFT2 | 7.25E-10 | 7.225 | SUPPRESSES SED5 TS MUTANTS |
| MRP17 | 8.32E-10 | 7.194 | RIBOSOMAL PROTEIN, MITOCHONDRIAL SMALL S |
| ATP17 | 8.32E-10 | 7.193 | ATP SYNTHASE SUBUNIT F |
| MEF1 | 1.03E-09 | 7.148 | TRANSLATION ELONGATION FACTOR G, MITOCHO |
| RPN10 | 1.07E-09 | -7.137 | 26S PROTEASOME SUBUNIT |
| CKB1 | 1.10E-09 | -7.123 | CASEIN KINASE II SUBUNIT |
| VPS45 | 1.26E-09 | 7.093 | MEMBRANE PROTEIN |
| NAP1 | 1.66E-09 | -7.033 | NUCLEOSOME ASSEMBLY PROTEIN |
| RFC3 | 2.13E-09 | -6.983 | REPLICATION FACTOR C 40 KD SUBUNIT |
| TRP5 | 2.29E-09 | 6.967 | TRYPTOPHAN SYNTHASE |
| ARG4 | 3.20E-09 | 6.894 | ARGININOSUCCINATE LYASE |
| BRN1 | 3.68E-09 | -6.865 | UNKNOWN; HOMOLOG OF HUMAN BRRN1 |
| VPS4 | 3.73E-09 | -6.858 | AAA ATPASE FAMILY |
| YSR3 | 3.90E-09 | 6.847 | DIHYDROSPHINGOSINE-1-PHOSPHATE PHOSPHATA |
| STU2 | 4.77E-09 | -6.808 | SPINDLE POLE BODY COMPONENT |
| HIS5 | 5.26E-09 | 6.786 | HISTIDINOL-PHOSPHATE AMINOTRANSFERASE |
| NAT2 | 5.29E-09 | -6.783 | N-ACETYLTRANSFERASE FOR N-TERMINAL METHI |
| TRS31 | 6.20E-09 | 6.749 | TRANSPORT PROTEIN PARTICLE (TRAPP) SUBUN |
| SPT8 | 8.15E-09 | -6.696 | HISTONE ACETYLTRANSFERASE COMPLEX SUBUNI |
| VTI1 | 8.27E-09 | 6.692 | CIS-GOLGI V-SNARE |
| TRX2 | 9.11E-09 | 6.672 | THIOREDOXIN II |
| PRE4 | 1.17E-08 | 6.616 | PROTEASOME SUBUNIT, B TYPE |
| UBC1 | 1.20E-08 | 6.611 | E2 UB.-CONJUGATING ENZYME |
| MRPL19 | 1.20E-08 | -6.609 | RIBOSOMAL PROTEIN, MITOCHONDRIAL L19 |
| TAL1 | 1.36E-08 | 6.582 | TRANSALDOLASE |
| SPT4 | 1.66E-08 | -6.54 | ELONGATION FACTOR |
| RAD54 | 2.59E-08 | -6.446 | DNA-DEPENDENT ATPASE |
| RPT3 | 2.66E-08 | 6.439 | 26S PROTEASOME SUBUNIT |
| YPR1 | 2.96E-08 | 6.418 | UNKNOWN; SIMILAR TO ALDO-KETO REDUCTASES |
| TPM1 | 3.18E-08 | -6.403 | TROPOMYOSIN |
| PET54 | 3.27E-08 | 6.396 | TRANSLATION ACTIVATOR OF COX3 |
| PDR17 | 3.38E-08 | 6.388 | UNKNOWN |
| YRF1-2 | 3.53E-08 | -6.379 | Y' HELICASE (SUBTELOMERICALLY-ENCODED) |
| NTA1 | 3.82E-08 | 6.362 | AMINO-TERMINAL AMIDASE |
| CLP1 | 4.05E-08 | 6.349 | CLEAVAGE/POLYADENYLATION FACTOR CF IA CO |
| ARH1 | 4.87E-08 | 6.305 | UNKNOWN; SIMILAR TO HUMAN ADRENODOXIN RE |
| ROT1 | 4.87E-08 | 6.305 | UNKNOWN |
| SGV1 | 5.12E-08 | -6.293 | PROTEIN KINASE |
| IRS4 | 5.45E-08 | -6.279 | UNKNOWN |
| LSM2 | 5.71E-08 | 6.268 | CORE SNRNP PROTEIN |
| NCP1 | 5.73E-08 | -6.264 | NADP-CYTOCHROME P450 REDUCTASE |
| LRS4 | 7.69E-08 | 6.201 | UNKNOWN |
| KRE1 | 9.68E-08 | -6.152 | BETA-1,6-GLUCAN ASSEMBLY |
| ADE16 | 9.78E-08 | -6.148 | 5-AMINOIMIDAZOLE-4-CARBOXAMIDE RIBONUCLE |
| IPP1 | 9.97E-08 | 6.143 | INORGANIC PYROPHOSPHATASE, CYTOPLASMIC |
| CDC16 | 1.04E-07 | 6.133 | ANAPHASE-PROMOTING COMPLEX SUBUNIT |
| YSH1 | 1.12E-07 | 6.116 | CLEAVAGE/POLYADENYLATION FACTOR CF II CO |
| CNB1 | 1.19E-07 | 6.101 | CALCINEURIN REGULATORY B SUBUNIT |
| TRS23 | 1.75E-07 | -6.022 | TRANSPORT PROTEIN PARTICLE (TRAPP) SUBUN |
| ECM31 | 1.76E-07 | 6.02 | UNKNOWN |
| SKY1 | 1.94E-07 | -5.998 | PROTEIN KINASE |
| RNA15 | 2.17E-07 | -5.973 | CLEAVAGE/POLYADENYLATION FACTOR CF I COM |
| CWH41 | 2.34E-07 | 5.955 | BETA-1,6-GLUCAN ASSEMBLY PROTEIN |
| OSM1 | 2.40E-07 | 5.945 | FUMARATE REDUCTASE |
| ADH1 | 2.80E-07 | -5.91 | ALCOHOL DEHYDROGENASE I |
| SAN1 | 2.88E-07 | -5.904 | (PUTATIVE) TRANSCRIPTIONAL REGULATOR |
| PAU2 | 2.88E-07 | -5.904 | UNKNOWN; SIMILAR TO MEMBERS OF THE SRP1P |
| HEM4 | 3.66E-07 | 5.854 | UROPORPHYRINOGEN III SYNTHASE |
| ARP2 | 3.67E-07 | 5.853 | ACTIN-RELATED PROTEIN |
| NMT1 | 4.04E-07 | 5.832 | N-MYRISTOYLTRANSFERASE |
| MRP21 | 4.21E-07 | 5.822 | RIBOSOMAL PROTEIN, MITOCHONDRIAL |
| SEC65 | 4.70E-07 | 5.796 | SIGNAL RECOGNITION PARTICLE SUBUNIT |
| Cluster 17 |  |  |  |
| PRS1 | 7.16E-25 | 13.277 | PHOSPHORIBOSYLPYROPHOSPHATE SYNTHETASE |
| GAR1 | 1.68E-19 | 11.286 | SNORNP PROTEIN |
| RPL19A | 1.89E-19 | 11.177 | RIBOSOMAL PROTEIN L19A |
| RPL8B | 1.89E-19 | 11.161 | RIBOSOMAL PROTEIN L8B |
| RPS9B | 8.10E-19 | 10.903 | RIBOSOMAL PROTEIN S9B |
| RPL42B | 2.35E-18 | 10.71 | RIBOSOMAL PROTEIN L42B |
| RPL23A | 4.83E-18 | 10.554 | RIBOSOMAL PROTEIN L23A |
| RPL16A | 5.20E-18 | 10.525 | RIBOSOMAL PROTEIN L16A |
| SUP45 | 5.58E-18 | 10.497 | TRANSLATION RELEASE FACTOR ERF1 SUBUNIT |
| RPS6B | 8.29E-18 | 10.421 | RIBOSOMAL PROTEIN S6B |
| DIM1 | 1.40E-17 | 10.326 | DIMETHYLADENOSINE TRANSFERASE |
| RPS2 | 3.30E-17 | 10.167 | RIBOSOMAL PROTEIN S2 |
| RPL19B | 3.95E-17 | 10.129 | RIBOSOMAL PROTEIN L19B |
| HIS6 | 6.05E-17 | 10.052 | PHOSPHORIBOSYL IMIDAZOLECARBOXAMIDE ISOM |
| RPL2B | 6.55E-17 | 10.03 | RIBOSOMAL PROTEIN L2B |
| RPL8A | 1.21E-16 | 9.925 | RIBOSOMAL PROTEIN L8A |
| RPL20B | 1.88E-16 | 9.847 | RIBOSOMAL PROTEIN L20B |
| RPL4B | 2.33E-16 | 9.8 | RIBOSOMAL PROTEIN L4B |
| RPS22A | 2.33E-16 | 9.791 | RIBOSOMAL PROTEIN S22A |
| RPS8A | 2.33E-16 | 9.789 | RIBOSOMAL PROTEIN S8 |
| STM1 | 2.97E-16 | 9.728 | UNKNOWN; SUPPRESSOR OF TOM1 AND POP2 MUT |
| RPL35B | 2.97E-16 | 9.724 | RIBOSOMAL PROTEIN L35B |
| YTM1 | 3.01E-16 | 9.711 | MICROTUBULE-ASSOCIATED PROTEIN |
| RPS18A | 3.23E-16 | 9.695 | RIBOSOMAL PROTEIN S18A |
| RPL32 | 3.40E-16 | 9.681 | RIBOSOMAL PROTEIN L23 |
| RPS26B | 4.39E-16 | 9.635 | RIBOSOMAL PROTEIN S26B |
| RPL10 | 5.20E-16 | 9.594 | RIBOSOMAL PROTEIN L10 |
| RPS29A | 5.62E-16 | 9.573 | RIBOSOMAL PROTEIN S29A |
| NIP7 | 6.27E-16 | 9.547 | UNKNOWN |
| RPL14B | 6.59E-16 | 9.535 | RIBOSOMAL PROTEIN L14B |
| RPS23A | 6.76E-16 | 9.527 | RIBOSOMAL PROTEIN S23A |
| RPL7B | 8.46E-16 | 9.483 | RIBOSOMAL PROTEIN L7B |
| RPL30 | 8.54E-16 | 9.478 | RIBOSOMAL PROTEIN L30 |
| RPS21A | 1.09E-15 | 9.435 | RIBOSOMAL PROTEIN S21A |
| RPA12 | 1.22E-15 | 9.414 | RNA POLYMERASE I SUBUNIT |
| RPL6A | 1.53E-15 | 9.37 | RIBOSOMAL PROTEIN L6A |
| RPL21A | 1.57E-15 | 9.363 | RIBOSOMAL PROTEIN L21A |
| RPS18B | 1.57E-15 | 9.36 | RIBOSOMAL PROTEIN S18B |
| RPL13A | 1.75E-15 | 9.339 | RIBOSOMAL PROTEIN L13A |
| TIF5 | 2.08E-15 | 9.308 | TRANSLATION INITIATION FACTOR EIF5 |
| HAS1 | 2.08E-15 | 9.305 | RNA HELICASE |
| CAF20 | 2.64E-15 | 9.264 | MRNA CAP-BINDING PROTEIN (EIF4F) 20K SUB |
| RPL40A | 2.88E-15 | 9.247 | RIBOSOMAL PROTEIN L40A |
| RPL34A | 2.88E-15 | 9.244 | RIBOSOMAL PROTEIN L34A |
| PPT1 | 2.98E-15 | 9.236 | PROTEIN PHOSPHATASE |
| RPL26B | 4.06E-15 | 9.183 | RIBOSOMAL PROTEIN L26B |
| RPS25A | 4.63E-15 | 9.16 | RIBOSOMAL PROTEIN S25A |
| RPL20A | 6.18E-15 | 9.108 | RIBOSOMAL PROTEIN L20A |
| MAK11 | 6.18E-15 | 9.108 | UNKNOWN; ESSENTIAL GENE |
| RPL7A | 7.67E-15 | 9.07 | RIBOSOMAL PROTEIN L7A |
| RPL37A | 8.89E-15 | 9.044 | RIBOSOMAL PROTEIN L37A |
| RPS17A | 9.18E-15 | 9.036 | RIBOSOMAL PROTEIN S17A |
| RPL40B | 9.30E-15 | 9.031 | RIBOSOMAL PROTEIN L40B |
| TIF6 | 9.79E-15 | 9.014 | UNKNOWN; SIMILAR TO HUMAN TRANSLATION IN |
| MRT4 | 9.79E-15 | 9.013 | UNKNOWN |
| RPS23B | 9.79E-15 | 9.011 | RIBOSOMAL PROTEIN S23B |
| GIS2 | 1.02E-14 | 9.001 | UNKNOWN; GIG3 SUPPRESSOR |
| RPL13B | 1.35E-14 | 8.954 | RIBOSOMAL PROTEIN L13B |
| NCL1 | 1.86E-14 | 8.898 | UNKNOWN; SIMILAR TO HUMAN PROLIFERATING |
| RPS31 | 1.86E-14 | 8.897 | RIBOSOMAL PROTEIN S31 |
| RPS1B | 1.86E-14 | 8.895 | RIBOSOMAL PROTEIN S1B |
| RPL37B | 1.92E-14 | 8.887 | RIBOSOMAL PROTEIN L37B |
| RPL4A | 1.92E-14 | 8.886 | RIBOSOMAL PROTEIN L4A |
| URA7 | 2.01E-14 | 8.876 | CTP SYNTHASE 1 |
| RPS13 | 2.05E-14 | 8.871 | RIBOSOMAL PROTEIN S13 |
| NIP1 | 2.28E-14 | 8.852 | UNKNOWN, SIMILAR TO NSR1 |
| RPL14A | 2.62E-14 | 8.827 | RIBOSOMAL PROTEIN L14A |
| RPL39 | 3.04E-14 | 8.8 | RIBOSOMAL PROTEIN L39 |
| RNC1 | 3.28E-14 | 8.784 | ENDO-EXONUCLEASE |
| ARO4 | 3.40E-14 | 8.774 | 2-DEHYDRO-3-DEOXYPHOSPHOHEPTONATE ALDOLA |
| RPA49 | 3.68E-14 | 8.76 | RNA POLYMERASE I 46 KD SUBUNIT |
| RPL43A | 3.79E-14 | 8.751 | RIBOSOMAL PROTEIN L34A |
| HMT1 | 3.88E-14 | 8.745 | ARGININE METHYLTRANSFERASE |
| IMP4 | 4.40E-14 | 8.723 | U3 SNORNP PROTEIN |
| RPL27B | 4.42E-14 | 8.72 | RIBOSOMAL PROTEIN L27B |
| RPL9A | 4.63E-14 | 8.71 | RIBOSOMAL PROTEIN L9A |
| MPP10 | 4.76E-14 | 8.703 | U3 SNORNP PROTEIN |
| RPS27A | 6.19E-14 | 8.655 | RIBOSOMAL PROTEIN S27A |
| RPS6A | 6.23E-14 | 8.651 | RIBOSOMAL PROTEIN S6A |
| RPL33B | 6.23E-14 | 8.649 | RIBOSOMAL PROTEIN L33B |
| RPL23B | 6.77E-14 | 8.632 | RIBOSOMAL PROTEIN L23B |
| RPL2A | 6.80E-14 | 8.629 | RIBOSOMAL PROTEIN L2A |
| PAB1 | 6.98E-14 | 8.623 | CLEAVAGE/POLYADENYLATION FACTOR CF IA CO |
| CBF5 | 6.98E-14 | 8.621 | CENTROMERIC MICROTUBULE BINDING PROTEIN |
| HTA2 | 9.24E-14 | 8.571 | HISTONE H2A |
| KRR1 | 9.33E-14 | 8.568 | ESSENTIAL |
| RPS15 | 9.36E-14 | 8.566 | RIBOSOMAL PROTEIN S15 |
| RPL11A | 9.92E-14 | 8.553 | RIBOSOMAL PROTEIN L11A |
| SNU13 | 9.92E-14 | 8.552 | U4/U6.U5 SNRNP PROTEIN |
| RPS30B | 9.92E-14 | 8.552 | RIBOSOMAL PROTEIN S30B |
| RPL42A | 9.92E-14 | 8.549 | RIBOSOMAL PROTEIN L42A |
| NOP58 | 1.46E-13 | 8.485 | NUCLEOLAR PROTEIN |
| NMD3 | 1.51E-13 | 8.477 | NAM7P/UPF1P-INTERACTING PROTEIN |
| RPL36A | 1.59E-13 | 8.466 | RIBOSOMAL PROTEIN L36A |
| PWP1 | 1.73E-13 | 8.451 | UNKNOWN; SIMILAR TO BETA-TRANSDUCIN SUPE |
| RPB8 | 1.76E-13 | 8.446 | SHARED SUBUNIT OF RNA POLYMERASE I,II, A |
| NOP1 | 1.87E-13 | 8.435 | FIBRILLARIN HOMOLOG |
| RPL33A | 2.29E-13 | 8.398 | RIBOSOMAL PROTEIN L33A |
| RPL11B | 2.87E-13 | 8.355 | RIBOSOMAL PROTEIN L11B |
| Cluster 18 |  |  |  |
| MFA2 | 6.56E-10 | 7.784 | A-FACTOR PRECURSOR |
| ATP4 | 1.54E-08 | -7.111 | ATPASE; F0-ATP SYNTHASE SUBUNIT 4 |
| GOG5 | 6.15E-08 | 6.833 | GOLGI GDP-MANNOSE TRANSPORTER |
| TAF47 | 7.42E-08 | 6.753 | COMPONENT OF TAF(II) COMPLEX |
| YFH1 | 1.81E-07 | 6.534 | FRATAXIN HOMOLOG |
| HOM6 | 3.93E-07 | 6.373 | HOMOSERINE DEHYDROGENASE |
| HXT17 | 8.25E-07 | 6.204 | HEXOSE PERMEASE |
| GAL1 | 9.81E-07 | -6.135 | GALACTOKINASE |
| RMS1 | 9.98E-07 | 6.121 | (PUTATIVE) TRANSCRIPTIONAL REGULATOR |
| POR2 | 1.89E-06 | 5.959 | PORIN, ANION CHANNEL |
| PMP2 | 1.99E-06 | 5.94 | REGULATES PLASMA MEMBRANE H+-ATPASE |
| ERG10 | 2.23E-06 | 5.91 | ACETOACETYL COA THIOLASE |
| GAL2 | 3.88E-06 | -5.791 | GLUCOSE AND GALACTOSE PERMEASE |
| RPS28B | 5.89E-06 | 5.699 | RIBOSOMAL PROTEIN S28B |
| ALA1 | 5.95E-06 | 5.685 | TRNA SYNTHETASE, ALANYL |
| QRI5 | 5.95E-06 | -5.683 | UNKNOWN |
| RCE1 | 7.24E-06 | 5.636 | PROTEASE, ACTS ON RAS AND A-FACTOR C-TER |
| MRPL44 | 7.61E-06 | -5.616 | RIBOSOMAL PROTEIN, MITOCHONDRIAL L44 |
| AUR1 | 7.61E-06 | 5.612 | PHOSPHATIDYLINOSITOL:CERAMIDE PHOSPHOINO |
| MAL32 | 9.27E-06 | -5.566 | ALPHA-GLUCOSIDASE |
| ATP3 | 1.25E-05 | -5.497 | MITOCHONDRIAL F1F0 ATP SYNTHASE SUBUNIT |
| LAC1 | 1.28E-05 | 5.486 | UNKNOWN; SIMILAR TO LAG1P, HAS 6 POTENTI |
| ANB1 | 1.36E-05 | 5.466 | TRANSLATION INITIATION FACTOR EIF5A |
| GAL7 | 1.36E-05 | -5.463 | GAL-1-PHOSPHATE URIDYL TRANSFERASE |
| IDI1 | 1.81E-05 | 5.392 | ISOPENTENYL-DIPHOSPHATE DELTA-ISOMERASE |
| COX12 | 1.85E-05 | -5.374 | CYTOCHROME-C OXIDASE, SUBUNIT VIB |
| TEF4 | 1.85E-05 | 5.368 | TRANSLATION ELONGATION FACTOR EF-1GAMMA |
| SEN15 | 1.85E-05 | 5.366 | SPLICING ENDONUCLEASE SUBUNIT |
| SSH4 | 3.15E-05 | -5.234 | SUPPRESSES SHR3 MUTATION |
| RPA12 | 3.58E-05 | 5.189 | RNA POLYMERASE I SUBUNIT |
| GAL10 | 3.83E-05 | -5.17 | UDP-GLUCOSE 4-EPIMERASE |
| KGD2 | 3.91E-05 | -5.162 | 2-OXOGLUTARATE DEHYDROGENASE |
| YMR31 | 3.91E-05 | -5.158 | RIBOSOMAL PROTEIN, MITOCHONDRIAL |
| FEN1 | 3.92E-05 | 5.153 | BETA-1,3-GLUCAN SYNTHASE SUBUNIT |
| RPS13 | 3.92E-05 | 5.149 | RIBOSOMAL PROTEIN S13 |
| GPM1 | 4.57E-05 | 5.108 | PHOSPHOGLYCERATE MUTASE |
| SAR1 | 4.97E-05 | 5.087 | GTP-BINDING PROTEIN OF THE ARF FAMILY |
| RPS12 | 5.18E-05 | 5.074 | RIBOSOMAL PROTEIN S12 |
| STB6 | 5.30E-05 | 5.065 | BINDS SIN3P |
| ENO2 | 5.62E-05 | 5.046 | ENOLASE II |
| COX13 | 5.67E-05 | -5.039 | CYTOCHROME-C OXIDASE SUBUNIT VIA |
| COX7 | 6.44E-05 | -5.008 | CYTOCHROME-C OXIDASE, SUBUNIT VII |
| SNU13 | 7.56E-05 | 4.962 | U4/U6.U5 SNRNP PROTEIN |
| ATP7 | 7.56E-05 | -4.962 | F1F0-ATPASE COMPLEX, FO D SUBUNIT |
| MER1 | 9.06E-05 | 4.917 | RNA BINDING PROTEIN |
| MDH1 | 9.06E-05 | -4.916 | MALATE DEHYDROGENASE |
| UBP16 | 1.09E-04 | -4.869 | PUTATIVE DEUBIQUITINATING ENZYME |
| CYB2 | 1.14E-04 | -4.855 | CYTOCHROME B2 |
| CUP5 | 1.30E-04 | 4.82 | VACUOLAR ATP SYNTHASE SUBUNIT |
| NDI1 | 1.33E-04 | -4.811 | NADH-UBIQUINONE-6 OXIDOREDUCTASE |
| SGE1 | 1.36E-04 | 4.803 | TRANSPORTER, MAJOR FACILITATOR SUPERFAMI |
| DYS1 | 1.48E-04 | 4.78 | DEOXYHYPUSINE SYNTHASE |
| RPL14B | 1.54E-04 | 4.768 | RIBOSOMAL PROTEIN L14B |
| CHS7 | 1.55E-04 | 4.762 | UNKNOWN |
| PIS1 | 1.55E-04 | 4.757 | PHOSPHATIDYLINOSITOL SYNTHASE |
| TGL2 | 1.55E-04 | 4.756 | TRIACYLGLYCEROL LIPASE |
| UBC11 | 1.66E-04 | 4.734 | HOMOLOG OF UBIQUITIN CARRIER PROTEIN E2- |
| ERG2 | 1.99E-04 | 4.688 | C-8 STEROL ISOMERASE |
| RPL33B | 2.21E-04 | 4.658 | RIBOSOMAL PROTEIN L33B |
| TDH3 | 2.24E-04 | 4.648 | GLYCERALDEHYDE-3-PHOSPHATE DEHYDROGENASE |
| NYV1 | 2.28E-04 | -4.638 | VACUOLAR V-SNARE |
| MSK1 | 2.29E-04 | -4.635 | TRNA SYNTHETASE, MITOCHONDRIAL, LYSYL |
| MCR1 | 2.29E-04 | -4.632 | CYTOCHROME-B5 REDUCTASE |
| MEI5 | 2.39E-04 | 4.614 | UNKNOWN |
| ERG24 | 2.39E-04 | 4.612 | C-14 STEROL REDUCTASE |
| ISU1 | 2.49E-04 | -4.593 | UNKNOWN; SIMILAR TO IRON-SULFUR CLUSTER |
| RPL18A | 2.58E-04 | 4.583 | RIBOSOMAL PROTEIN L18A |
| HES1 | 2.58E-04 | 4.581 | UNKNOWN |
| DNM1 | 2.66E-04 | -4.572 | DYNAMIN-RELATED PROTEIN |
| RPL26A | 2.67E-04 | 4.568 | RIBOSOMAL PROTEIN L26A |
| COX8 | 2.70E-04 | -4.559 | CYTOCHROME-C OXIDASE CHAIN VIII |
| TRM3 | 2.70E-04 | 4.559 | TRNA ROBOSE METHYLASE |
| QCR2 | 3.05E-04 | -4.525 | UBIQUINOL--CYTOCHROME-C REDUCTASE 40 KD |
| ATP5 | 3.23E-04 | -4.5 | F1F0-ATPASE SUBUNIT |
| NAM2 | 3.59E-04 | -4.472 | TRNA SYNTHETASE, MITOCHONDRIAL, LEUCYL |
| ALG9 | 3.84E-04 | 4.449 | MANNOSYLTRANSFERASE |
| NDJ1 | 3.91E-04 | 4.441 | UNKNOWN |
| ECM13 | 3.93E-04 | -4.439 | UNKNOWN |
| PRP12 | 3.97E-04 | -4.434 | MITOCHONDRIAL INNER MEMBRANE PROTEIN |
| PMT4 | 4.06E-04 | 4.426 | DOLICHYL PHOSPHATE-D-MANNOSE:PROTEIN O-D |
| CPR7 | 4.12E-04 | 4.422 | PEPTIDYL-PROLYL CIS/TRANS ISOMERASE |
| RPL23B | 4.28E-04 | 4.408 | RIBOSOMAL PROTEIN L23B |
| SVL3 | 4.56E-04 | 4.387 | UNKNOWN; STYRYL DYE VACUOLAR LOCALIZATIO |
| TIF2 | 5.05E-04 | 4.357 | TRANSLATION INITIATION FACTOR EIF4A |
| PDE2 | 5.05E-04 | 4.355 | HIGH AFFINITY 3',5'-CAMP PHOSPHODIESTERA |
| THR1 | 5.30E-04 | 4.342 | HOMOSERINE KINASE |
| SUN4 | 5.30E-04 | 4.341 | UNKNOWN |
| STB3 | 5.30E-04 | -4.338 | BINDS SIN3P |
| SDH4 | 5.30E-04 | -4.334 | SUCCINATE DEHYDROGENASE ANCHOR SUBUNIT |
| CEF1 | 5.30E-04 | -4.332 | UNKNOWN; SIMILAR TO S. POMBE CDC5+ |
| RPL16B | 5.30E-04 | 4.331 | RIBOSOMAL PROTEIN L16B |
| PKR1 | 5.30E-04 | 4.331 | UNKNOWN |
| RPS27B | 5.76E-04 | 4.305 | RIBOSOMAL PROTEIN S27B |
| PET117 | 5.79E-04 | -4.3 | CYTOCHROME C OXIDASE ASSEMBLY FACTOR |
| RPL23A | 5.79E-04 | 4.298 | RIBOSOMAL PROTEIN L23A |
| RPP1A | 5.88E-04 | 4.291 | RIBOSOMAL PROTEIN P1A, ACIDIC |
| ECM19 | 5.88E-04 | -4.29 | UNKNOWN |
| REV1 | 5.92E-04 | 4.287 | DEOXYCYTIDYL TRANSFERASE |
| SIP2 | 5.96E-04 | -4.283 | COMPONENT OFSNF1 PROTEIN COMPLEX |
| Cluster 19 |  |  |  |
| PRP9 | 1.99E-12 | 8.796 | U2 SNRNP ACTIVATION |
| NRF1 | 1.39E-10 | 7.942 | NEGATIVE REGULATOR OF CDC42P |
| GPR1 | 1.39E-10 | 7.938 | G-PROTEIN COUPLED RECEPTOR, COUPLED TO G |
| PRP8 | 7.95E-10 | 7.581 | U4/U6, U5 SNRNP PROTEIN |
| SPL2 | 8.43E-10 | 7.548 | PROTEIN KINASE INHIBITOR |
| RAT1 | 3.52E-09 | 7.246 | EXONUCLEASE II |
| CDC2 | 3.52E-09 | 7.231 | DNA POLYMERASE DELTA CATALYTIC 125 KD SU |
| SIT1 | 3.52E-09 | 7.226 | FERRIOXAMINE B PERMEASE |
| NAN1 | 3.52E-09 | 7.212 | UNKNOWN; NUCLEOLAR PROTEIN |
| IPL1 | 5.37E-09 | 7.123 | PROTEIN KINASE |
| MYO1 | 9.79E-09 | 7.003 | MYOSIN HEAVY CHAIN |
| FAS2 | 1.42E-08 | 6.914 | FATTY-ACYL-COA SYNTHASE, ALPHA SUBUNIT |
| IKI3 | 6.41E-08 | 6.626 | UNKNOWN |
| ATF2 | 9.84E-07 | 6.06 | ALCOHOL ACETYLTRANSFERASE |
| PHO81 | 1.07E-06 | 6.026 | PHO85P KINASE INHIBITOR |
| MNN1 | 1.10E-06 | 6.014 | ALPHA-1,3-MANNOSYLTRANSFERASE |
| ECM1 | 3.17E-06 | 5.776 | UNKNOWN |
| ECM16 | 3.17E-06 | 5.775 | UNKNOWN |
| PHO84 | 3.69E-06 | 5.737 | INORGANIC PHOSPHATE PERMEASE |
| ASK10 | 4.08E-06 | 5.711 | ENHANCER OF SKN7-DEPENDENT TRANSCRIPTION |
| POL1 | 4.15E-06 | 5.695 | POLYMERASE ALPHA 180 KD SUBUNIT |
| BET5 | 4.20E-06 | 5.686 | UNKNOWN; SUPPRESSES BET3 MUTATION |
| BIO2 | 4.69E-06 | 5.656 | BIOTIN SYNTHETASE |
| ERG3 | 5.35E-06 | 5.604 | C-5 STEROL DESATURASE |
| RPA190 | 5.58E-06 | 5.587 | RNA POLYMERASE I 190 KD SUBUNIT |
| MTR4 | 6.14E-06 | 5.562 | RNA HELICASE |
| MMP1 | 6.75E-06 | 5.535 | HIGH AFFINITY S-METHYLMETHIONINE PERMEAS |
| MSR1 | 7.80E-06 | -5.499 | TRNA SYNTHETASE, ARGINYL |
| SCC2 | 7.86E-06 | 5.493 | UNKNOWN; BINDS CHROMOSOMES |
| MCM3 | 1.06E-05 | 5.41 | MCM INITIATOR COMPLEX |
| KAP123 | 1.09E-05 | 5.4 | BETA-KARYOPHERIN |
| GEA1 | 1.26E-05 | 5.365 | GDP/GTP EXCHANGE FACTOR FOR ARF |
| CDC15 | 1.36E-05 | 5.346 | PROTEIN KINASE |
| KIN2 | 1.44E-05 | 5.327 | PROTEIN KINASE |
| TIS11 | 1.44E-05 | 5.326 | UNKNOWN |
| BEM4 | 1.70E-05 | 5.28 | INTERACTS WITH RHO-TYPE GTPASES |
| ISW1 | 2.03E-05 | 5.226 | UNKNOWN; SIMILAR TO DROSOPHILA NUCLEOSOM |
| AXL1 | 2.07E-05 | 5.218 | UNKNOWN; INSULIN-DEGRADING ENZYME HOMOLO |
| SKT5 | 2.14E-05 | 5.207 | CHITIN SYNTHASE REGULATOR |
| SWH1 | 2.15E-05 | 5.201 | UNKNOWN; SIMILAR TO MAMMALIAN OXYSTEROL- |
| DRS1 | 2.38E-05 | 5.176 | RNA HELICASE |
| SMX3 | 2.50E-05 | -5.162 | CORE SNRNP PROTEIN |
| CHS2 | 2.54E-05 | 5.155 | CHITIN SYNTHASE II |
| ENP1 | 2.61E-05 | 5.143 | PUTATIVE OLIGOSACCHARYLTRANSFERASE COMPL |
| RAP1 | 2.61E-05 | 5.139 | TRANSCRIPTIONAL REPRESSOR AND ACTIVATOR |
| GPD1 | 3.43E-05 | -5.065 | GLYCEROL-3-PHOSPHATE DEHYDROGENAS |
| CTF4 | 3.87E-05 | 5.032 | POLYMERASE ALPHA BINDING PROTEIN |
| CYS3 | 3.87E-05 | 5.03 | CYSTATHIONINE GAMMA-LYASE |
| SPA2 | 3.98E-05 | 5.018 | CORTICAL ACTIN PATCH COMPONENT |
| ZAP1 | 4.00E-05 | 5.013 | TRANSCRIPTION FACTOR |
| ACC1 | 4.03E-05 | 5.007 | ACETYL-COA CARBOXYLASE |
| SAP185 | 4.03E-05 | 5.007 | SIT4P-ASSOCIATED PROTEIN |
| SNF2 | 4.17E-05 | 4.994 | COMPONENT OF SWI/SNF GLOBAL ACTIVATOR CO |
| RAD9 | 4.54E-05 | 4.968 | UNKNOWN |
| SGD1 | 4.66E-05 | 4.953 | HIGH OSMOLARITY PATHWAY |
| PMR1 | 4.66E-05 | 4.945 | CA(2+) ATPASE |
| TOP2 | 5.13E-05 | 4.918 | DNA TOPOISOMERASE II |
| UBP13 | 5.13E-05 | 4.917 | UBIQUITIN CARBOXYL-TERMINAL HYDROLASE |
| NCR1 | 5.80E-05 | 4.879 | UNKNOWN |
| MSP1 | 5.80E-05 | -4.877 | AAA-ATPASE |
| MXR1 | 5.80E-05 | 4.877 | PEPTIDE-METHIONINE SULFOXIDE REDUCTASE |
| PWP2 | 6.44E-05 | 4.848 | UNKNOWN |
| MED1 | 6.63E-05 | 4.839 | RNA POLYMERASE II MEDIATOR SUBUNIT |
| NRD1 | 6.89E-05 | 4.825 | ELONGATION; ALSO MRNA ABUNDANCE |
| THR4 | 8.50E-05 | 4.768 | THREONINE SYNTHASE |
| FPR4 | 8.62E-05 | 4.763 | SIMILAR TO PEPTIDYL-PROLYL CIS-TRANS ISO |
| POP1 | 9.04E-05 | 4.747 | RNASE P AND RNASE MRP SUBUNIT |
| MIS1 | 9.04E-05 | 4.746 | C1-TETRAHYDROFOLATE SYNTHASE |
| NAB2 | 9.10E-05 | 4.743 | POLY(A)+RNA BINDING PROTEIN |
| TRM3 | 9.10E-05 | 4.741 | TRNA ROBOSE METHYLASE |
| MAK16 | 9.13E-05 | 4.737 | UNKNOWN; ESSENTIAL GENE |
| URA7 | 9.23E-05 | 4.733 | CTP SYNTHASE 1 |
| PUN1 | 9.49E-05 | -4.725 | UNKNOWN |
| AFG2 | 1.01E-04 | 4.706 | (PUTATIVE) AAA ATPASE |
| NRG2 | 1.01E-04 | -4.705 | UNKNOWN |
| ORC1 | 1.07E-04 | 4.686 | ORIGIN RECOGNITION COMPLEX 104 KD SUBUNI |
| EFD1 | 1.07E-04 | 4.685 | UNKNOWN |
| DBP10 | 1.15E-04 | 4.668 | UNKNOWN; SIMILAR TO RNA HELICASES |
| UTR2 | 1.15E-04 | 4.663 | UNKNOWN |
| AFR1 | 1.18E-04 | -4.655 | CYTOSKELETAL PROTEIN, SIMILAR TO ARRESTI |
| RFA1 | 1.22E-04 | 4.642 | REPLICATION FACTOR A, 69 KD SUBUNIT |
| IQG1 | 1.24E-04 | 4.637 | IQGAP HOMOLOG |
| PHO12 | 1.28E-04 | 4.628 | SECRETED ACID PHOSPHATASE |
| ESC4 | 1.29E-04 | 4.625 | UNKNOWN |
| SSU72 | 1.33E-04 | 4.617 | NUCLEAR PROTEIN |
| SPT5 | 1.34E-04 | 4.613 | ELONGATION FACTOR |
| DPH5 | 1.36E-04 | 4.609 | DIPHTHAMIDE METHYLTRANSFERASE |
| PCM1 | 1.43E-04 | 4.594 | PHOSPHOACETYLGLUCOSAMINE MUTASE |
| ZDS1 | 1.56E-04 | 4.57 | PERIPHERAL PLASMA MEMBRANE PROTEIN |
| GPM2 | 1.57E-04 | -4.567 | PHOSPHOGLYCERATE MUTASE |
| PIF1 | 1.57E-04 | 4.566 | DNA HELICASE |
| REC104 | 1.66E-04 | -4.549 | DS BREAK FORMATION COMPLEX SUBUNIT |
| NUP145 | 1.66E-04 | 4.549 | NUCLEAR PORE PROTEIN |
| PPZ1 | 1.70E-04 | 4.541 | SER/THR PHOSPHATASE |
| DFG16 | 1.81E-04 | 4.526 | UNKNOWN |
| RPO31 | 1.97E-04 | 4.504 | RNA POLYMERASE III 160 KD SUBUNIT |
| SEN1 | 1.97E-04 | 4.502 | RNA HELICASE, PUTATIVE |
| RRP5 | 1.98E-04 | 4.501 | UNKNOWN; REQUIRED FOR PRE-RRNA CLEAVAGE |
| NAB3 | 2.01E-04 | 4.495 | NUCLEAR POLYADENYLATED RNA-BINDING PROTE |
| Cluster 20 |  |  |  |
| --- |  |  |  |
| Cluster 21 |  |  |  |
| --- | 2.76E-13 | 9.243 | MAJOR FACILITATOR SUPERFAMILY |
| POP7 | 1.01E-11 | -8.479 | RNASE P AND RNASE MRP SUBUNIT |
| CGR1 | 2.73E-10 | 7.808 | UNKNOWN; SIMILAR TO HUMAN CHROMATIN ASSE |
| RRP7 | 6.38E-10 | 7.612 | UNKNOWN |
| AGP3 | 1.40E-08 | -7.028 | GENERAL AMINO ACID PERMEASE |
| DOM34 | 1.62E-08 | 6.943 | UNKNOWN |
| PNT1 | 3.55E-08 | 6.769 | UNKNOWN |
| YEA4 | 3.89E-08 | -6.735 | UNKNOWN; SIMILAR TO GOG5, A GENE INVOLVE |
| VCX1 | 8.50E-08 | 6.58 | VACUOLAR H+/CA(2+) EXCHANGER |
| PHO12 | 8.74E-08 | 6.565 | SECRETED ACID PHOSPHATASE |
| ZMS1 | 1.08E-07 | -6.509 | UNKNOWN; SIMILAR TO ADR1P AND YML081P |
| COS12 | 3.39E-07 | 6.253 | UNKNOWN; SIMILAR TO SUBTELOMERICALLY-ENC |
| PHO11 | 4.52E-07 | 6.176 | SECRETED ACID PHOSPHATASE |
| FRE4 | 5.50E-07 | 6.132 | UNKNOWN; SIMILAR TO FERRIC REDUCTASES FR |
| RRP45 | 2.84E-06 | 5.743 | 3'->5' EXORIBONUCLEASE |
| MAK3 | 2.92E-06 | 5.733 | L-A VIRUS GAG PROTEIN N-ACETYLTRANSFERAS |
| SEN2 | 3.07E-06 | 5.718 | SPLICING ENDONUCLEASE SUBUNIT |
| AGA1 | 3.52E-06 | 5.686 | A-AGGLUTININ ANCHOR SUBUNIT |
| RPI1 | 3.73E-06 | -5.665 | NEGATIVE REGULATOR OF RAS-CAMP PATHWAY |
| GIS2 | 4.13E-06 | 5.64 | UNKNOWN; GIG3 SUPPRESSOR |
| SIT1 | 4.21E-06 | 5.632 | FERRIOXAMINE B PERMEASE |
| CTR2 | 9.43E-06 | 5.446 | COPPER TRANSPORTER |
| VPS30 | 1.21E-05 | -5.389 | UNKNOWN? |
| RPA34 | 1.44E-05 | 5.349 | RNA POLYMERASE I SUBUNIT |
| MRPL31 | 1.63E-05 | 5.319 | RIBOSOMAL PROTEIN, MITOCHONDRIAL L31 |
| SED5 | 1.63E-05 | 5.316 | ER-TO-GOLGI T-SNARE |
| FRE5 | 1.82E-05 | 5.281 | UNKNOWN; SIMILAR TO FRE2P |
| RPL41A | 1.83E-05 | 5.277 | RIBOSOMAL PROTEIN L41A |
| YPT6 | 2.20E-05 | 5.221 | GTP-BINDING PROTEIN, RAB FAMILY |
| TIS11 | 3.09E-05 | 5.13 | UNKNOWN |
| REF2 | 3.28E-05 | 5.111 | UNKNOWN |
| HMT1 | 3.39E-05 | 5.099 | ARGININE METHYLTRANSFERASE |
| MRS11 | 3.45E-05 | 5.092 | COMPONENT OF MITOCHONDRIAL IMPORT MACHIN |
| SDC25 | 4.64E-05 | 5.008 | GDP/GTP EXCHANGE FACTOR FOR RAS |
| MRPL32 | 5.06E-05 | 4.984 | RIBOSOMAL PROTEIN, MITOCHONDRIAL L32 |
| MSS116 | 5.15E-05 | 4.975 | RNA HELICASE |
| SEC72 | 5.35E-05 | 4.962 | ER PROTEIN TRANSLOCATION SUBCOMPLEX SUBU |
| MMS2 | 5.39E-05 | -4.957 | UNKNOWN |
| SAS5 | 5.68E-05 | 4.942 | UNKNOWN |
| SRP21 | 5.68E-05 | 4.941 | SIGNAL RECOGNITION PARTICLE SUBUNIT |
| RSR1 | 6.04E-05 | 4.919 | GTP-BINDING PROTEIN, RAS SUPERFAMILY |
| ULA1 | 6.04E-05 | -4.919 | RUB1P ACTIVATING PROTEIN |
| GLC7 | 6.65E-05 | 4.885 | PROTEIN PHOSPHATASE |
| KNH1 | 6.73E-05 | -4.88 | KRE9P HOMOLOG |
| ARP9 | 6.75E-05 | 4.877 | ACTIN-RELATED PROTEIN |
| TYE7 | 7.05E-05 | 4.862 | BASIC H-L-H TRANSCRIPTION FACTOR |
| GIM3 | 7.55E-05 | 4.842 | PREFOLDIN SUBUNIT 4 |
| IMP3 | 7.69E-05 | 4.836 | U3 SNORNP PROTEIN |
| ARG2 | 8.69E-05 | -4.804 | ACETYLGLUTAMATE SYNTHASE |
| COT1 | 1.03E-04 | 4.755 | MITOCHONDRIAL MEMBRANE PROTEIN |
| RNH1 | 1.28E-04 | 4.695 | RIBONUCLEASE H |
| RRS1 | 1.31E-04 | 4.687 | UNKNOWN |
| STE4 | 1.31E-04 | 4.685 | BETA SUBUNIT OF G PROTEIN |
| RPC31 | 1.35E-04 | 4.677 | RNA POLYMERASE III 31 KD SUBUNIT |
| BAT2 | 1.41E-04 | -4.661 | TRANSAMINASE |
| CIN1 | 1.50E-04 | -4.642 | UNKNOWN |
| NUC1 | 1.50E-04 | 4.637 | ENDONUCLEASE |
| CCH1 | 1.50E-04 | -4.636 | (PUTATIVE) CA(2+) CHANNEL PROTEIN |
| DSS4 | 1.53E-04 | 4.63 | GDP/GTP EXCHANGE FACTOR FOR SEC4P |
| APA2 | 1.55E-04 | 4.625 | ATP ADENYLYLTRANSFERASE II |
| RTF1 | 1.55E-04 | 4.625 | REGULATOR OF SPT15 DNA BINDING PROPERTIE |
| MRT4 | 1.61E-04 | 4.613 | UNKNOWN |
| AME1 | 1.64E-04 | 4.605 | MICROTUBULE ASSOCIATED |
| MSG5 | 1.78E-04 | 4.583 | DUAL-SPECIFICITY PROTEIN PHOSPHATASE |
| POP4 | 1.85E-04 | 4.572 | RNASE P AND RNASE MRP SUBUNIT |
| RRP1 | 1.92E-04 | 4.562 | UNKNOWN |
| NAT2 | 1.93E-04 | 4.558 | N-ACETYLTRANSFERASE FOR N-TERMINAL METHI |
| ATC1 | 1.98E-04 | 4.548 | MEMBER OF BUD6P COMPLEX |
| SRB8 | 2.13E-04 | -4.527 | RNA POLYMERASE II MEDIATOR SUBUNIT |
| RAI1 | 2.14E-04 | 4.525 | UNKNOWN; SIMILAR TO C. ELEGANS DOM-3 (GB |
| PHD1 | 2.40E-04 | 4.491 | TRANSCRIPTION FACTOR |
| SPT2 | 2.45E-04 | 4.484 | HMG-LIKE NON-HISTONE PROTEIN |
| DAN1 | 2.46E-04 | 4.483 | UNKNOWN; INDUCED DURING ANAEROBIC GROWTH |
| ADE17 | 2.59E-04 | -4.465 | 5-AMINOIMIDAZOLE-4-CARBOXAMIDE RIBONUCLE |
| FET3 | 2.70E-04 | 4.454 | CELL SURFACE FERROXIDASE |
| SNC1 | 2.79E-04 | -4.444 | GOLGI V-SNARE |
| SPB1 | 3.16E-04 | 4.411 | RRNA METHYLTRANSFERASE (PUTATIVE) |
| DAL81 | 3.34E-04 | -4.392 | ACTIVATOR OF ALLANTOIN AND UREA CATABOLI |
| SSF1 | 3.37E-04 | 4.387 | UNKNOWN |
| TOA1 | 3.49E-04 | 4.374 | TFIIA 32 KD SUBUNIT |
| NMD3 | 3.54E-04 | 4.369 | NAM7P/UPF1P-INTERACTING PROTEIN |
| NOP8 | 3.66E-04 | 4.359 | UNKNOWN; NUCLEOLAR PROTEIN |
| PHO86 | 3.76E-04 | 4.351 | INORGANIC PHOSPHATE PERMEASE |
| NOP2 | 3.83E-04 | 4.346 | NUCLEOLAR PROTEIN |
| RNA15 | 3.87E-04 | 4.34 | CLEAVAGE/POLYADENYLATION FACTOR CF I COM |
| LYS20 | 4.03E-04 | 4.327 | HOMOCITRATE SYNTHASE |
| HIT1 | 4.49E-04 | 4.296 | REQUIRED FOR GROWTH AT HIGH TEMPERATURE |
| LYS9 | 4.55E-04 | 4.291 | SACCHAROPINE DEHYDROGENASE |
| TIR1 | 4.55E-04 | 4.29 | CELL WALL PROTEIN |
| MNE1 | 4.56E-04 | -4.288 | UNKNOWN; SIMILAR TO LUCILIA ILLUSTRIS MI |
| SSF2 | 4.61E-04 | 4.285 | SUPPRESSES G-PROTEIN BETA SUBUNIT MUTATI |
| GAR1 | 4.86E-04 | 4.267 | SNORNP PROTEIN |
| MIF2 | 4.86E-04 | 4.267 | CENTROMERE PROTEIN |
| DBP9 | 4.96E-04 | 4.26 | PUTATIVE RNA HELICASE |
| RPB3 | 5.15E-04 | 4.247 | RNA POLYMERASE II 45 KDA SUBUNI |
| EBP2 | 5.20E-04 | 4.243 | UNKNOWN; EBNA1-BINDING PROTEIN HOMOLOG |
| SAS10 | 5.20E-04 | 4.241 | NUCLEAR PROTEIN, REULATOR OF SILENCING A |
| SSU1 | 5.23E-04 | -4.236 | UNKNOWN; PLASMA MEMBRANE PROTEIN, MAJOR |
| CCC2 | 5.37E-04 | 4.227 | CU(2+)-TRANSPORTING ATPASE |
| SYF3 | 5.39E-04 | 4.225 | UNKNOWN; SYNTHETIC LETHAL WITH CDC40 |
| Cluster 22 |  |  |  |
| HRB1 | 1.36E-06 | 6.458 | RNA BINDING PROTEIN (PUTATIVE) |
| ALR1 | 1.20E-04 | 5.41 | ION TRANSPORTER, PUTATIVE |
| PMP2 | 2.23E-04 | 5.217 | REGULATES PLASMA MEMBRANE H+-ATPASE |
| Cluster 23 |  |  |  |
| MED11 | 9.37E-15 | 9.643 | RNA POLYMERASE II MEDIATOR SUBUNIT |
| SEC9 | 2.96E-14 | 9.381 | PLASMA MEMBRANE T-SNARE |
| THO1 | 2.96E-14 | 9.379 | UNKNOWN |
| LSM7 | 1.00E-11 | 8.334 | SIMILAR TO SNRNA-ASSOCIATED PROTEIN |
| TLG1 | 1.14E-11 | 8.295 | LATE GOLGI T-SNARE |
| NUP120 | 4.60E-10 | 7.611 | NUCLEAR PORE PROTEIN |
| SPR6 | 4.66E-10 | 7.596 | UNKNOWN |
| ADE16 | 8.93E-10 | 7.462 | 5-AMINOIMIDAZOLE-4-CARBOXAMIDE RIBONUCLE |
| SPT2 | 1.40E-09 | 7.365 | HMG-LIKE NON-HISTONE PROTEIN |
| MPT1 | 1.40E-09 | 7.36 | UNKNOWN |
| MSL1 | 1.40E-09 | -7.355 | U2 SNRNP PROTEIN |
| VPS38 | 2.45E-09 | 7.247 | UNKNOWN |
| HOC1 | 4.04E-09 | 7.142 | PUTATIVE MANNOSYLTRANSFERASE |
| BUR2 | 4.38E-09 | 7.12 | UNKNOWN |
| TCM62 | 2.12E-08 | -6.821 | CHAPERONE, MITOCHONDRIAL (PUTATIVE) |
| MRPL36 | 2.34E-08 | 6.79 | RIBOSOMAL PROTEIN, MITOCHONDRIAL L36 |
| HHO1 | 4.66E-08 | 6.65 | HISTONE H1 |
| RPS21A | 9.51E-07 | 6.035 | RIBOSOMAL PROTEIN S21A |
| RPN11 | 1.68E-06 | 5.903 | PUTATIVE GLOBAL REGULATOR |
| VPH2 | 1.72E-06 | 5.894 | VACUOLAR H+-ATPASE ASSEMBLY PROTEIN |
| FIG4 | 1.86E-06 | -5.864 | UNKNOWN; INDUCED BY MATING FACTOR |
| CHC1 | 2.36E-06 | -5.808 | CLATHRIN HEAVY CHAIN |
| SSO2 | 2.72E-06 | 5.771 | POST-GOLGI T-SNARE |
| LHS1 | 3.21E-06 | 5.734 | CHAPERONE; ER PROTEIN TRANSLOCATION |
| ERV14 | 3.51E-06 | 5.711 | UNKNOWN; ER-DERIVED VESICLE PROTEIN |
| RET3 | 4.12E-06 | 5.67 | VESICLE COAT COMPONENT |
| TIF35 | 4.12E-06 | 5.668 | TRANSLATION INITIATION FACTOR EIF3 SUBUN |
| RRP43 | 4.12E-06 | 5.667 | EXORIBONUCLEASE |
| TIF3 | 5.25E-06 | 5.614 | TRANSLATION INITIATION FACTOR EIF4B |
| RPS21B | 6.76E-06 | 5.559 | RIBOSOMAL PROTEIN S21B |
| SRP14 | 9.48E-06 | 5.484 | SIGNAL RECOGNITION PARTICLE SUBUNIT |
| SPC3 | 1.29E-05 | 5.411 | SIGNAL PEPTIDASE SUBUNIT |
| TFA1 | 1.77E-05 | 5.325 | TFIIE 66 KD SUBUNIT |
| VAM3 | 1.77E-05 | 5.32 | T-SNARE |
| RPP2A | 1.77E-05 | 5.318 | RIBOSOMAL PROTEIN P2A/L44, ACIDIC |
| SYT1 | 1.87E-05 | -5.299 | UNKNOWN; SIMILAR TO S. TYPHIMURIUM T1PA |
| ESS1 | 2.45E-05 | 5.238 | PEPTIDYL-PROLYL CIS/TRANS ISOMERASE |
| RPS5 | 2.60E-05 | 5.214 | RIBOSOMAL PROTEIN S5 |
| SGT1 | 2.60E-05 | 5.21 | UNKNOWN; SUPPRESSES SKP1 MUTATION |
| NPI46 | 3.54E-05 | 5.135 | PEPTIDYL-PROLYL CIS-TRANS ISOMERASE |
| GCD2 | 3.71E-05 | 5.12 | TRANSLATION INITIATION FACTOR EIF2B SUBU |
| AGP3 | 3.73E-05 | 5.115 | GENERAL AMINO ACID PERMEASE |
| RGA1 | 4.18E-05 | 5.083 | PUTATIVE GTPASE-ACTIVATING PROTEIN (GAP) |
| RPP2B | 4.34E-05 | 5.066 | RIBOSOMAL PROTEIN L45, ACIDIC |
| CMP2 | 4.34E-05 | -5.066 | CALCINEURIN CATALYTIC A SUBUNIT |
| MGM101 | 5.27E-05 | 5.02 | (PUTATIVE) NUCLEIC ACID INTERACTOR |
| GFA1 | 5.65E-05 | 4.997 | CHITIN BIOSYNTHESIS |
| NPR2 | 5.65E-05 | -4.994 | TRANSCRIPTION FACTOR |
| SPT6 | 7.80E-05 | 4.912 | ELONGATION FACTOR |
| CHS5 | 7.82E-05 | 4.902 | UNKNOWN |
| CTF4 | 9.23E-05 | -4.863 | POLYMERASE ALPHA BINDING PROTEIN |
| ZUO1 | 1.10E-04 | 4.818 | BINDS Z-DNA |
| YAF1 | 1.24E-04 | -4.789 | TRANSCRIPTIONAL ACTIVATOR OF POX1 |
| CNM67 | 1.39E-04 | 4.756 | SPINDLE POLE BODY COMPONENT |
| PEX19 | 1.39E-04 | -4.754 | UNKNOWN |
| THI3 | 1.49E-04 | -4.737 | ALPHA-KETOISOCAPROATE CARBOXYLASE |
| INP52 | 1.79E-04 | 4.684 | INOSITOL POLYPHOSPHATE 5-PHOSPHATASE |
| SNU71 | 1.81E-04 | 4.678 | U1 SNRNP PROTEIN |
| REG1 | 1.94E-04 | -4.66 | PROTEIN PHOSPHATASE REGULATOR |
| VMA22 | 2.54E-04 | 4.592 | VACUOLAR H+-ATPASE ASSEMBLY PROTEIN |
| SPC2 | 2.77E-04 | 4.567 | SIGNAL PEPTIDASE SUBUNIT |
| TPM1 | 3.01E-04 | 4.544 | TROPOMYOSIN |
| SOK2 | 3.01E-04 | 4.543 | UNKNOWN; SIMILAR TO SEVERAL TRANSCRIPTIO |
| RPL12B | 3.02E-04 | 4.541 | RIBOSOMAL PROTEIN L12B |
| RLP7 | 3.05E-04 | 4.536 | RIBOSOMAL PROTEIN L7 (PUTATIVE) |
| HCR1 | 3.31E-04 | 4.514 | UNKNOWN |
| SSS1 | 3.68E-04 | 4.486 | ER PROTEIN TRANSLOCATION COMPLEX SUBUNIT |
| TRM1 | 3.78E-04 | 4.473 | TRNA METHYLTRANSFERASE |
| RTG2 | 3.99E-04 | -4.455 | UNKNOWN |
| DBP9 | 4.02E-04 | 4.452 | PUTATIVE RNA HELICASE |
| APC9 | 4.25E-04 | -4.435 | ANAPHASE-PROMOTING COMPLEX SUBUNIT |
| SEC6 | 4.63E-04 | 4.413 | EXOCYST COMPLEX SUBUNIT |
| DAM1 | 4.70E-04 | 4.408 | UNKNOWN; INTERACTS WITH DUO1P AND MPS1P |
| HNT1 | 5.07E-04 | 4.388 | UNKNOWN; SIMILAR TO PROTEIN KINASE C INH |
| SEC13 | 5.35E-04 | 4.371 | VESICLE COAT COMPONENT |
| SUI3 | 5.35E-04 | 4.369 | TRANSLATION INITIATION FACTOR EIF2 BETA |
| ERO1 | 6.43E-04 | 4.321 | PROTEIN DISULFIDE BOND FORMATION IN THE |
| DBP5 | 6.54E-04 | 4.313 | RNA HELICASE |
| MCM6 | 6.65E-04 | 4.304 | MCM INITIATOR COMPLEX |
| SEC61 | 6.65E-04 | 4.304 | ER PROTEIN TRANSLOCATION COMPLEX SUBUNIT |
| RAD57 | 6.96E-04 | -4.29 | RECA HOMOLOG |
| MDM12 | 6.96E-04 | -4.289 | TRANSMEMBRANE PROTEIN |
| LSM2 | 7.31E-04 | 4.276 | CORE SNRNP PROTEIN |
| SSN3 | 8.08E-04 | -4.247 | RNA POLYMERASE II MEDIATOR SUBUNIT |
| REC102 | 8.21E-04 | 4.242 | DS BREAK FORMATION COMPLEX SUBUNIT |
| RPL38 | 8.34E-04 | 4.235 | RIBOSOMAL PROTEIN L38 |
| POP1 | 8.60E-04 | -4.226 | RNASE P AND RNASE MRP SUBUNIT |
| CKA1 | 8.70E-04 | 4.222 | CASEIN KINASE II, CATALYTIC SUBUNIT |
| MAC1 | 9.47E-04 | -4.195 | TRANSCRIPTION FACTOR |
| Cluster 24 |  |  |  |
| BUD3 | 2.37E-32 | 15.979 | BUD SITE SELECTION |
| SLA1 | 2.49E-23 | 12.524 | CORTICAL ACTIN ASSEMBLY |
| SEC23 | 3.45E-22 | 12.1 | VESICLE COAT COMPONENT |
| DHH1 | 1.06E-21 | 11.909 | RNA HELICASE |
| SEC31 | 3.86E-21 | 11.693 | VESICLE COAT COMPONENT |
| MDJ2 | 7.42E-21 | 11.563 | MITOCHONDRIAL CHAPERONIN |
| BEM2 | 4.30E-20 | 11.28 | GTPASE-ACTIVATING PROTEIN FOR RHO1P |
| YBT1 | 9.82E-19 | 10.786 | BILE ACID TRANSPORTER OF ABC FAMILY |
| NUP133 | 2.81E-18 | 10.612 | NUCLEAR PORE PROTEIN |
| SEC21 | 6.50E-18 | 10.472 | VESICLE COAT COMPONENT |
| VTH2 | 7.33E-18 | 10.433 | UNKNOWN; SIMILAR TO VPS10P |
| SSM4 | 1.06E-17 | 10.358 | UNKNOWN |
| SPT5 | 1.32E-17 | 10.316 | ELONGATION FACTOR |
| PAN1 | 1.53E-17 | 10.285 | ACTIN FILAMENT ORGANIZATION |
| RPA135 | 1.25E-16 | 9.937 | RNA POLYMERASE I 135 KD SUBUNIT |
| MGA2 | 3.60E-16 | 9.763 | CHROMATIN REMODELING (PUTATIVE) |
| FKS1 | 4.41E-16 | 9.719 | 1,3-BETA-D-GLUCAN SYNTHASE SUBUNIT |
| STE6 | 6.47E-16 | 9.647 | A-FACTOR EXPORTER (ABC SUPERFAMILY) |
| SLN1 | 9.18E-16 | 9.581 | TWO-COMPONENT PATHWAY |
| HOT1 | 9.18E-16 | 9.58 | UNKNOWN; NUCLEAR PROTEIN; SIMILAR TO MSN |
| RGR1 | 1.48E-15 | 9.5 | RNA POLYMERASE II MEDIATOR SUBUNIT |
| KAP95 | 2.18E-15 | 9.429 | BETA-KARYOPHERIN |
| POM152 | 2.36E-15 | 9.411 | NUCLEAR PORE PROTEIN |
| SEC24 | 3.64E-15 | 9.338 | VESICLE COAT COMPONENT |
| PDR5 | 3.93E-15 | 9.321 | TRANSPORTER |
| PDR3 | 5.74E-15 | 9.248 | TRANSCRIPTION FACTOR |
| NUP159 | 6.05E-15 | 9.232 | NUCLEAR PORE PROTEIN |
| RPG1 | 6.21E-15 | 9.223 | TRANSLATION INITIATION FACTOR EIF3 |
| ADE6 | 8.53E-15 | 9.169 | 5'-PHOSPHORIBOSYLFORMYL GLYCINAMIDINE SY |
| NAM7 | 4.99E-14 | 8.862 | RNA HELICASE, PUTATIVE |
| CRM1 | 5.87E-14 | 8.833 | NUCLEAR EXPORT FACTOR |
| CSE1 | 8.62E-14 | 8.767 | KINETOCHORE PROTEIN (PUTATIVE) |
| GCN1 | 9.93E-14 | 8.741 | TRANSLATION ACTIVATOR OF GCN4 |
| BCK1 | 1.44E-13 | 8.677 | SERINE-THREONINE PROTEIN KINASE |
| MSB2 | 2.53E-13 | 8.578 | UNKNOWN |
| RET1 | 6.88E-13 | 8.402 | RNA POLYMERASE III 130 KD SUBUNIT |
| LOS1 | 1.80E-12 | 8.228 | NUCLEAR PORE PROTEIN |
| KAP104 | 2.35E-12 | 8.175 | BETA-KARYOPHERIN |
| SKY1 | 2.90E-12 | 8.138 | PROTEIN KINASE |
| RSP5 | 4.75E-12 | 8.052 | UBIQUITIN-PROTEIN LIGASE (E3 ENZYME) |
| NUT1 | 5.78E-12 | 8.017 | NEGATIVE REGULATOR OF HO ENDONUCLEASE |
| WHI3 | 7.48E-12 | 7.971 | UNKNOWN |
| BUL1 | 1.06E-11 | 7.905 | UNKNOWN; BINDS RSP5P UBIQUITIN LIGASE |
| VIP1 | 1.50E-11 | 7.844 | UNKNOWN |
| UBP12 | 1.89E-11 | 7.797 | UBIQUITIN-SPECIFIC PROTEASE |
| SMC4 | 4.14E-11 | 7.651 | UNKNOWN |
| HCM1 | 4.27E-11 | 7.645 | FORKHEAD FAMILY OF DNA-BINDING PROTEINS |
| BUD7 | 5.21E-11 | 7.608 | UNKNOWN |
| TFP1 | 5.66E-11 | 7.589 | VACUOLAR H+-ATPASE SUBUNIT |
| MSB1 | 1.00E-10 | 7.486 | UNKNOWN |
| PYC2 | 2.17E-10 | 7.348 | PYRUVATE CARBOXYLASE 2 |
| SAE2 | 2.51E-10 | 7.319 | UNKNOWN |
| NUP157 | 2.79E-10 | 7.3 | NUCLEAR PORE PROTEIN |
| IRE1 | 3.39E-10 | 7.262 | SENSOR OF UNFOLDED PROTEINS IN THE ER |
| TUP1 | 3.88E-10 | 7.237 | GENERAL REPRESSOR |
| UBA1 | 4.39E-10 | 7.212 | E1-LIKE (UB.-ACTIVATING) ENZYME |
| NRK1 | 5.44E-10 | 7.172 | PROTEIN KINASE; INTERACTS WITH CDC31P |
| PFK1 | 7.73E-10 | 7.104 | PHOSPHOFRUCTOKINASE |
| RPO21 | 1.50E-09 | 6.975 | RNA POLYMERASE II 215 KD SUBUNIT |
| SFB3 | 1.63E-09 | 6.958 | UNKNOWN; BINDS SED3P AND SEC23P |
| VAC7 | 1.84E-09 | 6.934 | UNKNOWN; VACUOLAR INTEGRAL MEMBRANE PROT |
| APL5 | 2.17E-09 | 6.901 | AP-3 COMPLEX SUBUNIT |
| AKR1 | 2.17E-09 | 6.899 | ANKYRIN-CONTAINING PROTEIN |
| CDC60 | 3.26E-09 | 6.821 | TRNA SYNTHETASE, LEUCYL |
| NUP100 | 3.60E-09 | 6.802 | NUCLEAR PORE PROTEIN |
| NUP116 | 3.68E-09 | 6.797 | NUCLEAR PORE PROTEIN |
| SEC15 | 3.95E-09 | 6.782 | EXOCYST COMPLEX SUBUNIT |
| HRD3 | 4.40E-09 | 6.761 | HMG-COA REDUCTASE DEGRADATION |
| DOA1 | 4.57E-09 | 6.752 | UNKNOWN |
| PSD2 | 4.62E-09 | 6.749 | PHOSPHATIDYLSERINE DECARBOXYLASE 2 |
| DIS3 | 5.26E-09 | 6.722 | 3'-5' EXORIBONUCLEASE COMPLEX SUBUNIT |
| VTH1 | 8.47E-09 | 6.632 | UNKNOWN; SIMILAR TO VPS10P |
| PMT1 | 1.06E-08 | 6.588 | DOLICHYL PHOSPHATE-D-MANNOSE:PROTEIN O-D |
| STT3 | 1.09E-08 | 6.581 | OLIGOSACCHARYLTRANSFERASE COMPLEX ASSEMB |
| SSD1 | 1.12E-08 | 6.572 | PUTATIVE PROTEIN PHOSPHATASE |
| SPT23 | 1.38E-08 | 6.531 | TRANSCRIPTION FACTOR |
| TAF90 | 1.45E-08 | 6.521 | TFIID 90 KD SUBUNIT |
| LOC7 | 1.54E-08 | 6.507 | UNKNOWN |
| SRO9 | 1.90E-08 | 6.466 | ACTIN FILAMENT ORGANIZATION |
| ARG81 | 2.11E-08 | 6.443 | TRANSCRIPTION FACTOR |
| USO1 | 2.39E-08 | 6.415 | SNARE DOCKING COMPLEX ASSEMBLY |
| PMA1 | 2.52E-08 | 6.405 | PLASMA MEMBRANE H+-ATPASE |
| PEX1 | 2.54E-08 | 6.402 | ATPASE (PUTATIVE) |
| LAS21 | 2.64E-08 | 6.392 | MAJOR FACILITATOR SUPERFAMILY |
| ECM30 | 2.73E-08 | 6.384 | UNKNOWN |
| YRF1-5 | 2.85E-08 | 6.375 | Y' HELICASE (SUBTELOMERICALLY-ENCODED) |
| CHD1 | 2.98E-08 | 6.364 | CHROMODOMAIN-HELICASE-DNA-BINDING (CHD) |
| VAS1 | 3.46E-08 | 6.333 | TRNA SYNTHETASE, VALYL |
| MES1 | 3.64E-08 | 6.322 | TRNA SYNTHETASE, METHIONYL |
| RIS1 | 4.87E-08 | 6.265 | SNF2 FAMILY DNA-DEPENDENT ATPASE |
| STE23 | 7.06E-08 | 6.19 | A-FACTOR PROCESSING PROTEASE (PUTATIVE) |
| TRS120 | 7.58E-08 | 6.174 | TRANSPORT PROTEIN PARTICLE (TRAPP) SUBUN |
| DED1 | 8.03E-08 | 6.161 | ATP-DEPENDENT RNA HELICASE |
| KEL1 | 8.40E-08 | 6.152 | NULL MUTANT HAS MATING DEFECT |
| KAP122 | 8.42E-08 | 6.15 | KARYOPHERIN-BETA FAMILY PROTEIN |
| INP53 | 8.84E-08 | 6.139 | INOSITOL POLYPHOSPHATE 5-PHOSPHATASE |
| RRP5 | 9.56E-08 | 6.121 | UNKNOWN; REQUIRED FOR PRE-RRNA CLEAVAGE |
| SCP160 | 1.06E-07 | 6.101 | UNKNOWN |
| PFK2 | 1.13E-07 | 6.084 | PHOSPHOFRUCTOKINASE |
| Cluster 25 |  |  |  |
| TAF19 | 5.68E-08 | 7.209 | TFIID 19 KD SUBUNIT |
| HKR1 | 9.42E-08 | 6.995 | UNKNOWN; CELL SURFACE PROTEIN |
| MAD1 | 1.24E-07 | 6.847 | SPINDLE CHECKPOINT COMPLEX SUBUNIT |
| CTF19 | 1.24E-07 | 6.819 | KINETOCHORE PROTEIN |
| RCK1 | 4.57E-07 | 6.474 | PROTEIN KINASE |
| EFD1 | 6.71E-07 | 6.353 | UNKNOWN |
| MBP1 | 7.52E-07 | 6.312 | TRANSCRIPTION FACTOR |
| PGS1 | 2.41E-06 | -6.049 | PHOSPHATIDYLGLYCEROPHOSPHATE SYNTHASE |
| MSS11 | 2.41E-06 | 6.024 | (PUTATIVE) TRANSCRIPTIONAL REPRESSOR |
| MCX1 | 3.65E-06 | 5.925 | CHAPERONE, MITOCHONDRIAL (PUTATIVE) |
| MLP1 | 3.94E-06 | 5.886 | MYOSIN-LIKE PROTEIN |
| BEM3 | 4.91E-06 | 5.823 | GTPASE-ACTIVATING PROTEIN FOR CDC42P |
| PRP6 | 6.56E-06 | 5.751 | U4/U6 SNRNP PROTEIN |
| PHO81 | 1.09E-05 | 5.638 | PHO85P KINASE INHIBITOR |
| SKT5 | 1.64E-05 | 5.53 | CHITIN SYNTHASE REGULATOR |
| VIP1 | 1.64E-05 | 5.526 | UNKNOWN |
| RFA1 | 1.72E-05 | 5.485 | REPLICATION FACTOR A, 69 KD SUBUNIT |
| HRR25 | 1.74E-05 | 5.469 | CASEIN KINASE I ISOFORM |
| RLF2 | 2.00E-05 | 5.425 | CHROMATIN ASSEMBLY FACTOR I SUBUNIT |
| RPH1 | 2.01E-05 | 5.418 | TRANSCRIPTIONAL REPRESSOR OF PHR1 |
| DSK2 | 2.14E-05 | 5.398 | UBIQUITIN-LIKE PROTEIN |
| ESR1 | 2.94E-05 | 5.325 | PI KINASE HOMOLOG |
| SPT7 | 2.94E-05 | 5.32 | HISTONE ACETYLTRANSFERASE COMPLEX SUBUNI |
| HIR3 | 3.21E-05 | 5.295 | REGULATOR OF HISTONE TRANSCRIPTION |
| SET2 | 3.36E-05 | 5.273 | TRANSCRIPTIONAL REPRESSOR OF GAL4 |
| TIM54 | 3.36E-05 | 5.268 | INNER MEMBRANE TRANSLOCASE COMPONENT |
| ASK10 | 3.36E-05 | 5.257 | ENHANCER OF SKN7-DEPENDENT TRANSCRIPTION |
| NUP145 | 3.36E-05 | 5.248 | NUCLEAR PORE PROTEIN |
| IME2 | 3.56E-05 | -5.223 | SERINE/THREONINE PROTEIN KINASE |
| KIN28 | 3.56E-05 | 5.217 | PROTEIN KINASE; ALSO TFIIH SUBUNIT |
| MAL13 | 3.56E-05 | -5.217 | REGULATOR OF MALTOSE METABOLIC GENES |
| RPA190 | 3.64E-05 | 5.208 | RNA POLYMERASE I 190 KD SUBUNIT |
| PHO84 | 4.54E-05 | 5.151 | INORGANIC PHOSPHATE PERMEASE |
| POP1 | 4.79E-05 | 5.136 | RNASE P AND RNASE MRP SUBUNIT |
| RLM1 | 4.81E-05 | 5.127 | MADS BOX TRANSCRIPTION FACTOR |
| IFH1 | 5.17E-05 | 5.107 | UNKNOWN |
| TOP1 | 5.77E-05 | 5.072 | TOPOISOMERASE I |
| CDC54 | 7.76E-05 | 4.991 | MCM INITIATOR COMPLEX |
| TRA1 | 7.76E-05 | 4.991 | UNKNOWN; SIMILAR TO HUMAN TR-AP |
| MAK16 | 7.76E-05 | 4.988 | UNKNOWN; ESSENTIAL GENE |
| GPD2 | 8.15E-05 | 4.973 | GLYCEROL-3-PHOSPHATE DEHYDROGENASE |
| CDC24 | 9.50E-05 | 4.933 | GDP/GTP EXCHANGE FACTOR FOR CDC42P |
| DEG1 | 1.00E-04 | 4.914 | PSEUDOURIDINE SYNTHASE |
| NNF1 | 1.05E-04 | 4.902 | NUCLEAR ENVELOPE PROTEIN |
| MAL12 | 1.13E-04 | -4.882 | ALPHA-GLUCOSIDASE |
| SPL2 | 1.15E-04 | 4.874 | PROTEIN KINASE INHIBITOR |
| FUN30 | 1.25E-04 | 4.842 | UNKNOWN; SIMILAR TO SNF2 TRANSCRIPTIONAL |
| NHX1 | 1.25E-04 | -4.841 | NA+/H+ ANTIPORTER |
| IPL1 | 1.25E-04 | 4.839 | PROTEIN KINASE |
| SUV3 | 1.25E-04 | 4.834 | RNA HELICASE |
| BOI1 | 1.53E-04 | 4.784 | BINDS BEM1P |
| ZDS1 | 1.75E-04 | 4.743 | PERIPHERAL PLASMA MEMBRANE PROTEIN |
| RAD10 | 1.77E-04 | 4.738 | SSDNA ENDONUCLEASE |
| SPC72 | 1.78E-04 | 4.733 | SPINDLE POLE BODY COMPONENT |
| NCE4 | 1.92E-04 | 4.714 | NEGATIVE REGULATOR OF CTS1 EXPRESSION |
| PRP8 | 1.96E-04 | 4.706 | U4/U6, U5 SNRNP PROTEIN |
| SMC3 | 2.20E-04 | 4.677 | COHESIN |
| BAS1 | 2.26E-04 | 4.667 | TRANSCRIPTION FACTOR |
| VPS27 | 2.34E-04 | 4.656 | COMPONENT OF CLASS E PROTEIN COMPLEX |
| CDH1 | 2.34E-04 | -4.651 | CYCLIN DEGRADATION |
| SIR3 | 2.49E-04 | 4.633 | NUCLEAR PROTEIN, REULATOR OF SILENCING A |
| RAD14 | 2.49E-04 | 4.633 | REQUIRED FOR INCISION STEP |
| TEA1 | 2.69E-04 | 4.603 | TY1 ENHANCER ACTIVATOR |
| ULP1 | 2.91E-04 | 4.581 | UBL (UBIQUITIN-LIKE PROTEIN) - SPECIFIC |
| PDR10 | 3.10E-04 | 4.56 | ATP-BINDING CASSETTE (ABC) FAMILY |
| PRP31 | 3.10E-04 | 4.559 | U4/U6, U5 SNRNP PROTEIN |
| FUN14 | 3.16E-04 | 4.551 | UNKNOWN |
| ERP6 | 3.56E-04 | -4.517 | UNKNOWN |
| ISM1 | 3.73E-04 | 4.498 | TRNA SYNTHETASE, MITOCHONDRIAL, ISOLEUCY |
| NUP2 | 3.98E-04 | 4.479 | NUCLEAR PORE PROTEIN |
| SUM1 | 4.73E-04 | 4.431 | NUCLEAR PROTEIN |
| NUM1 | 4.76E-04 | 4.425 | UNKNOWN |
| GPI12 | 4.77E-04 | -4.423 | N-ACETYLGLUCOSAMINYLPHOSPHATIDYLINOSITOL |
| MET12 | 4.78E-04 | 4.417 | METHYLENETETRAHYDROFOLATE REDUCTASE |
| NAB3 | 4.96E-04 | 4.401 | NUCLEAR POLYADENYLATED RNA-BINDING PROTE |
| CNE1 | 5.08E-04 | 4.394 | CALNEXIN AND CALRETICULIN HOMOLOG |
| FUS3 | 5.45E-04 | 4.374 | PROTEIN KINASE |
| SDS23 | 6.03E-04 | 4.346 | UNKNOWN; SIMILAR TO S. POMBE SDS23 |
| IDI1 | 6.12E-04 | 4.338 | ISOPENTENYL-DIPHOSPHATE DELTA-ISOMERASE |
| NAB2 | 6.15E-04 | 4.335 | POLY(A)+RNA BINDING PROTEIN |
| UBP13 | 6.17E-04 | 4.332 | UBIQUITIN CARBOXYL-TERMINAL HYDROLASE |
| SEC5 | 6.54E-04 | 4.311 | EXOCYST COMPLEX SUBUNIT |
| PYC1 | 6.54E-04 | 4.31 | PYRUVATE CARBOXYLASE 1 |
| HEM14 | 6.64E-04 | 4.305 | PROTOPORPHYRINOGEN OXIDASE |
| SPP41 | 6.80E-04 | 4.297 | NEGATIVE REGULATOR OF SPLICEOSOME GENES |
| SAP185 | 6.80E-04 | 4.295 | SIT4P-ASSOCIATED PROTEIN |
| APG14 | 6.84E-04 | -4.29 | UNKNOWN; INTERACTS WITH APG6P/VPS30P |
| DST1 | 7.13E-04 | 4.277 | ELONGATION FACTOR TFIIS |
| SRP72 | 7.82E-04 | 4.247 | SIGNAL RECOGNITION PARTICLE SUBUNIT |
| MYO2 | 8.44E-04 | 4.226 | MYOSIN, CLASS V |
| DBF4 | 8.44E-04 | 4.224 | CDC7P (KINASE) REGULATOR |
| TRM3 | 8.59E-04 | 4.216 | TRNA ROBOSE METHYLASE |
| KRE5 | 8.87E-04 | 4.201 | UNKNOWN; ER RESIDENT GLYCOPROTEIN |
| CAR2 | 8.88E-04 | 4.199 | ORNITHINE AMINOTRANSFERASE |
| URK1 | 9.52E-04 | 4.18 | URIDINE KINASE |
| Cluster 26 |  |  |  |
| --- |  |  |  |
