# Supplementary material for: Dual activation of pathways regulated by steroid receptors and peptide growth factors in primary prostate cancer revealed by Factor Analysis of microarray data
Source: BMC Genomics. 2005 Aug 17;6:109. doi: 10.1186/1471-2164-6-109 (PMC1239914; doi:10.1186/1471-2164-6-109)
Supplement: Additional File 2 — List of genes significantly associated to each cluster in the prostate cancer dataset (q-value < 10-3). [file 1471-2164-6-109-S2.doc]

**Table 2.** Genes most relevant according to FADA for the five groups of the Welsh dataset [2], selected as described in Methods. For each gene, the *t-*test Q-value, the t-statistic, the gene name, and gene description is shown. Samples forming each group can be found in Figure 2.

| **Gene** | **Q-value** | **T-statistic** | **Description** |
| --- | --- | --- | --- |
| Cluster 1 |  |  |  |
| CLPTM1 | 1.02E-19 | -17.136 | cleft lip and palate associated transmembrane protein 1 |
| SCRN1 | 6.58E-19 | -16.181 | secernin 1 |
| KCNJ3 | 1.49E-14 | 12.641 | potassium inwardly-rectifying channel, subfamily J, member 3 |
| JAK1 | 1.07E-13 | -11.937 | Janus kinase 1 (a protein tyrosine kinase) |
| PSMD5 | 4.46E-13 | -11.435 | proteasome (prosome, macropain) 26S subunit, non-ATPase, 5 |
| SLC1A5 | 6.19E-13 | -11.283 | solute carrier family 1 (neutral amino acid transporter), member 5 |
| VEGFB | 1.16E-12 | -11.049 | vascular endothelial growth factor B |
| PLSCR1 | 2.44E-12 | -10.789 | phospholipid scramblase 1 |
| ST7 | 5.98E-12 | 10.462 | suppression of tumorigenicity 7 |
| GTF2E1 | 1.54E-11 | 10.161 | general transcription factor IIE, polypeptide 1, alpha 56kDa |
| PBX3 | 2.59E-11 | -9.988 | Pre-B-cell leukemia transcription factor 3 |
| STAT6 | 4.11E-11 | -9.834 | signal transducer and activator of transcription 6, interleuk |
| CAST | 4.75E-11 | -9.771 | calpastatin |
| HLA-E | 5.59E-11 | -9.705 | major histocompatibility complex, class I, E |
| OAT | 8.67E-11 | -9.563 | ornithine aminotransferase (gyrate atrophy) |
| SP100 | 1.05E-10 | -9.485 | nuclear antigen Sp100 |
| GAGE1 | 1.05E-10 | 9.463 | /// GAGE2 /// GAGE4 /// GAGE5 /// GAGE6 /// GAGE7 /// GAGE7B /// GAGE8 |
| SPTBN1 | 1.05E-10 | -9.462 | spectrin, beta, non-erythrocytic 1 |
| NME4 | 2.02E-10 | -9.248 | non-metastatic cells 4, protein expressed in |
| ABCD3 | 2.36E-10 | 9.193 | ATP-binding cassette, sub-family D (ALD), member 3 |
| DDAH2 | 5.01E-10 | -8.959 | dimethylarginine dimethylaminohydrolase 2 |
| HEG | 7.89E-10 | -8.822 | HEG homolog |
| B2M | 9.94E-10 | -8.747 | beta-2-microglobulin |
| U2AF2 | 1.36E-09 | 8.640 | U2 (RNU2) small nuclear RNA auxiliary factor 2 |
| C10orf6 | 3.38E-09 | -8.379 | chromosome 10 open reading frame 6 |
| CALM2 | 3.38E-09 | -8.372 | calmodulin 2 (phosphorylase kinase, delta) |
| CASP4 | 5.35E-09 | -8.237 | caspase 4, apoptosis-related cysteine protease |
| PGM1 | 6.93E-09 | -8.158 | phosphoglucomutase 1 |
| UGT2B15 | 7.85E-09 | 8.116 | UDP glycosyltransferase 2 family, polypeptide B15 |
| CNN3 | 8.98E-09 | -8.071 | calponin 3, acidic |
| HLA-A | 1.02E-08 | -8.028 | major histocompatibility complex, class I, A |
| DKFZp667G2110 | 1.18E-08 | -7.982 | hypothetical protein DKFZp667G2110 |
| GNE | 1.19E-08 | -7.970 | glucosamine (UDP-N-acetyl)-2-epimerase/N-acetylmannosamine ki |
| DST | 1.37E-08 | -7.927 | dystonin |
| ABCC6 | 2.28E-08 | 7.780 | ATP-binding cassette, sub-family C (CFTR/MRP), member 6 |
| NMI | 2.88E-08 | -7.710 | N-myc (and STAT) interactor |
| MAP3K4 | 3.83E-08 | -7.626 | mitogen-activated protein kinase kinase kinase 4 |
| GRB10 | 4.05E-08 | 7.603 | growth factor receptor-bound protein 10 |
| TSNAX | 4.05E-08 | 7.599 | translin-associated factor X |
| TLE4 | 4.36E-08 | -7.568 | transducin-like enhancer of split 4 (E(sp1) homolog, Drosophi |
| CD44 | 4.36E-08 | -7.566 | CD44 antigen (homing function and Indian blood group system) |
| MAGEA5 | 4.97E-08 | 7.525 | melanoma antigen family A, 5 |
| DKFZP564J0123 | 5.75E-08 | 7.476 | nuclear protein E3-3 |
| RPS6KA3 | 5.75E-08 | -7.474 | ribosomal protein S6 kinase, 90kDa, polypeptide 3 |
| TMAP1 | 7.57E-08 | -7.387 | transmembrane anchor protein 1 |
| PPP1R15A | 1.18E-07 | -7.259 | protein phosphatase 1, regulatory (inhibitor) subunit 15A |
| CRAT | 1.43E-07 | -7.200 | carnitine acetyltransferase |
| RAP1B | 1.49E-07 | -7.184 | RAP1B, member of RAS oncogene family |
| ANXA2 | 1.58E-07 | -7.165 | annexin A2 |
| KCTD12 | 1.65E-07 | -7.147 | potassium channel tetramerisation domain containing 12 |
| C20orf18 | 1.77E-07 | 7.119 | chromosome 20 open reading frame 18 |
| PTP4A2 | 1.77E-07 | -7.118 | protein tyrosine phosphatase type IVA, member 2 |
| RAP140 | 2.38E-07 | -7.031 | retinoblastoma-associated protein 140 |
| UQCRC2 | 2.42E-07 | 7.018 | ubiquinol-cytochrome c reductase core protein II |
| KLHL20 | 2.42E-07 | 7.017 | kelch-like 20 (Drosophila) |
| STOM | 2.72E-07 | -6.980 | stomatin |
| TARS | 3.14E-07 | 6.938 | threonyl-tRNA synthetase |
| NNT | 3.42E-07 | -6.907 | nicotinamide nucleotide transhydrogenase |
| HTATIP2 | 3.62E-07 | -6.887 | HIV-1 Tat interactive protein 2, 30kDa |
| NAPG | 3.75E-07 | 6.872 | N-ethylmaleimide-sensitive factor attachment protein, gamma |
| GBP1 | 3.75E-07 | -6.870 | guanylate binding protein 1, interferon-inducible, 67kDa |
| PSMB4 | 3.76E-07 | 6.865 | proteasome (prosome, macropain) subunit, beta type, 4 |
| LOC149603 | 4.02E-07 | 6.843 | hypothetical protein LOC149603 |
| KIAA1128 | 4.62E-07 | -6.801 | KIAA1128 |
| HOXA11 | 5.21E-07 | -6.762 | homeo box A11 |
| NIPSNAP1 | 5.40E-07 | 6.745 | nipsnap homolog 1 (C. elegans) |
| PRKD1 | 5.40E-07 | 6.744 | protein kinase D1 |
| ANKRD15 | 5.88E-07 | -6.718 | ankyrin repeat domain 15 |
| PCCB | 7.26E-07 | 6.658 | propionyl Coenzyme A carboxylase, beta polypeptide |
| CYR61 | 7.83E-07 | -6.631 | cysteine-rich, angiogenic inducer, 61 |
| BAG2 | 7.83E-07 | -6.632 | BCL2-associated athanogene 2 |
| NMB | 1.11E-06 | -6.534 | neuromedin B |
| ASXL1 | 1.19E-06 | -6.510 | additional sex combs like 1 (Drosophila) |
| DAB2 | 1.32E-06 | -6.481 | disabled homolog 2, mitogen-responsive phosphoprotein (Drosophila) |
| ATP1B1 | 1.39E-06 | 6.463 | ATPase, Na+/K+ transporting, beta 1 polypeptide |
| ZNF238 | 1.42E-06 | -6.454 | zinc finger protein 238 |
| MAFF | 1.47E-06 | -6.441 | v-maf musculoaponeurotic fibrosarcoma oncogene homolog F (avi |
| AP3S2 | 2.05E-06 | 6.345 | adaptor-related protein complex 3, sigma 2 subunit |
| FARP1 | 2.40E-06 | -6.299 | FERM, RhoGEF (ARHGEF) and pleckstrin domain protein 1 (chondr |
| KCNH2 | 2.46E-06 | -6.289 | potassium voltage-gated channel, subfamily H (eag-related), member 2 |
| TNFAIP2 | 2.49E-06 | -6.282 | tumor necrosis factor, alpha-induced protein 2 |
| MCFD2 | 2.81E-06 | -6.247 | multiple coagulation factor deficiency 2 |
| FYN | 3.14E-06 | -6.214 | FYN oncogene related to SRC, FGR, YES |
| SMARCA5 | 4.14E-06 | -6.137 | SWI/SNF related, matrix associated, actin dependent regulator |
| DUSP6 | 4.68E-06 | -6.096 | dual specificity phosphatase 6 |
| LGALS3 | 4.94E-06 | -6.079 | /// GALIG lectin, galactoside-binding, soluble, 3 (gale |
| PCK2 | 4.94E-06 | 6.077 | phosphoenolpyruvate carboxykinase 2 (mitochondrial) |
| AARS | 5.06E-06 | 6.064 | alanyl-tRNA synthetase |
| GCLC | 5.36E-06 | 6.042 | glutamate-cysteine ligase, catalytic subunit |
| NDUFS2 | 5.36E-06 | 6.041 | NADH dehydrogenase (ubiquinone) Fe-S protein 2, 49kDa (NADH-c |
| C19orf13 | 5.69E-06 | -6.018 | chromosome 19 open reading frame 13 |
| LEPROT | 6.07E-06 | -5.999 | leptin receptor overlapping transcript |
| IFI16 | 6.31E-06 | -5.986 | interferon, gamma-inducible protein 16 |
| PHGDH | 7.11E-06 | 5.951 | phosphoglycerate dehydrogenase |
| PIK3CA | 7.17E-06 | -5.945 | phosphoinositide-3-kinase, catalytic, alpha polypeptide |
| IER3 | 7.42E-06 | -5.931 | immediate early response 3 |
| ZNF22 | 7.42E-06 | -5.928 | zinc finger protein 22 (KOX 15) |
| SMARCA1 | 7.57E-06 | -5.918 | SWI/SNF related, matrix associated, actin dependent regulator of chro |
| DAP3 | 7.75E-06 | 5.910 | death associated protein 3 |
| Cluster 2 |  |  |  |
| FSCN1 | 4.65E-19 | 16.330 | fascin homolog 1, actin-bundling protein (Strongylocentrotus purpurat |
| LOC157567 | 3.52E-18 | -15.350 | hypothetical protein LOC157567 |
| KIAA0240 | 1.43E-17 | -14.720 | KIAA0240 |
| RBL2 | 2.38E-17 | -14.447 | retinoblastoma-like 2 (p130) |
| DDX17 | 3.59E-17 | -14.232 | DEAD (Asp-Glu-Ala-Asp) box polypeptide 17 |
| MGC2650 | 1.71E-16 | -13.652 | hypothetical protein MGC2650 |
| ZNF91 | 3.03E-16 | -13.415 | zinc finger protein 91 (HPF7, HTF10) |
| POGZ | 7.09E-16 | -13.063 | pogo transposable element with ZNF domain |
| SMARCA2 | 7.09E-16 | -13.057 | SWI/SNF related, matrix associated, actin dependent regulator of chro |
| BTG2 | 7.86E-16 | -12.990 | BTG family, member 2 |
| AGL | 4.97E-15 | -12.371 | amylo-1, 6-glucosidase, 4-alpha-glucanotransferase (glycogen debranch |
| YT521 | 4.97E-15 | -12.350 | splicing factor YT521-B |
| RANBP2 | 2.35E-14 | -11.845 | RAN binding protein 2 |
| ZHX2 | 3.74E-14 | -11.682 | zinc fingers and homeoboxes 2 |
| GSTO1 | 4.03E-14 | 11.630 | glutathione S-transferase omega 1 |
| ZNF24 | 4.03E-14 | -11.617 | Zinc finger protein 24 (KOX 17) |
| FLJ21439 | 4.75E-14 | -11.550 | hypothetical protein FLJ21439 |
| C2orf23 | 7.29E-14 | -11.404 | chromosome 2 open reading frame 23 |
| GPC3 | 8.31E-14 | -11.337 | glypican 3 |
| BZRAP1 | 8.31E-14 | -11.333 | benzodiazapine receptor (peripheral) associated protein 1 |
| PER2 | 8.91E-14 | -11.297 | period homolog 2 (Drosophila) |
| MRCL3 | 1.10E-13 | 11.221 | myosin regulatory light chain MRCL3 |
| ITPKB | 1.12E-13 | -11.201 | inositol 1,4,5-trisphosphate 3-kinase B |
| CLK1 | 1.25E-13 | -11.156 | CDC-like kinase 1 |
| CIRBP | 1.34E-13 | -11.123 | cold inducible RNA binding protein |
| FLJ35348 | 1.44E-13 | -11.090 | FLJ35348 |
| ATRX | 1.58E-13 | -11.050 | alpha thalassemia/mental retardation syndrome X-linked (RAD54 homolog |
| CTSC | 1.81E-13 | 10.991 | cathepsin C |
| ARPC1B | 1.96E-13 | 10.957 | actin related protein 2/3 complex, subunit 1B, 41kDa |
| CECR7 | 2.02E-13 | -10.930 | Similar to acetyl-Coenzyme A synthetase 3; medium-chain acyl-CoA synt |
| DMXL1 | 2.02E-13 | -10.928 | Dmx-like 1 |
| LOC153561 | 2.12E-13 | -10.905 | /// LOC402223 /// LOC441066 hypothetical protein LOC153561 /// si |
| HOXB13 | 3.36E-13 | -10.761 | homeo box B13 |
| TAX1BP3 | 3.91E-13 | 10.691 | Tax1 (human T-cell leukemia virus type I) binding protein 3 |
| TMSB10 | 4.05E-13 | 10.671 | thymosin, beta 10 |
| IQGAP2 | 4.05E-13 | -10.666 | IQ motif containing GTPase activating protein 2 |
| ABAT | 4.23E-13 | -10.645 | 4-aminobutyrate aminotransferase |
| CCNG2 | 4.23E-13 | -10.638 | Cyclin G2 |
| SHC1 | 4.60E-13 | 10.606 | SHC (Src homology 2 domain containing) transforming protein 1 |
| PSD4 | 4.63E-13 | -10.598 | pleckstrin and Sec7 domain containing 4 |
| TRIM33 | 4.90E-13 | -10.575 | Tripartite motif-containing 33 |
| EPM2AIP1 | 5.38E-13 | -10.541 | EPM2A (laforin) interacting protein 1 |
| CBX7 | 5.55E-13 | -10.527 | chromobox homolog 7 |
| RSBN1 | 5.67E-13 | -10.513 | round spermatid basic protein 1 |
| TM4SF2 | 6.78E-13 | -10.454 | transmembrane 4 superfamily member 2 |
| PCF11 | 6.78E-13 | -10.449 | pre-mRNA cleavage complex II protein Pcf11 |
| ACPP | 6.86E-13 | -10.438 | acid phosphatase, prostate |
| FLJ30092 | 6.86E-13 | -10.431 | AF-1 specific protein phosphatase |
| PP784 | 6.86E-13 | -10.428 | PP784 protein |
| CAP350 | 7.29E-13 | -10.405 | centrosome-associated protein 350 |
| SETBP1 | 8.06E-13 | -10.370 | SET binding protein 1 |
| KIAA0711 | 1.04E-12 | -10.290 | KIAA0711 gene product |
| MYCBP2 | 1.08E-12 | -10.277 | MYC binding protein 2 |
| PSMD2 | 1.13E-12 | 10.255 | proteasome (prosome, macropain) 26S subunit, non-ATPase, 2 |
| SORL1 | 1.13E-12 | -10.252 | Sortilin-related receptor, L(DLR class) A repeats-containing |
| FLJ13910 | 1.25E-12 | -10.218 | hypothetical protein FLJ13910 |
| THUMPD1 | 1.45E-12 | -10.171 | THUMP domain containing 1 |
| PPIL2 | 1.45E-12 | -10.165 | peptidylprolyl isomerase (cyclophilin)-like 2 |
| TNRC15 | 1.48E-12 | -10.147 | trinucleotide repeat containing 15 |
| SMCX | 1.48E-12 | -10.146 | Smcy homolog, X-linked (mouse) |
| ITGB4BP | 1.56E-12 | 10.125 | integrin beta 4 binding protein |
| RPL3 | 1.56E-12 | -10.122 | ribosomal protein L3 |
| AUH | 1.64E-12 | -10.099 | AU RNA binding protein/enoyl-Coenzyme A hydratase |
| RUTBC1 | 1.64E-12 | -10.097 | RUN and TBC1 domain containing 1 |
| DRAP1 | 1.64E-12 | 10.095 | DR1-associated protein 1 (negative cofactor 2 alpha) |
| COBLL1 | 1.65E-12 | -10.089 | COBL-like 1 |
| UTY | 1.79E-12 | -10.058 | ubiquitously transcribed tetratricopeptide repeat gene, Y-lin |
| FUBP1 | 1.81E-12 | -10.051 | Far upstream element (FUSE) binding protein 1 |
| PLAU | 1.87E-12 | 10.038 | plasminogen activator, urokinase |
| RAB32 | 1.95E-12 | 10.023 | RAB32, member RAS oncogene family |
| ZNF42 | 2.12E-12 | -9.994 | zinc finger protein 42 (myeloid-specific retinoic acid-responsive) |
| PFN1 | 2.19E-12 | 9.981 | profilin 1 |
| SRRM2 | 2.19E-12 | -9.977 | serine/arginine repetitive matrix 2 |
| SMCY | 2.32E-12 | -9.958 | Smcy homolog, Y-linked (mouse) |
| NEBL | 2.58E-12 | -9.923 | nebulette |
| HLA-DRA | 2.70E-12 | -9.906 | major histocompatibility complex, class II, DR alpha |
| ZNF250 | 2.70E-12 | -9.904 | zinc finger protein 250 |
| LOC92249 | 2.70E-12 | -9.899 | hypothetical protein LOC92249 |
| CAPN2 | 2.70E-12 | 9.895 | calpain 2, (m/II) large subunit |
| ZNF262 | 2.87E-12 | -9.875 | zinc finger protein 262 |
| FAAH | 2.88E-12 | -9.871 | fatty acid amide hydrolase |
| ZNF297 | 3.56E-12 | -9.807 | zinc finger protein 297 |
| EIF4A2 | 3.69E-12 | -9.794 | eukaryotic translation initiation factor 4A, isoform 2 |
| LOC255458 | 4.15E-12 | -9.755 | hypothetical protein LOC255458 |
| MRLC2 | 4.73E-12 | 9.713 | myosin regulatory light chain MRLC2 |
| KIAA0146 | 4.73E-12 | -9.711 | KIAA0146 protein |
| PKM2 | 5.53E-12 | 9.664 | pyruvate kinase, muscle |
| PDCD4 | 5.53E-12 | -9.661 | programmed cell death 4 (neoplastic transformation inhibitor) |
| DKFZp547K1113 | 5.96E-12 | -9.637 | Hypothetical protein DKFZp547K1113 |
| CD74 | 5.98E-12 | -9.633 | CD74 antigen (invariant polypeptide of major histocompatibility compl |
| RPS4X | 6.34E-12 | -9.613 | ribosomal protein S4, X-linked |
| KIF5C | 6.74E-12 | -9.593 | kinesin family member 5C |
| ADCY1 | 6.80E-12 | -9.586 | adenylate cyclase 1 (brain) |
| USP7 | 6.80E-12 | -9.584 | Ubiquitin specific protease 7 (herpes virus-associated) |
| EBNA1BP2 | 6.80E-12 | 9.582 | EBNA1 binding protein 2 |
| PIK3R1 | 7.12E-12 | -9.564 | phosphoinositide-3-kinase, regulatory subunit 1 (p85 alpha) |
| CPEB3 | 7.12E-12 | -9.564 | cytoplasmic polyadenylation element binding protein 3 |
| AHSA2 | 7.45E-12 | -9.548 | AHA1, activator of heat shock 90kDa protein ATPase homolog 2 |
| EPHA3 | 7.67E-12 | -9.537 | EPH receptor A3 |
| Cluster 3 |  |  |  |
| NR2F2 | 8.67E-23 | -19.809 | nuclear receptor subfamily 2, group F, member 2 |
| NCKAP1 | 1.01E-19 | -16.626 | NCK-associated protein 1 |
| CTBP2 | 1.01E-19 | -16.571 | C-terminal binding protein 2 |
| CD151 | 6.25E-19 | -15.802 | CD151 antigen |
| DAG1 | 7.64E-18 | -14.838 | dystroglycan 1 (dystrophin-associated glycoprotein 1) |
| RNF11 | 6.60E-17 | -14.043 | ring finger protein 11 |
| PHTF2 | 2.05E-16 | 13.573 | putative homeodomain transcription factor 2 |
| PFN2 | 8.42E-16 | -13.064 | profilin 2 |
| CD63 | 8.42E-16 | -13.042 | CD63 antigen (melanoma 1 antigen) |
| TPM1 | 2.38E-15 | -12.680 | tropomyosin 1 (alpha) |
| CAPNS1 | 1.06E-14 | -12.186 | calpain, small subunit 1 |
| YES1 | 1.09E-14 | -12.152 | v-yes-1 Yamaguchi sarcoma viral oncogene homolog 1 |
| OCRL | 1.09E-14 | -12.128 | oculocerebrorenal syndrome of Lowe |
| RB1 | 1.31E-14 | 12.037 | retinoblastoma 1 (including osteosarcoma) |
| LAPTM4A | 1.31E-14 | -12.031 | lysosomal-associated protein transmembrane 4 alpha |
| RDX | 2.55E-14 | -11.808 | radixin |
| PTK2 | 2.75E-14 | -11.768 | PTK2 protein tyrosine kinase 2 |
| ID1 | 4.99E-14 | -11.571 | inhibitor of DNA binding 1, dominant negative helix-loop-helix protei |
| PTMS | 6.86E-14 | -11.459 | parathymosin |
| CTNNA1 | 1.06E-13 | -11.304 | catenin (cadherin-associated protein), alpha 1, 102kDa |
| FLJ11021 | 1.34E-13 | 11.217 | similar to splicing factor, arginine/serine-rich 4 |
| CTNND1 | 2.13E-13 | -11.065 | catenin (cadherin-associated protein), delta 1 |
| HSPB1 | 2.40E-13 | -11.018 | heat shock 27kDa protein 1 |
| PRPF4B | 2.62E-13 | 10.980 | PRP4 pre-mRNA processing factor 4 homolog B (yeast) |
| TCTEL1 | 3.46E-13 | -10.887 | t-complex-associated-testis-expressed 1-like 1 |
| SEP-10 | 1.28E-12 | -10.470 | septin 10 |
| SEC31L1 | 1.28E-12 | 10.464 | SEC31-like 1 (S. cerevisiae) |
| PHLDA2 | 1.41E-12 | -10.427 | pleckstrin homology-like domain, family A, member 2 |
| POU2AF1 | 1.47E-12 | 10.405 | POU domain, class 2, associating factor 1 |
| PTPRF | 1.88E-12 | -10.326 | protein tyrosine phosphatase, receptor type, F |
| CXX1 | 5.56E-12 | -10.005 | CAAX box 1 |
| PLCG2 | 6.82E-12 | 9.934 | phospholipase C, gamma 2 (phosphatidylinositol-specific) |
| CLPX | 6.82E-12 | 9.931 | ClpX caseinolytic protease X homolog (E. coli) |
| KIAA1078 | 6.95E-12 | -9.917 | KIAA1078 protein |
| SP140 | 7.01E-12 | 9.909 | SP140 nuclear body protein |
| APP | 7.84E-12 | -9.869 | amyloid beta (A4) precursor protein (protease nexin-II, Alzheimer dis |
| CAPZA1 | 7.99E-12 | 9.857 | capping protein (actin filament) muscle Z-line, alpha 1 |
| SUZ12 | 1.02E-11 | 9.781 | suppressor of zeste 12 homolog (Drosophila) |
| APLP2 | 1.11E-11 | -9.751 | amyloid beta (A4) precursor-like protein 2 |
| DOCK2 | 1.11E-11 | 9.739 | dedicator of cytokinesis 2 |
| CCND1 | 1.11E-11 | -9.739 | cyclin D1 (PRAD1: parathyroid adenomatosis 1) |
| CNOT2 | 1.11E-11 | 9.732 | CCR4-NOT transcription complex, subunit 2 |
| KRT18 | 1.71E-11 | -9.603 | keratin 18 |
| SLC25A5 | 2.47E-11 | 9.487 | solute carrier family 25 (mitochondrial carrier; adenine nucleotide t |
| WWTR1 | 2.48E-11 | -9.477 | WW domain containing transcription regulator 1 |
| RPIA | 2.48E-11 | 9.475 | ribose 5-phosphate isomerase A (ribose 5-phosphate epimerase) |
| RHOC | 2.52E-11 | -9.465 | ras homolog gene family, member C |
| RASGRP1 | 2.58E-11 | 9.451 | RAS guanyl releasing protein 1 (calcium and DAG-regulated) |
| COX7A2L | 2.58E-11 | 9.448 | cytochrome c oxidase subunit VIIa polypeptide 2 like |
| POU4F1 | 2.61E-11 | 9.439 | POU domain, class 4, transcription factor 1 |
| C14orf2 | 2.83E-11 | 9.407 | chromosome 14 open reading frame 2 |
| GATA2 | 2.83E-11 | -9.406 | GATA binding protein 2 |
| PITPNM1 | 2.87E-11 | 9.397 | phosphatidylinositol transfer protein, membrane-associated 1 |
| GNA11 | 2.96E-11 | -9.384 | guanine nucleotide binding protein (G protein), alpha 11 (Gq |
| SLC35D2 | 2.98E-11 | -9.377 | solute carrier family 35, member D2 |
| BLMH | 3.10E-11 | 9.361 | bleomycin hydrolase |
| LOC92689 | 3.12E-11 | -9.355 | Hypothetical protein BC001096 |
| SFRS11 | 3.34E-11 | 9.331 | splicing factor, arginine/serine-rich 11 |
| IDH3A | 3.72E-11 | 9.297 | isocitrate dehydrogenase 3 (NAD+) alpha |
| CD53 | 3.79E-11 | 9.284 | CD53 antigen |
| CRLF3 | 3.98E-11 | 9.265 | cytokine receptor-like factor 3 |
| MDH1 | 4.09E-11 | 9.254 | malate dehydrogenase 1, NAD (soluble) |
| BSG | 5.35E-11 | -9.175 | basigin (OK blood group) |
| TCEAL4 | 5.35E-11 | -9.170 | transcription elongation factor A (SII)-like 4 |
| FCN3 | 5.88E-11 | 9.141 | ficolin (collagen/fibrinogen domain containing) 3 (Hakata antigen) |
| BNIP3 | 5.96E-11 | -9.132 | BCL2/adenovirus E1B 19kDa interacting protein 3 |
| IGFBP4 | 6.10E-11 | -9.122 | insulin-like growth factor binding protein 4 |
| SC5DL | 6.12E-11 | -9.117 | sterol-C5-desaturase (ERG3 delta-5-desaturase homolog, fungal |
| PL6 | 6.22E-11 | -9.109 | placental protein 6 |
| ABL1 | 6.54E-11 | -9.092 | v-abl Abelson murine leukemia viral oncogene homolog 1 |
| TCL1A | 8.21E-11 | 9.018 | T-cell leukemia/lymphoma 1A |
| OAZ2 | 8.31E-11 | -9.011 | ornithine decarboxylase antizyme 2 |
| RAB13 | 9.28E-11 | -8.977 | RAB13, member RAS oncogene family |
| TMSB4Y | 1.04E-10 | 8.938 | thymosin, beta 4, Y-linked |
| MTERF | 1.20E-10 | 8.895 | transcription termination factor, mitochondrial |
| EIF4G1 | 1.24E-10 | -8.884 | eukaryotic translation initiation factor 4 gamma, 1 |
| BTAF1 | 1.30E-10 | 8.867 | BTAF1 RNA polymerase II, B-TFIID transcription factor-associa |
| KIAA0125 | 1.32E-10 | 8.859 | KIAA0125 |
| EEF1B2 | 1.42E-10 | 8.835 | eukaryotic translation elongation factor 1 beta 2 |
| PCBD1 | 1.48E-10 | -8.821 | 6-pyruvoyl-tetrahydropterin synthase/dimerization cofactor of |
| KRTHB6 | 1.78E-10 | 8.765 | keratin, hair, basic, 6 (monilethrix) |
| C7orf23 | 1.86E-10 | 8.749 | chromosome 7 open reading frame 23 |
| DLD | 2.10E-10 | 8.713 | dihydrolipoamide dehydrogenase (E3 component of pyruvate dehydrogenas |
| PGRMC1 | 2.19E-10 | -8.694 | progesterone receptor membrane component 1 |
| SKP1A | 2.77E-10 | -8.626 | S-phase kinase-associated protein 1A (p19A) |
| PGK1 | 3.16E-10 | 8.588 | phosphoglycerate kinase 1 |
| PON2 | 3.97E-10 | -8.524 | paraoxonase 2 |
| ZNF157 | 4.05E-10 | 8.515 | zinc finger protein 157 (HZF22) |
| TNF | 4.64E-10 | 8.473 | tumor necrosis factor (TNF superfamily, member 2) |
| MT4 | 4.64E-10 | 8.471 | metallothionein IV |
| P2RX5 | 5.66E-10 | 8.415 | purinergic receptor P2X, ligand-gated ion channel, 5 |
| NR1H2 | 5.84E-10 | -8.403 | nuclear receptor subfamily 1, group H, member 2 |
| PFKFB2 | 6.12E-10 | 8.388 | 6-phosphofructo-2-kinase/fructose-2,6-biphosphatase 2 |
| HLA-DOB | 6.40E-10 | 8.373 | major histocompatibility complex, class II, DO beta |
| DNAJC8 | 6.85E-10 | 8.351 | DnaJ (Hsp40) homolog, subfamily C, member 8 |
| PRCP | 6.94E-10 | -8.346 | prolylcarboxypeptidase (angiotensinase C) |
| ZNF148 | 6.96E-10 | -8.342 | zinc finger protein 148 (pHZ-52) |
| PLD3 | 7.40E-10 | -8.323 | phospholipase D3 |
| SNTB2 | 7.59E-10 | -8.314 | syntrophin, beta 2 (dystrophin-associated protein A1, 59kDa, basic co |
| Cluster 4 |  |  |  |
| HPN | 9.30E-14 | 12.314 | hepsin (transmembrane protease, serine 1) |
| CAMKK2 | 2.41E-13 | 11.806 | calcium/calmodulin-dependent protein kinase kinase 2, beta |
| ARFIP2 | 3.45E-13 | 11.575 | ADP-ribosylation factor interacting protein 2 (arfaptin 2) |
| ATP6V1G1 | 1.53E-12 | 11.041 | ATPase, H+ transporting, lysosomal 13kDa, V1 subunit |
| LU | 2.71E-12 | 10.807 | Lutheran blood group (Auberger b antigen included) |
| PPM1H | 4.69E-12 | 10.537 | protein phosphatase 1H (PP2C domain containing) |
| ENTPD6 | 4.69E-12 | 10.514 | ectonucleoside triphosphate diphosphohydrolase 6 (putative fu |
| KIAA0152 | 4.69E-12 | 10.509 | KIAA0152 |
| BICD1 | 5.30E-12 | 10.440 | bicaudal D homolog 1 (Drosophila) |
| ABCC4 | 9.07E-12 | 10.219 | ATP-binding cassette, sub-family C (CFTR/MRP), member 4 |
| PDLIM5 | 9.07E-12 | 10.203 | PDZ and LIM domain 5 |
| MCCC2 | 9.07E-12 | 10.201 | methylcrotonoyl-Coenzyme A carboxylase 2 (beta) |
| P4HB | 2.28E-11 | 9.914 | procollagen-proline, 2-oxoglutarate 4-dioxygenase (proline 4-hydroxyl |
| AMACR | 2.70E-11 | 9.845 | alpha-methylacyl-CoA racemase |
| TEGT | 2.99E-11 | 9.797 | testis enhanced gene transcript (BAX inhibitor 1) |
| SYNGR2 | 3.54E-11 | 9.713 | synaptogyrin 2 |
| OACT2 | 4.70E-11 | 9.616 | O-acyltransferase (membrane bound) domain containing 2 |
| GRP58 | 7.59E-11 | 9.466 | glucose regulated protein, 58kDa |
| KIAA0268 | 8.01E-11 | 9.435 | /// LOC440751 C219-reactive peptide /// similar to C219-reactive pe |
| LDHB | 8.01E-11 | -9.422 | lactate dehydrogenase B |
| TSPAN1 | 9.32E-11 | 9.367 | tetraspan 1 |
| DHRS7 | 1.26E-10 | 9.270 | dehydrogenase/reductase (SDR family) member 7 |
| PGAM1 | 1.39E-10 | -9.219 | phosphoglycerate mutase 1 (brain) |
| RAP1GA1 | 2.57E-10 | 9.016 | RAP1, GTPase activating protein 1 |
| KIAA0251 | 6.17E-10 | 8.764 | KIAA0251 protein |
| ERBB3 | 1.00E-09 | 8.621 | v-erb-b2 erythroblastic leukemia viral oncogene homolog 3 (avian) |
| SHANK2 | 1.00E-09 | 8.614 | SH3 and multiple ankyrin repeat domains 2 |
| SLC9A6 | 1.07E-09 | -8.587 | solute carrier family 9 (sodium/hydrogen exchanger), isoform |
| HIST1H2AD | 1.34E-09 | 8.512 | /// HIST1H3D histone 1, H2ad /// histone 1, H3d |
| BBS4 | 1.34E-09 | 8.508 | Bardet-Biedl syndrome 4 |
| MMD | 1.92E-09 | -8.387 | monocyte to macrophage differentiation-associated |
| KLK2 | 1.92E-09 | 8.374 | kallikrein 2, prostatic |
| LRIG1 | 1.92E-09 | 8.371 | leucine-rich repeats and immunoglobulin-like domains 1 |
| REPS2 | 1.92E-09 | 8.371 | RALBP1 associated Eps domain containing 2 |
| PDE3B | 2.34E-09 | 8.309 | phosphodiesterase 3B, cGMP-inhibited |
| PTPRN2 | 3.31E-09 | 8.202 | protein tyrosine phosphatase, receptor type, N polypeptide 2 |
| HGD | 3.31E-09 | 8.195 | homogentisate 1,2-dioxygenase (homogentisate oxidase) |
| GTF3C1 | 3.31E-09 | 8.195 | general transcription factor IIIC, polypeptide 1, alpha 220kDa |
| IQGAP2 | 3.41E-09 | 8.181 | IQ motif containing GTPase activating protein 2 |
| C21orf5 | 4.72E-09 | 8.081 | chromosome 21 open reading frame 5 |
| ASAHL | 4.72E-09 | 8.079 | N-acylsphingosine amidohydrolase (acid ceramidase)-like |
| NEDD4L | 4.72E-09 | 8.074 | neural precursor cell expressed, developmentally down-regulat |
| PRSS8 | 8.92E-09 | 7.880 | protease, serine, 8 (prostasin) |
| CPD | 8.92E-09 | 7.877 | carboxypeptidase D |
| ESRRG | 8.92E-09 | 7.873 | estrogen-related receptor gamma |
| FAAH | 9.41E-09 | 7.850 | fatty acid amide hydrolase |
| C9orf61 | 9.54E-09 | 7.839 | chromosome 9 open reading frame 61 |
| FPR1 | 9.54E-09 | 7.835 | formyl peptide receptor 1 |
| GREB1 | 9.55E-09 | 7.830 | GREB1 protein |
| KLK3 | 1.07E-08 | 7.793 | kallikrein 3, (prostate specific antigen) |
| PHF8 | 1.07E-08 | 7.790 | PHD finger protein 8 |
| SERP1 | 1.11E-08 | 7.776 | stress-associated endoplasmic reticulum protein 1 |
| AUH | 1.56E-08 | 7.679 | AU RNA binding protein/enoyl-Coenzyme A hydratase |
| ATP6AP1 | 1.58E-08 | 7.670 | ATPase, H+ transporting, lysosomal accessory protein 1 |
| HIST1H2BD | 1.60E-08 | 7.660 | histone 1, H2bd |
| LOC51760 | 1.60E-08 | 7.658 | B/K protein |
| HIST2H2BE | 1.60E-08 | 7.653 | histone 2, H2be |
| FLJ20323 | 1.60E-08 | 7.650 | hypothetical protein FLJ20323 |
| HIST2H2AA | 1.76E-08 | 7.617 | histone 2, H2aa |
| USP33 | 1.76E-08 | 7.612 | ubiquitin specific protease 33 |
| GAA | 1.76E-08 | 7.607 | glucosidase, alpha; acid (Pompe disease, glycogen storage disease typ |
| ABAT | 1.76E-08 | 7.606 | 4-aminobutyrate aminotransferase |
| TNFSF10 | 1.79E-08 | 7.593 | tumor necrosis factor (ligand) superfamily, member 10 |
| TPD52L2 | 1.90E-08 | -7.573 | tumor protein D52-like 2 |
| ANP32E | 1.93E-08 | -7.565 | acidic (leucine-rich) nuclear phosphoprotein 32 family, member E |
| ARFRP1 | 1.96E-08 | 7.558 | ADP-ribosylation factor related protein 1 |
| BNIP2 | 1.96E-08 | -7.548 | BCL2/adenovirus E1B 19kDa interacting protein 2 |
| UBE3C | 1.96E-08 | -7.548 | ubiquitin protein ligase E3C |
| RDH11 | 2.11E-08 | 7.520 | retinol dehydrogenase 11 (all-trans and 9-cis) |
| KIF5C | 2.11E-08 | 7.515 | kinesin family member 5C |
| PTPN11 | 2.11E-08 | -7.514 | protein tyrosine phosphatase, non-receptor type 11 (Noonan syndrome 1 |
| H2AFZ | 2.21E-08 | -7.498 | H2A histone family, member Z |
| TFRC | 2.26E-08 | -7.488 | transferrin receptor (p90, CD71) |
| CTPS | 2.27E-08 | -7.483 | CTP synthase |
| DJ167A19.1 | 2.27E-08 | 7.482 | hypothetical protein DJ167A19.1 |
| PFKP | 2.34E-08 | -7.470 | phosphofructokinase, platelet |
| AIM1 | 2.40E-08 | 7.460 | absent in melanoma 1 |
| RGL2 | 2.56E-08 | 7.440 | ral guanine nucleotide dissociation stimulator-like 2 |
| ADCY3 | 2.89E-08 | -7.403 | adenylate cyclase 3 |
| MGC15523 | 2.97E-08 | 7.393 | hypothetical protein MGC15523 |
| TPD52 | 3.13E-08 | 7.376 | tumor protein D52 |
| PPAP2A | 3.13E-08 | 7.374 | phosphatidic acid phosphatase type 2A |
| CANX | 3.43E-08 | 7.340 | calnexin |
| ZNF217 | 3.43E-08 | 7.333 | zinc finger protein 217 |
| COG5 | 3.43E-08 | 7.333 | component of oligomeric golgi complex 5 |
| LPIN1 | 3.43E-08 | -7.331 | lipin 1 |
| BAIAP2 | 3.43E-08 | 7.330 | BAI1-associated protein 2 |
| ZNF652 | 3.47E-08 | 7.324 | zinc finger protein 652 |
| SEC23A | 3.76E-08 | -7.296 | Sec23 homolog A (S. cerevisiae) |
| CPE | 3.79E-08 | 7.292 | carboxypeptidase E |
| SEL1L | 3.79E-08 | 7.289 | sel-1 suppressor of lin-12-like (C. elegans) |
| ACSL3 | 3.79E-08 | 7.285 | acyl-CoA synthetase long-chain family member 3 |
| MUT | 3.95E-08 | 7.269 | methylmalonyl Coenzyme A mutase |
| DDB2 | 4.01E-08 | -7.262 | damage-specific DNA binding protein 2, 48kDa |
| ATP2C1 | 4.06E-08 | 7.254 | ATPase, Ca++ transporting, type 2C, member 1 |
| ICA1 | 4.14E-08 | 7.247 | islet cell autoantigen 1, 69kDa |
| SAT | 4.37E-08 | 7.229 | spermidine/spermine N1-acetyltransferase |
| KIAA1002 | 4.63E-08 | 7.211 | KIAA1002 protein |
| OS-9 | 4.68E-08 | 7.207 | amplified in osteosarcoma |
| Cluster 5 |  |  |  |
| PAK3 | 1.34E-06 | 7.710 | P21 (CDKN1A)-activated kinase 3 |
| NME1 | 1.91E-06 | -7.427 | non-metastatic cells 1, protein (NM23A) expressed in |
| EYA1 | 4.79E-06 | 7.070 | eyes absent homolog 1 (Drosophila) |
| ACTC | 5.04E-06 | 6.979 | actin, alpha, cardiac muscle |
| STAC | 5.13E-06 | 6.914 | SH3 and cysteine rich domain |
| ATP2A2 | 6.42E-06 | 6.804 | ATPase, Ca++ transporting, cardiac muscle, slow twitch 2 |
| CALM1 | 6.76E-06 | 6.726 | calmodulin 1 (phosphorylase kinase, delta) |
| PPP1CA | 6.76E-06 | -6.711 | protein phosphatase 1, catalytic subunit, alpha isoform |
| TRPC1 | 8.07E-06 | 6.633 | transient receptor potential cation channel, subfamily C, member 1 |
| UCK2 | 1.03E-05 | -6.539 | uridine-cytidine kinase 2 |
| EIF3S2 | 1.04E-05 | -6.510 | eukaryotic translation initiation factor 3, subunit 2 beta, 36kDa |
| DMPK | 1.10E-05 | 6.466 | dystrophia myotonica-protein kinase |
| GPRASP1 | 1.10E-05 | 6.448 | G protein-coupled receptor associated sorting protein 1 |
| HADH2 | 1.10E-05 | -6.400 | hydroxyacyl-Coenzyme A dehydrogenase, type II |
| OLFML2A | 1.10E-05 | 6.399 | olfactomedin-like 2A |
| ABCF3 | 1.10E-05 | -6.376 | ATP-binding cassette, sub-family F (GCN20), member 3 |
| DSCR1L1 | 1.10E-05 | 6.370 | Down syndrome critical region gene 1-like 1 |
| PPIA | 1.10E-05 | -6.362 | peptidylprolyl isomerase A (cyclophilin A) |
| EIF4A1 | 1.13E-05 | -6.340 | eukaryotic translation initiation factor 4A, isoform 1 |
| PGM5 | 1.47E-05 | 6.255 | phosphoglucomutase 5 |
| DDHD2 | 2.39E-05 | 6.085 | DDHD domain containing 2 |
| PRDX4 | 2.41E-05 | -6.070 | peroxiredoxin 4 |
| MRPL12 | 2.51E-05 | -6.047 | mitochondrial ribosomal protein L12 |
| CLU | 2.68E-05 | 6.019 | clusterin (complement lysis inhibitor, SP-40,40, sulfated glycoprotei |
| DDX49 | 2.89E-05 | -5.989 | DEAD (Asp-Glu-Ala-Asp) box polypeptide 49 |
| LMO4 | 3.43E-05 | 5.923 | LIM domain only 4 |
| SH3BGR | 3.43E-05 | 5.922 | SH3 domain binding glutamic acid-rich protein |
| DPM2 | 3.48E-05 | -5.909 | dolichyl-phosphate mannosyltransferase polypeptide 2, regulatory subu |
| CCT3 | 4.36E-05 | -5.830 | chaperonin containing TCP1, subunit 3 (gamma) |
| C9orf3 | 4.53E-05 | 5.811 | chromosome 9 open reading frame 3 |
| ACLY | 4.87E-05 | -5.782 | ATP citrate lyase |
| PMM2 | 4.99E-05 | -5.768 | phosphomannomutase 2 |
| AK2 | 5.08E-05 | -5.755 | adenylate kinase 2 |
| CACNA1C | 5.34E-05 | 5.727 | calcium channel, voltage-dependent, L type, alpha 1C subunit |
| KCNAB1 | 5.38E-05 | 5.717 | potassium voltage-gated channel, shaker-related subfamily, beta membe |
| ROR2 | 5.43E-05 | 5.708 | receptor tyrosine kinase-like orphan receptor 2 |
| YWHAQ | 5.44E-05 | -5.701 | tyrosine 3-monooxygenase/tryptophan 5-monooxygenase activation protei |
| PSMB7 | 5.92E-05 | -5.664 | proteasome (prosome, macropain) subunit, beta type, 7 |
| COL4A6 | 5.98E-05 | 5.655 | collagen, type IV, alpha 6 |
| ADAM22 | 6.42E-05 | 5.625 | A disintegrin and metalloproteinase domain 22 |
| FLNA | 6.42E-05 | 5.623 | filamin A, alpha (actin binding protein 280) |
| SEC61G | 6.45E-05 | -5.616 | Sec61 gamma subunit |
| ALG8 | 6.52E-05 | -5.603 | asparagine-linked glycosylation 8 homolog (yeast, alpha-1,3-g |
| MRPL3 | 6.52E-05 | -5.593 | mitochondrial ribosomal protein L3 |
| PDCD6 | 6.52E-05 | -5.590 | programmed cell death 6 |
| KLHL21 | 6.52E-05 | 5.589 | kelch-like 21 (Drosophila) |
| NME2 | 6.52E-05 | -5.585 | non-metastatic cells 2, protein (NM23B) expressed in |
| HSPE1 | 6.99E-05 | -5.559 | heat shock 10kDa protein 1 (chaperonin 10) |
| GATM | 6.99E-05 | 5.555 | glycine amidinotransferase (L-arginine:glycine amidinotransferase) |
| SLC7A1 | 7.81E-05 | -5.519 | solute carrier family 7 (cationic amino acid transporter, y+ |
| ZFP36L1 | 8.55E-05 | 5.490 | zinc finger protein 36, C3H type-like 1 |
| TLN1 | 8.84E-05 | 5.476 | talin 1 |
| TXNDC9 | 9.04E-05 | -5.465 | thioredoxin domain containing 9 |
| DAAM2 | 9.27E-05 | 5.452 | dishevelled associated activator of morphogenesis 2 |
| APRT | 9.27E-05 | -5.446 | adenine phosphoribosyltransferase |
| OGG1 | 9.27E-05 | -5.443 | 8-oxoguanine DNA glycosylase |
| PPP1R12A | 9.57E-05 | 5.430 | protein phosphatase 1, regulatory (inhibitor) subunit 12A |
| CYLD | 9.57E-05 | 5.426 | Cylindromatosis (turban tumor syndrome) |
| KIAA1648 | 1.01E-04 | 5.407 | KIAA1648 protein |
| TXNDC7 | 1.07E-04 | -5.381 | thioredoxin domain containing 7 (protein disulfide isomerase) |
| PAICS | 1.07E-04 | -5.380 | phosphoribosylaminoimidazole carboxylase, phosphoribosylaminoimidazol |
| RBPMS | 1.07E-04 | 5.377 | RNA binding protein with multiple splicing |
| CBX7 | 1.14E-04 | 5.351 | chromobox homolog 7 |
| ATP6V0B | 1.14E-04 | -5.348 | ATPase, H+ transporting, lysosomal 21kDa, V0 subunit c'' |
| ANAPC5 | 1.14E-04 | -5.342 | anaphase promoting complex subunit 5 |
| ACTG1 | 1.14E-04 | -5.340 | actin, gamma 1 |
| TBL1X | 1.15E-04 | 5.334 | transducin (beta)-like 1X-linked |
| ADAMTSL3 | 1.17E-04 | 5.324 | ADAMTS-like 3 |
| SVIL | 1.47E-04 | 5.257 | supervillin |
| GSTM5 | 1.52E-04 | 5.245 | glutathione S-transferase M5 |
| DMD | 1.55E-04 | 5.233 | dystrophin (muscular dystrophy, Duchenne and Becker types) |
| UBE2N | 1.55E-04 | -5.232 | ubiquitin-conjugating enzyme E2N (UBC13 homolog, yeast) |
| HEPH | 1.71E-04 | 5.199 | hephaestin |
| RPN2 | 2.05E-04 | -5.145 | ribophorin II |
| RRAS | 2.14E-04 | 5.129 | related RAS viral (r-ras) oncogene homolog |
| TMEM4 | 2.18E-04 | -5.122 | transmembrane protein 4 |
| MRPS12 | 2.37E-04 | -5.094 | mitochondrial ribosomal protein S12 |
| CAMK2G | 2.54E-04 | 5.072 | calcium/calmodulin-dependent protein kinase (CaM kinase) II g |
| TCEAL1 | 2.65E-04 | 5.056 | transcription elongation factor A (SII)-like 1 |
| ANKS1 | 2.89E-04 | 5.028 | ankyrin repeat and sterile alpha motif domain containing 1 |
| PPP2R1B | 2.91E-04 | -5.020 | protein phosphatase 2 (formerly 2A), regulatory subunit A (PR 65), beta isofo |
| FHL1 | 3.19E-04 | 4.991 | four and a half LIM domains 1 |
| JTV1 | 3.22E-04 | -4.984 | JTV1 gene |
| SPG20 | 3.29E-04 | 4.975 | spastic paraplegia 20, spartin (Troyer syndrome) |
| C16orf45 | 3.32E-04 | 4.970 | chromosome 16 open reading frame 45 |
| FGFR2 | 3.37E-04 | 4.962 | fibroblast growth factor receptor 2 (bacteria-expressed kinase, kerat |
| SMTN | 3.41E-04 | 4.955 | smoothelin |
| GCS1 | 3.41E-04 | -4.953 | glucosidase I |
| MEIS2 | 3.67E-04 | 4.925 | Meis1, myeloid ecotropic viral integration site 1 homolog 2 ( |
| SEC61B | 3.67E-04 | -4.922 | Sec61 beta subunit |
| PYCR1 | 3.67E-04 | -4.920 | pyrroline-5-carboxylate reductase 1 |
| MYH3 | 3.79E-04 | 4.908 | myosin, heavy polypeptide 3, skeletal muscle, embryonic |
| CD200 | 3.86E-04 | 4.900 | CD200 antigen |
| PPP1R12B | 3.95E-04 | 4.891 | protein phosphatase 1, regulatory (inhibitor) subunit |
| ATP5J2 | 4.01E-04 | -4.880 | ATP synthase, H+ transporting, mitochondrial F0 complex, subu |
| NDUFV2 | 4.01E-04 | -4.881 | NADH dehydrogenase (ubiquinone) flavoprotein 2, 24kDa |
| DIXDC1 | 4.09E-04 | 4.867 | DIX domain containing 1 |
| ZMPSTE24 | 4.09E-04 | -4.867 | zinc metallopeptidase (STE24 homolog, yeast) |
| POLD2 | 4.43E-04 | -4.840 | polymerase (DNA directed), delta 2, regulatory subunit 50kDa |
